# The Fabaceae in Northeastern Mexico (Subfamily Papilionoideae, Tribes Amorpheae, Brongniartieae, and Dalbergieae)

**DOI:** 10.3390/plants14050789

**Published:** 2025-03-04

**Authors:** Eduardo Estrada Castillón, José Ángel Villarreal Quintanilla, Juan Antonio Encina Domínguez, Arturo Mora Olivo, Jaime Sánchez Salas, Gisela Muro Pérez, Eduardo Alanís Rodríguez, Renata Aidé Valdés Alameda, Nelly Sandoval Mata, Gilberto Ocampo

**Affiliations:** 1 Facultad de Ciencias Forestales, Universidad Autónoma de Nuevo Léon, Linares 67700, Mexico; aeduardoestradac@prodigy.net.mx (E.E.C.); eduardo.alanisrd@uanl.edu.mx (E.A.R.); renatavamalmeda@gmail.com (R.A.V.A.); 2 Departamento de Botánica, Universidad Autónoma Agraria Antonio Narro, Saltillo 25315, Mexico; javillarreal00@hotmail.com (J.Á.V.Q.); jaencinad@gmail.com (J.A.E.D.); 3 Instituto de Ecología Aplicada, Universidad Autónoma de Tamaulipas, Ciudad Victoria 87019, Mexico; amorao@uat.edu.mx; 4 Facultad de Ciencias Biológicas, Universidad Juárez de Estado de Durango, Gómez Palacio 35010, Mexico; jsanchez@ujed.mx (J.S.S.); giselamuro@ujed.mx (G.M.P.); 5 Centro Universitario de Ciencias Biológicas y Agropecuarias, Universidad de Guadalajara, Zapopan 44600, Mexico; tecoatlayupeuh.sandoval0453@alumnos.udg.mx; 6 Centro de Ciencias Básicas, Departamento de Biología, Universidad Autónoma de Aguascalientes, Aguascalientes 20100, Mexico

**Keywords:** Amorpheae, Brongniarteae, Dalbergieae, diversity, Fabaceae, Lotoideae, northeastern Mexico, species richness, taxonomy

## Abstract

A compendium of the legumes of the subfamily Papilionoideae, tribes Amorpheae, Brongniarteae, and Dalbergieae in northeastern Mexico is presented for the first time, including changes in their botanical nomenclature within tribes and genera. Based on recently published studies, the taxonomic limits of several genera and new ones segregated such as *Marina* and *Ctenodon* are clarified and included. Based mainly on fieldwork over the past 40 years, as well as reviewing specimens in national and international herbaria, we show the total diversity of legumes of the subfamily Papilionoideae, tribes Amorpheae, Brongniarteae, and Dalbergieae. The three tribes include 16 genera and 75 species. Tribe Amorpheae comprises five genera (*Amorpha*, *Dalea*, *Eysenhardtia*, *Marina*, and *Psorothamnus*) and forty-three species; tribe Brongniartieae comprises two genera *Brongniartia* and *Harpalyce*) and eight species; and tribe Dalbergieae comprises nine genera (*Aeschynomene*, *Amicia*, *Arachis*, *Ctneodon*, *Dalbergia*, *Diphysa*, *Nissolia*, *Stylosanthes*, and *Zornia*) and twenty-four species. *Dalea* is by far the genus with the highest number of species and infraspecific categories, as well as in a number of endemisms because 17 (51%) of them are endemic to Mexico, and six of them are endemic to the northeastern part of the country. Of the 13 species of *Eysenhardtia* present in Mexico, 31% of them reach the northeast region and three of them are exclusive to this region. There are no species of the Brongniartieae and Dalbergieae tribes endemic to northeastern Mexico, but 10 of their species are endemic to Mexico.

## 1. Introduction

The Fabaceae (Leguminosae) represents one of the most economically and ecologically important groups of flowering plants [1] and is one with the highest species richness [2], comprising about 770 genera and 19,500 species [1]. Among the economically important species, beans (*Phaseolus vulgaris*), lentils (*Lens culinaris*), chickpeas (*Cicer arietinum*), peas (*Pisum sativum*), beans (*Vicia faba*), peanuts (*Arachis hypogaea*), and soybeans (*Glycine max*) stand out as an important part of the diet of millions of people. From an ecological point of view, the Fabaceae play a leading role in many of the planet’s biotic communities, being an important part of the vegetation in tropical, subtropical, and desert areas, where countless genera are the dominant elements in diversity, density, and coverage, such as *Acacia*, *Albizia*, *Bauhinia*, *Desmodium*, *Ebenopsis*, *Erythrina*, *Harpalyce*, *Havardia*, *Indigofera*, *Inga*, *Lonchocarpus*, *Neltuma*, *Senegalia*, and *Vachellia*. Many legume species are capable of fixing atmospheric nitrogen [3], an important aspect of agriculture, because nitrogen is an essential nutrient for plant development. Legumes are characterized by being multifunctional in terms of uses, including as a source of charcoal [4], firewood [5], wood [6], medicinal purposes [7], ornamental uses [8], chemical compounds [9], and tanning [10,11].

Molecular biology applied to plant systematics has revolutionized the classification of numerous taxa, merging or segregating families, subfamilies, tribes, genera, and species. The Fabaceae are one of the groups of flowering plants that have undergone substantial changes in their taxonomy with the application of these techniques. Since 2000, a large number of scientific articles have been published that report these changes [12,13,14,15,16,17,18,19,20,21,22,23,24,25]. Since 2017, six subfamilies (Caesalpinioideae, Cercidoideae, Detarioideae, Dialioideae, Duparquetioideae, and Papilionoideae) have been recognized as part of the Fabaceae [25], leaving behind the classic recognition of the three classic ones (Mimosoideae, Caesalpinioideae, and Papilionoideae).

The phylogenetic classification of the subfamily Papilionoideae has been modified over time [25,26]. Since 2013, this subfamily has been subject to nomenclature changes following phylogenetic consideration; however, there are still certain groups of plants whose phylogenetic relationships remain unresolved [15,25,26,27].

Mexico is a country rich in legume species [11,28] because approximately 1900 of them have been recorded [28], almost half of which are endemic. In northeastern Mexico, the presence of a high number of species has been reported, where the number of taxa of the subfamily Papilionoideae stands out [11].

The tribes Amorpheae, Brongniartieae, and Dalbergieae are members of the subfamily Papilionoideae [1]. The tribe Amorpheae is a group of plants native to the Americas [29], where several of its distinctive characteristics are the presence of oil glands on stems, leaves, inflorescences, and sometimes flowers. This tribe includes approximately 240 [29,30]–248 species [1]. The tribe Brongniartieae comprises herbaceous, subshrubs, shrubs, or tree species with multifoliate gland-dotted leaves, and sometimes with very large stipules, bilabiate or 5-toothed calyx, and 1-several seeds, dehiscent, and sometimes explosive fruits. This tribe includes 10 genera and approximately 152 species [31]. The tribe Dalbergieae includes trees, shrubs, or woody lianas sometimes with appendiculate stipules below the point of attachment, leaves paripinnate or imparipinnate, 1–many-foliolate, bracts similar to stipules or large and circular, often enclosing flowers and fruit, calyx with subequal lobes or teeth, or bilabiate, stamen monadelphous or diadelphous, and a flattened or drupe-like, indehiscent, or a loment or lomentaceous fruit. This tribe includes 49 genera and around 1300 species [32], more than twice as many genera (19), and more than six times as many species (300) are recognized [33].

Based on the new nomenclatural changes in the Fabaceae, as well as at the tribal and generic level within the subfamily Papilionoideae, it is necessary to update the nomenclature of those taxa that have undergone modifications, especially considering that there are no updated records of taxa of the subfamily Papilionoideae in northeastern Mexico. The goals of this work are to provide a study of the species richness of the tribes Amorpheae, Brongniartieae, and Dalbergieae of the subfamily Papilionoideae in northeastern Mexico, including new information regarding their most recent nomenclature, and to provide information about their distribution within the main plant communities found in the study area.

## 2. Results

### 2.1. Diversity of Species of the Tribes Amorpheae, Brongniartieae, and Dalbergieae of the Subfamily Papilionoideae and Growth Forms in Northeastern Mexico

**Figure 1:**
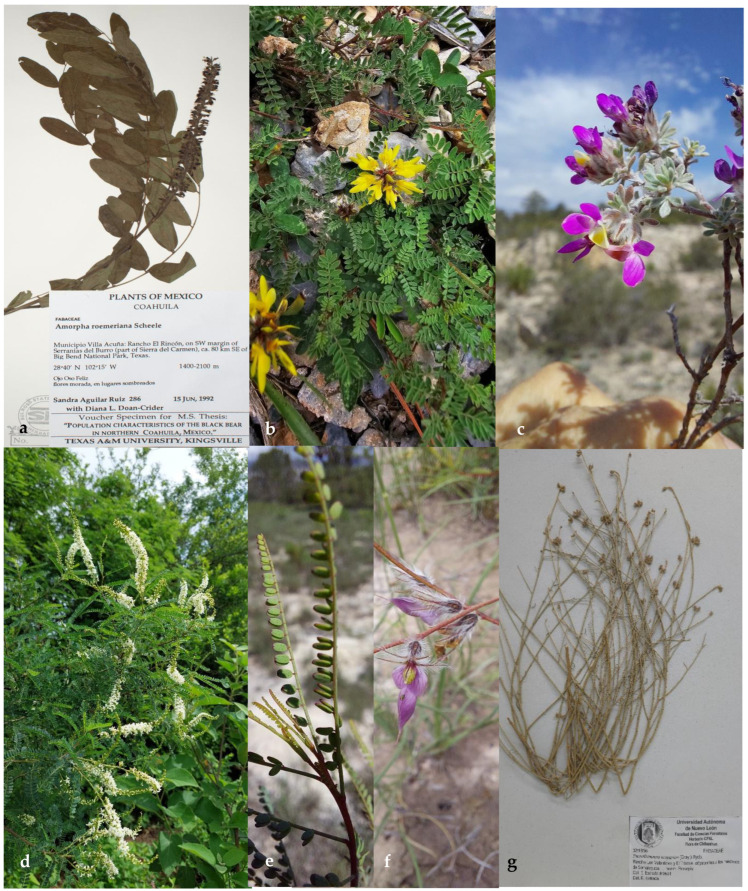
Species of the genera of tribe Amorpheae in northeastern Mexico. *Amorpha roemeriana* (**a**) (Herbarium specimen, deposited in the ANSM scientific collection), *Dalea lutea* var. *lutea* (**b**); *D. eriophylla* (**c**), *Eysenhardtia texana* (**d**), *Marina filiciformis* (**e**,**f**), and *Psorothamnus scoparius* (**g**) (Herbarium specimen, deposited in the ANSM scientific collection).

**Figure 2:**
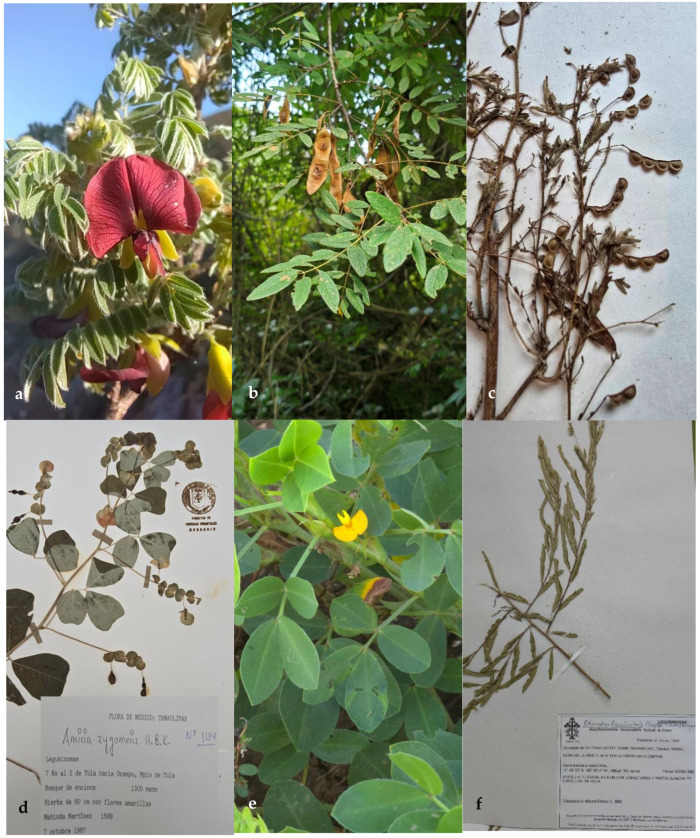
Species of the genera of tribe Brongniartieae in northeastern Mexico. *Brongniartia magnibracteata* (**a**), and *Harpalyce mexicana* (**b**). Species representative of the genera of tribe Dalbergieae in northeastern Mexico. *Aeschynomene villosa* var. *villosa* (**c**) (Herbarium specimen, deposited in the CFNL scientific collection), *Amicia zygomeris* (**d**) (Herbarium specimen, deposited in the CFNL scientific collection), *Arachis hypogaea* (**e**), and *Ctenodon fascicularis* (**f**) (Herbarium specimen, deposited in the CFNL scientific collection).

**Figure 3:**
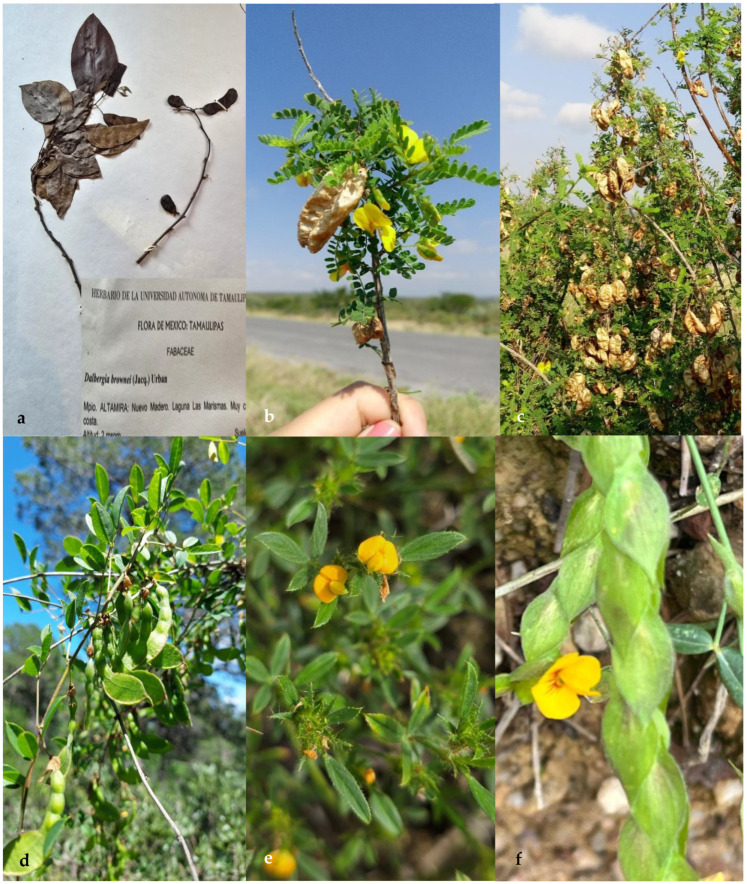
Species of the genera of tribe Dalbergieae in northeastern Mexico. *Dalbergia brownei* (**a**) (Herbarium specimen, deposited in the UAT scientific collection), *Diphysa microphylla* (**b**,**c**), *Nissolia platycalyx* (**d**), *Stylosanthes mexicana* (**e**), *Zornia reticulata* (**f**).

The tribes Amorpheae, Brongniarteae, and Dalbergieae of the subfamily Papilionoideae in northeastern Mexico comprise 16 genera and 75 species (Table 1). The tribe Amorpheae has five genera (*Amorpha*, *Dalea*, *Eysenhardtia*, *Marina*, and *Psorothamnus*) (Figure 1) and 43 species. The tribe Brongniartieae comprises the genera *Brongniartia* and *Harpalyce*, with eight species in total (Figure 2). Finally, the tribe Dalbergieae has nine genera (*Aeschynomene*, *Amicia*, *Arachis*, *Ctneodon*, *Dalbergia*, *Diphysa*, *Nissolia*, *Stylosanthes*, and *Zornia*), and 24 species (Figure 2 and Figure 3). *Dalea* is by far the genus with the highest number of species and infraspecific taxa, followed by *Nissolia*, *Brongniartia*, *Stylosanthes*, *Zornia*, and *Eysenhardtia*. There are genera with a high number of species in Mexico and other countries but are poorly represented in northeastern Mexico, such as *Amicia*, *Arachis*, *Amorpha*, *Ctenodon*, *Dalbergia*, and *Psorothamnus*.

The main growth forms are herbs (42 species and infraspecific taxa), shrubs (39 taxa), lianas, climbing plants (five taxa, *Nissolia* species), and trees (two taxa); the latter two belong to the genus *Harpalyce*. Almost half and half of the *Dalea* species are herbaceous and shrubs, respectively.

### 2.2. Endemism

Thirty-eight out of 74 (51%) that belong to tribes Amorpheae, Brongniatieae, and Dalbergieae in northeastern Mexico are endemic to the country (Table 2). *Dalea* is not only the genus with the highest species richness, but the one with the highest number of endemic taxa, because 17 (51%) of them are endemic to Mexico, and six of them are endemic to northeastern Mexico. Of the 13 species of *Eysenhardtia* present in Mexico, 31% of them reach the northeast region and three of them are exclusive to this part of the country. There are no species of the tribes Brongniartieae and Dalbergieae endemic to northeastern Mexico, but 10 of their species found in northeastern Mexico are endemic to the country.

### 2.3. Taxonomic Treatment

**Tribe Amorpheae** Barneby, Mem. New York Bot. Gard. 27: 4. 1977. Leguminosae tribus *Amorpheae* Borissova, Novit. Syst. PL Vase. 1964: 224. Fabaceae tribus *Daleeae* Hutchinson, Gen. Fl. PL, Dicotyl. 1: 413 {*“Daleae”*) 1964 (3 dec). Fabaceae tribus *Psoraleeae* Bentham emend. Hutchinson, Gen. Fl. PL, Dicotyl. 1: 414. (*Psoralieae*). 1964.

**Type**: *Amorpha* L., Sp. Pl.: 743. 1753.

Herbaceous, shrubs, rarely small trees. Leaves simple, trifoliolate, or pinnate. Aromatic (when bruised) glands (oil cells) are present in stems, leaves, calyx, and sometimes petals. Trichomes are simple and basifixed. Inflorescences in spikes, racemes, or heads (capitate). Flowers are white, yellow, pink, violet, purple, blue, bicolored, or more colors present in a flower. Ovules 1–2, rarely 3–7. Fruit mostly 1-seeded, rarely more (*Psorothamnus*), indehiscent, detaching along with the calyx. All taxa within this tribe are native to the American continent [29].

The tribe comprises eight genera and almost 240 [29,30]-248 species [1]. Five genera are recorded in northeastern Mexico, *Amorpha*, *Dalea*, *Eysenhardtia*, *Marina*, and *Psorothamnus.*
1A.Petals 5, one (banner) inserted on the hypanthium, the other four, the keel and the wings, inserted near the base, medially or apically on the stamens tube.21B.Petals 1 or 5, all of them, are always inserted at the base of the hypanthium.32A.Ovules 2; leaflets without adaxially or abaxially parallel white sinuous lines ascending from the middle vein; ribs of the calyx anastomosing or reaching the apex of each of the teeth, sometimes extending and forming a mucron. Plants with the inflorescence densely racemose, their flowers, close to each other, contiguous, separated from each other by a distance less than one time the size of the calyx, rarely more.***Dalea***2B.Ovules 1; leaflets with parallel white sinuous lines ascending from the middle vein adaxially (above) and sometimes abaxially; ribs of the calyx without anastomosing or without ever reaching the apex of each of the teeth or the plants with the inflorescences in lax racemes, its flowers distant to each other, separated from each other by a distance of 2–3 times the size of the calyx. ***Marina***3A.Flowers with only one petal (banner).***Amorpha***3B.Flowers with five petals.44A.Corolla papilionaceous; the keel blades closely overlapping on their outer margins, enclosing androecium; banner differentiated into a thin claw and a broad, basally cordate blade; the petals pink or purple.***Psorothamnus***4B.Corolla no papilionaceous; petals always free, not enclosing the androecium, all with the same shape; the petals white.***Eysenhardtia***

***Amorpha*** L., Sp. Pl. 743. 1753; Gen. Pl. ed. 5. 319. 1754. *Bonfidia* Necker, Elem. Bot. 3: 46. 1790.

**Type species:** *Amorpha fruticosa* L., Sp. Pl. 2: 713. 1753.

Subshrubs or shrubs. Leaves odd-pinnate. Leaflets 7–45 per leaf, margin smooth or crenulate, with or without glands. Inflorescences terminal, arranged in racemes or panicle-like. Bracts caducous. Flowers pedicellate. Calyx campanulate to funnel-shaped, its lobes short or long. Corolla with a single petal, the banner, inserted on the hypanthium, enveloping the stamens and gynoecium, clawed, the blade obovate to cordate, violet, blue, purple, or white, truncate to retuse. Stamens 10, basally monadelphous, the tube exerted beyond the calyx, the free filaments exerted beyond the calyx, and often the banner. Ovary 2-ovulate. Style exerted beyond the calyx and often the banner. Fruit indehiscent, straight to curved, compressed legume (pod), slightly equal to or longer than the calyx, with or without glands. Seed compressed.

A North American genus with 15 species [34], distributed from southern Canada to northern Mexico. An easily distinguishable genus from the rest of the tribe Amorpheae because its flowers have a single petal.

A single species recorded for northeastern Mexico, *A. roemeriana*.

***Amorpha roemeriana*** Scheele, Linnaea 21: 461. 1848. Basionym: *A. fruticosa* var. *subglabra* A. Gray, Boston Jour. Nat. Hist. 6: 174. 1850. *A. laevigata* var. *pubescens* A. Gray, Smithson. Contr. Knowl. 3: 49. 1852. *A. texana* Buckley, Proc. Acad. Nat. Sci. Philadelphia 1861: 452. 1862. *A. sublgabra* (A. Gray) Heller, Contr. Herb. Franklin-Marshall Coll. 1: 48. 1895. *A. texana* var. *mollis* Boynton, Biltmore Bot. Stud. 1: 139. 1902. *A. laevigata* var. *pubescens* f. *mollis* (Boynton) C.K. Schneid. Bot. Gaz. 43: 307. 1907. *A. texana* var. *glabrescens* Palmer, J. Arn. Arb. 12: 180. 1931.

**Type:** USA, Texas, Hays County, ash juniper woodland on extremely shallow stony clay loamon top of N to NW facing bluff of Fredericksburg Limestoneca. 40–50 ft above the S Bank of Blanco River, NW corner of Falls Ranch, ca. 4.5 air mi W of the junction of State Route 150 and Ranch road 2770 near Mountain City ca. 2.8 air mi SSE of the junction of State Route 150 and Ranch Road 3237 at Hays City at N 30°00′37.5″, W 097°58′01.1″, Mountaiun City Quadrangle, elev. 740–750 ft, 15-May 2009, *W.R. Carr*, *B. Johnson*, *T. Wendt 27810* (TEX) (Neotype: designated by Shannon C.K. Straub and J. L. Reveal in J. Bot. Res. Inst. Tex. 6(2): 339–340. 2012.

**Distinguishing features:** Shrub, 1–2.8 m tall. Stems and leaves densely pulverulent, occasionally glabrous, gland-dotted. Leaves 5–20 cm long. Leaflets 7–15, 1–3.5 cm long, oblong to elliptic, bicolored, smooth, or crenulate marginally. Inflorescences 5–18 cm long, in lax racemes. Calxy funnel-shaped, gland-dotted, apically, the lobes triangular-dentate, the abaxial one the longest. Banner suborbicular, 5–6 mm long, purple, emarginate. Stamens ca. 1 cm long. Ovary glabrous. Fruit 0.6–0.7 cm long, oblong, glabrous to strigillose, gland-dotted apically.

**Representative examined material:** Coahuila: 17-IX-1999, *Villarreal*, *Carranza*, *Rinskind*, *Henrickson*, *Wendt*, *Wagner 8795* (ANSM, TEX00253649!); 4-V-1981, *P.H. Riskind 2335a* et al. (UAT). 

**Distribution:** Endemic of Texas (USA) and northeastern Mexico, inhabiting oak-pine-maple forest in Mexico, 850–1570 m.

***Dalea*** Lucanus, Linnaei Opera Varia, 244. 1758.

**Type**: *Dalea cliffortiana* Willd., Hort. Cliff, t. 22. 1738.

Herbs or shrubs. Stems, leaves, and sometimes flowers with oily, aromatic glands. Leaves alternate, pinnate or trifoliolate. Bracts persistent or deciduous, glabrous or pubescent. Inflorescences, axillary or terminal, lax or compact, ovoid, cylindrical or cone-shaped spikes. Flowers papilionate, yellow, purple, blue, pink, white, or ochroleucus, sometimes the flowers bicolored, commonly the banner opening white or yellowish contrasting with some dark color of the other petals. Calyx campanulate, 5-toothed, 10-ribbed, these anastomosed at the apex of the tube or to the apex of the teeth, sometimes extending beyond these and forming a mucron or awn, the teeth with tiny additional teeth on the lateral edges. Corolla zygomorphic. Petals 5, the banner inserted on the hypanthium, the other four petals inserted basally, medially or apically in the staminal column; the two innermost ones form the keel, although they can be free from each other or imbricated (one on top of the other) and adherent on their external edges or also be valvately coherent with each other on their external edges; the other two petals form the wings, attached one each side of the keel. Stamens 5–10, united in a staminal column, connective often with a gland at the apex. Ovules 2, only 1 matures. Fruit small, enclosed by the calyx.

American genus with 161 [29]-175 species [35], distributed from southwestern Canada to Argentina; most of the taxa are found in Mexico (125), 85 of them are endemic species. Almost 95 species have been recorded from the northern region, inhabiting almost all plant communities, especially abundant in oak forest, conifer forest, and arid and semi-arid shrublands, some of them with restricted distribution and on gypsic soils.
1A.Stamens 521B.Stamens 7–1032A.Free filaments 1/3 or less of the length of the staminal column***D. emarginata***2B.Free filaments as long as the staminal column***D. multiflora***3A.Petals all free; filaments exserted from petals***D. lanata*** var. ***terminalis***3B.Keel-petals valvately fused at their outer margins or imbricately overlapping (one over the other) in their exterior edges; filaments almost always immersed in the keel 44AKeel-petals with their margins overlapping, one over the other (not fused at their outer margins); stamens immersed in the keel or exserted54B.Keel petals with their margins fused at their outer edges95A.Petals blue; hairs of the calyx 2.7–4.5 mm long***D. lachnostachys***5B.Petals bicolored, white, and crimson or lilac; hairs of the calyx 1–2.6 mm long66A.Stems and leaves glabrous; leaflets acute, linear-oblanceolate or elliptic; calyx sessile; keel 2.1–3.4 mm long ***D. cliffortiana***6B.Stems and leaves pubescent; leaflets obovate, obovate-oblong, obtuse, cordate, or truncate-cordate; calyx pedicellate; keel 4.5–7.1 mm long77A.Leaflets glabrous adaxially or hairy only on margin; pedicel glands relatively large, 0.5–0.7 mm long; calyx teeth 3.5 mm long or shorter; calyx glands absent or almost invisible***D. neomexicana*** var. ***megaladenia***7B.Leaflets pubescent adaxially and abaxially; pedicel glands relatively short, 0.15–0.4 mm long; calyx teeth 3.5–57 mm long; calyx glands small but visible and prominent88A.Leaflet margin entire, submarginal glands of 0.1–0.2 mm diameter***D. neomexicana*** var. ***longipila***8B.Leaflet margin strongly undulates due to the presence of submarginal glands, glands 0.2–0.3 mm in diameter***D. neomexicana*** var. ***neomexicana***9A.Dorsal teeth of the calyx hooked apically***D. scandens*** var. ***paucifolia***9B.Dorsal teeth of the calyx straight or slightly curved but never hooked1010A.Stems silvery-villous, arching and rooting at the tips forming colonies up to 1m in diameter***D. greggii***10B.Stems never arching or rooting at the tips to form colonies, or if stems arching then the plant glabrous and the bracts with verrucose glands1111A.Bracts with awned apex, equal to or longer than its body***D. obovatifolia*** var. ***obovatifolia***11B.Bracts acute or mucronate, without aristate apex or smaller than its body1212A.Herbaceous, annual plants1312B.Herbaceous perennial or shrub plants1513A.Petals yellow, early turning purple-brown or pink-brown: leaflets 1–5 pairs per leaf; racemes up to 1.6 cm long***D. brachystachya***13B.Petals bicolored, the banner opening white in part (the tip and basal lobes variably colored), but rubescent with age, wings, and keel petals dull pink, reddish-purple, magenta-purple or violet; leaflets 5–17 pairs per leaf; racemes 6–30 cm long1414A.Longest calyx tooth 1.4–4.3 mm long***D. foliolosa*** var. ***citrina***14B.Longest calyx tooth up to 1.3 mm long***D. foliolosa*** var. ***foliolosa***15A.Petals mainly yellow, light-yellow, sometimes turning pink or chocolate color with age1615B.Petals bicolored, the banner opening white or yellow, but rubescent with age, wings and keel petals white, pink, purple, magenta, or violet, never yellow3216A.Herbaceous, perennial, mostly gray or silvery pilose; calyx teeth 2.5 times longer than the tube1716B.Shrubs, erect shrubs, or if annual in appearance, the pubescence shaggy; calyx teeth less than 2.5 times longer than the tube 2517A.Leaves almost always 3-foliolate, rarely 5-foliolate1817B.Leaves mainly 5-foliolate, rarely 3 to 7-foliolate2118A.Leaflets linear-elliptic or linear-oblanceolate; pubescence appressed***D. luisana***18B.Leaflets obovate to elliptic-obovate; pubescence ascending, pilose1919A.Stems erect; calyx intercostal spaces with only one elongated gland ***D. boraginea***19B.Stems commonly prostrate; calyx intercostal spaces with a row of 3–4 small glands2020A.Banner blade up to 2.2 mm long, its claw 2–3 times longer than blade***D. laniceps***20B.Banner blade 2.6 mm long or longer, its claw 2 times longer than blade***D. prostrata***21A.Bracts 6–12 mm long; calyx commonly longer than 7 mm long2221B.Bracts 2.5–5.5 mm long; calyx commonly 6.5 mm long or shorter2322A.Banner 7.3–9.5 mm long; wings and keel petals inserted below middle of androecium ***D. wrightii***22B.Banner up to 6.3 mm long; wings and keel petals inserted above middle of androecium***D. parrasana***23A.Petals not turning red with age; the banner 6.2–8.6 mm long; stems commonly erect***D. aurea***23B.Petals turning red with age; the banner up to 5.5 mm long; stems commonly diffuse2424A.Inflorescence axis not visible due to the compactness of its flowers; bracts 1.2–2 mm wide***D. nana*** var. ***carnescens***24B.Inflorescence axis visible due to the laxity of its flowers, each of them separated by at least 1 mm of distance; bracts 2–4 mm wide***D. nana*** var. ***nana***25A.Stems shaggy pilose ***D. lutea*** var. ***lutea***25B.Stems glabrous2626A.Stems with abundant warty or verrucose glands, more evident in the young parts2726B.Stems without abundant warty or verrucose glands2927A.Pair of ventral calyx teeth free from each other, the space (sinus) between them not or barely shallower than the lateral sinuses***D. capitata*** var. ***capitata***27B.Pair of ventral calyx teeth fused, the space (sinus) between them shallower than the lateral sinuses2828A.Calyx glabrous externally, the orifice silky-villous***D. capitata*** var. ***pseudohospes***28B.Calyx silky-pilose externally***D. capitata*** var. ***lupinocalyx***29A.Leaflets softly puberulent***D. melantha*** var. ***pubens***29B.Leaflets glabrous3030A.Calyx teeth, shorter than tube, triangular, ventral pair wider than long, glabrate or tiny pilosulous; keel and wing petals inserted near middle of staminal tube, 3.4–5.4 mm from base; wing petal 6–7.5 mm long. ***D. hospes***30B.Calyx teeth, longer than tube, narrowly triangular-subulate to aristiform, always plumose; keel and wing petals inserted below middle of staminal tube, 0.8–1.7 mm from base; wing petal 4.8–5.2 mm long3131A.Leaflets 2–3 pairs per leaf; racemes loose, the flowers separated from each other by almost the same width of the calyx, the axis conspicuous***D. melantha*** var. ***berlandieri***31B.Leaflets 3–6 pairs per leaf, racemes more compact, the flowers, and calyces subcontiguous, the axis not conspicuous ***D. melantha*** var. ***melantha***32A.Leaves trifoliolate3332B.Leaves pinnate3433A.Leaflets obovate to oblanceolate, flattened or slightly revolute; inflorescences in distal compact, subcapitate racemes of 3–6 flowers, almost covered by distal leaves ***D. eriophylla***33B.Leaflets linear, tightly revolute; inflorescences distal of only one flower, barely visible, surrounded and almost covered by the distal leaves***D. uniflora***34A.Herbaceous perennial, stems not lignified at the base, they disappear after fruiting in the following season3534B.Shrubs, stems lignified at least at the base even when they measure 10–20 cm in height3735A.Calyx tube 3.3–4 mm long, pilose, the hairs 0.5–1 mm long; banner stained yellow in the center and with small glands present***D. lasiathera***35B.Calyx tube 2.6–3.3 mm long, plumose, the hairs 0.8–2.2 mm long; banner without stained yellow in the center and without small glands present3636A.Longest calyx tooth up to 3.9 mm long; tooth trichomes 1.4 mm long or shorter***D. pogonthera*** var. ***walkerae***36B.Longest calyx tooth 4 mm long or longer; tooth trichomes 1.5 mm long or longer***D. pogonathera*** var. ***pogonathera***37A.Dwarf shrubs up to 25 cm high, rooting adventitiously in the basal or apical parts of the stems3837B.Shrubs, erect 50 cm tall or taller3938A.Stems rhizomatous, rooting adventitiously in the basal parts; leaflets with tiny white papillae; calyx silky-pubescent; petals without tiny scattered glands***D. gypsophila***38B.Stems erect to suberect, rooting in the apical part of the branches; leaflets without papillae; petals without glands***D. radicans***39A.Leaflets glabrous4039B.Leaflets pubescent4240A.Stems with verrucose or tuberculated glands; calyx glabrous; calyx teeth shorter than the tube***D. frutescens***40B.Stems without verrucose or tuberculated glands; calyx pubescent; calyx teeth longer than the tube4141A.Flowers 2–9 in subcapitate racemes, raceme axis 9 mm or less; leaves 2.5–11 mm long with 3–7 pairs of unfolded leaflets; calyx teeth 4.5–8.5 mm long; calyx 7.4–16.2 mm long, its teeth 4.5–8.5 mm long***D. formosa***41B.Flowers 10 or more in more elongated racemes, raceme axis 5–15 mm long; leaves 10–16 mm long with 6–9 pairs of crowded, folded leaflets; calyx teeth 2.2–4.7 mm long; calyx 4.8–7.7 mm long, its teeth 2.3–4.7 mm long***D. saffordi***42A.Leaflets 7–13 pairs per leaf, dark-green; bracts persistent; flowers in conic or pyramidal dense racemes 1.2–1.5 cm wide without petals; petals turning pink-brown or black when dry***D. botterii*** var. ***botterii***42B.Leaflets 2–6 pairs per leaf, light-green; bracts late deciduous; flowers in narrow oblong, conic, conic-ovoid, subglobose or short oblong racemes, 0.8–1.2 cm wide without petals; petals without changing color when dried4343A.Flowers in dense conic, conic-ovoid, subglobose, or short oblong racemes, 0.7–0.9 mm wide without petals; leaflets 2–4 pairs per leaf***D. dorycnoides***43B.Flowers in narrow spiked racemes, 0.8–1.2 cm wide without petals; leaflets 3–6 pairs per leaf4444A.Leaflets sparsely pilose, glabrescent, green or greenish, spikes lax***D. bicolor*** var. ***bicolor***44B.Leaflets densely satiny-pubescent on both surfaces, spikes relatively compact***D. bicolor*** var. ***argyraea***

***Dalea aurea*** Nutt. ex Pursh, Fl. Amer. Sept. 740. 18. Basionym: *Parosela aurea* (Nutt.) Poir. in Lamk., Encycl. Suppl. 4: 590. 1816. *Parosela aurea* (Nutt.) Britt., Mem. Torr. Club 5: 196. 1894. *Cylipogon capitatum* Raf., Jour. Phys. 89: 97. 1819. *Petalostemon capitatus* (Raf.) D C, Prod. 2: 244. 1825.

**Type:** United States, [In upper Louisiana (Territory)]. *J. Bradbury*, *n.n.* (Isotype: NY00006819!) Holotype not seen.

**Distinguishing features:** Herbaceous, perennial. Stems commonly erect. Leaves mainly 5-foliolate, rarely 3 to 7-foliolate. Bracts 2.5–5.5 mm long. Flowers light-yellow. Calyx commonly 6.5 mm long or shorter, its teeth 2.5 times longer than the tube. Banner 6.2–8.6 mm long.

**Representative examined material:** Coahuila: 4-VII-1936, *F.L. Wynd*, *C.H. Muller 471* (UAT); 01-IX-2007 *J.A. Encina 2074A* (ANSM); 13-IX-2010 *J.A. Encina 3115* (ANSM); 22-VIII-2007 *M.A. Carranza Pérez y I. Ramírez 4760* (ANSM); 04-IX-2017 *J.A. Encina 5996* (ANSM); 15-VI-2017 *J.A. Encina 5917* (ANSM); 18-IV-2017 *J.A. Encina 5756* (ANSM); 25-V-2016 *J.A. Encina 5403* (ANSM); 30-IX-2014 *R. Trejo 33* (ANSM); 05-X-2007 *S.G. Gómez 317* (ANSM); 30-VIII-1997 A. *García y A. Herera 2712A* (ANSM); 24-IX-1999 *A. García 3788* (ANSM); 12-X-1991 *M.A. Carranza C-927* (ANSM); 15-IX-1992 *J.A. Villarreal 7016* (ANSM); 08-IX-1990 *R. Vázquez 100* (ANSM); 14-VI-1987 *D. Castillo Quiroz 502* (ANSM); 28-VI-1936 *F. Lyle y Cornelius H. Mueller 248* (ANSM); non *date J.A. Villarreal 7094* (ANSM); 21-VI-2007 *J.A. Encina 2485* (ANSM); 11-VI-2007 *J.A. Encina 2398* (ANSM); 02-XI-1988 *J.A. Villarreal y M.A. Carranza 4772* (ANSM); non date *J. Valdés 2252* (ASNM); 11-IX-1991 *M.A Carranza y L. García 1203* (ANSM).

**Distribution:** Recorded only in central and northern Coahuila. Outside the area, distributed from Dakota to Texas, Arizona, and New Mexico (USA), and to Chihuahua, Mexico. This species is distributed in open valleys with desert scrub, as well as in oak and oak-pine forests, 1000–1700 m

***D. bicolor*** Humb. & Bonpl. ex Willd. var. ***argyrea*** (A. Gray) Barneby, New York. Bot. Gard. 27: 426. 1977. Basionym: *Dalea argyraea* Gray, Pl. Wright. 1: 47. 1852. *Parosela paysonie* L.O. Williams, Bull. Torr. Club 61: 252. 1934.

**Type:** USA, Western Texas to El Paso, New Mexico [Devils River], 30-VII-1849, *C. Wright 131* (Isotype: BM001042556!). Holotype not seen.

**Distinguishing features:** Shrubs. Leaflets 3–6 pairs per leaf, densely satiny-pubescent on both surfaces. Bracts late deciduous. Flowers bicolored, banner white or creamy in early stages, soon turning reddish, the wings and the keel pink, violet, purple to blue cobalt, inserted well below middle of the staminal column, in narrow oblong, conic, ovoid, or short compact racemes, the calyces closely contiguous to each other.

**Representative examined material:** Coahuila: 15-IX-1992 *J.A. Villarreal 7014* (ANSM); 23-VIII-2014 *J.A. Encina 4001* (ANSM); 27-IX-2001 *J.A. Encina 907* (ANSM); 10-XII-1997 *G. Herper 97GC086* (ASNM); 19-X-1993 *M.A. Carranza 1887* (ANSM); 17-IX-1989, *E. Estrada 1800* (CFNL). Nuevo León: 15-XI-1991, *E. Estrada 2203* (CFNL); 11-XI-2001, *C. Yen y E. Estrada 13245* (CFNL); 20-XI-1990, *E. Estrada 1927*; 9-X-1991, *E. Estrada 2118* (CFNL); 24-X-1995 *G. Hinton 25687* (ANSM).

**Distribution:** The var. *argyrea* is differentiated from the var. *bicolor* by its density (satiny to satiny), pubescence, and compact inflorescences with the calyces closely contiguous which frequently completely cover the axis of the raceme, outside the area, in southern USA (Texas and New Mexico). Calciphile, but also found in other types of soils, 400–1770 m, inhabiting desert scrublands, oak forest, and conifer forest.

***D. bicolor*** Humb. & Bonpl. ex Willd. var. ***bicolor*.** Basionym: *Dalea bicolor* Humb. & Bonpl. ex Willd., Hort. Berol. 89. 1809. *Dalea tuberculata* Lag., Gen. & Sp. Nov. 23. 1816. *Parosela tuberculata* (Lag.) Rose, Contrib. U. S. Nat. Herb. 10: 104. 1906. *Dalea thymoides* Schltdl. & Cham., Linnaea 5: 580. 1830. *Dalea laevigata* G. Don, Gen. Hist. Diehl. PI. 2: 224. 1832. *Dalea verrucosa* (warty) G. Don, Gen. Hist. Diehl. PI. 2: 225. 1832. *Dalea comosa* Schltdl., Linnaea 12: 289. 1838. *Dalea ehrenbergii* Schltdl. in op. cit. 290. 1838. *Dalea seemanni* S. Watson, Proc. Amer. Acad. 22: 470. 1887. *Parosela seemanni* (S. Watson) Rose, Contrib. U. S. Nat. Herb. 10: 106. 1906. *Parosela longeracemosa* T. S. Brandegee, Univ. Calif. Pub. Bot. 10: 184. 1922.

**Type**: Mexico, *Seemann 2188* (Holotype: K000081956!).

**Distinguishing features:** Very similar to var. *argyraea*, except for its pubescence on the leaves and more compact racemes (see distribution).

**Representative examined material:** Coahuila: 18-X-1989, *M.A. Carranza*, *511*, *P. Paterson* ((AT). Nuevo León: 9-XI-2002, *C. Yen y E. Estrada 151931* (CFNL); 31-VII-1990, *Hinton* et al. *20623* (MEXU, TEX-LL); 2-VII-1934, *M.T. Mueller 938* (TEX-LL); 28-X-1982, *J. Grimes 2360* (MEXU, TEX-LL). Tamaulipas: 29-IX-1959, *M.C. Johnston 4145* (UAT).

**Distribution:** Var. *bicolor* is commonly differentiated from var. *argyrea* by its loosely pilose pubescence or glabrescent leaves, and by the frequently loose and open spikes with the calyces not contiguous to each other, where they do not completely cover the axis of the raceme. Outside the area, in southern USA (Texas and New Mexico). Frequent in northeastern Mexico, in desert scrublands (Tamaulipan thornscrub and piedmont), also in *Larrea tridentata* scrublands of the high plains, reaching oak and conifer forest, 360–1680 m.

***D. boraginea*** Barneby, New York Bot Gard. 27: 560. 1977.

**Type:** Mexico, [state of Coahuila] Melchor Muzquiz: Palm Canyon, 9-VII-1936, E.G. *Marsh 379* (Holotype: GH00053633!. Isotype: MEXU00056560!).

**Distinguishing features:** Herbaceous perennial. Stems erect, gray or silvery pilose, trichomes ascending. Leaves almost always 3-foliolate, rarely 5-foliolate. Calyx teeth 2.5 times longer than the tube. Petals yellow. Calyx intercostal spaces with only one elongated gland.

**Representative examined material:** Coahuila: 5-XII-1985, *R. Salgado Zavala n.n.* (ASU0020035!); 9-VII-1936, E.G. *Marsh 1937* (GH 00053633!); 24-VIII-1975, *T.L. Wendt*, *E. J. Lott*, *D.H. Riskind 1308* (TEX).

**Distribution:** Endemic to northeastern Mexico, at Sierra de Santa Rosa Coahuila), restricted to oak and oak-pine forest environments., 2000–3000 m.

***D. botterii*** (Rydb.) Barneby var. ***botterii***. Basionym: *Parosela botterii* Rydb., N. Amer. FI. 24: 110. 1920. 

**Type:** Mexico, Veracruz, Orizaba, *M. Botteri 973* (GH00064241!).

**Distinguishing features:** Shrub, stems erect, up to 1.5 m tall, glandular tuberculate, young branches purplish. Leaflets 7–13 pairs per leaf, dark-green; bracts persistent; flowers in conic or pyramidal dense racemes. Flowers concolorous, blue, violet, or purple, turning pink-brown or black when dry.

**Representative examined material:** Nuevo León: 25-X-2003, *E. Estrada 15848* (CFNL); 25-X-2003, *E. Estrada 15833* (CFNL); 8-VI-2003, *C. Yen y E. Estrada 15737* (CFNL); 8-VI-2003, *C. Yen y E. Estrada 15731* (CFNL); 26-VIII-1989, *J.A. Villarreal 5095* (ANSM, TEX-LL); 11-XI-2001, *C. Yen y E. Estrada 13241* (CFNL); 15-XI-1991, *E. Estrada 2202* (ANSM); 03-VIII-1993, *G. B. Hinton 23299* (ANSM); 13-XI-1993, *G. B. Hinton 23941* (ANSM); 18-XI-1993, *J. A. Villarreal 7677* (ANSM). Tamaulipas: 5-VIII-1941, *L.R. Stanford 710* (NY01278431!).

**Distribution:** Endemic of Mexico. In the mountains and high plains of northeastern Mexico. Outside the area, in Veracruz and Puebla. Var. *atrocyanea* of this species is found in Oaxaca and Puebla (Barneby, 1977). Distributed in open oak and oak-pine forest, 1600–2900 m.

***D. brachystachya*** A. Gray, PI. Wright. 2: 39. 1853. Basionym: *Parosela brachystachya* (A. Gray) A. Hell, Catal. N. Amer. PI. ed. 2, 113. 1900. *Dalea lemmoni* A. Gray, Proc. Amer. Acad. 17: 200. 1882.

**Type:** USA, New Mexico [Valley of Sonora] along branches, 13-IX-1951, *C. Wright 990* (Holotype: GH00053587!).

**Distinguishing features:** Herbaceous, diffuse, commonly annual or short perennial. Stems glabrous. Petals yellow, turning purple-brown or pink-brown soon: leaflets 1–5 pairs per leaf; racemes up to 1.6 cm long.

**Representative examined material:** Coahuila: 11-X-2008, *J. A. Alba 321* (ANSM); 14-VIII-1983, *A. Rodríguez y M. A. Carranza 1099* (ANSM); Nuevo León: 1-VIII-1990, *Hinton* et al. *20611* (TEX-LL); 21-VI-2003, *C. Yen y E. Estrada 15782* (CFNL, MEXU); 24-X-1982, *J. Grimes* et al. *2302* (TEX); 22-VI-2003, *C. Yen y E. Estrada 15763* (CFNL); 11-X-2008; *E. Estrada 20602* (ANSM).

**Distribution:** From southwestern USA, throughout the north of Mexico to Hidalgo and Puebla. Especially in desert scrublands, abundant in *Larrea* thickets, frequent along the road, in areas with disturbance and secondary vegetation, desert scrublands. 

***D. capitata*** S. Watson, Proc. Amer. Acad. 25: 146. 1890 var. ***capitata***. Basionym: *Parosela capitata* (S. Watson) Rose, Contr. U.S. Nat. Herb. 12: 272. 1909.

**Type:** Mexico [Coahuila], Carneros Pass, 5-IX-1889, *C.G. Pringle 2378* (Holotype: GH00053634!. Isotype: NY00006888!; MEXU01169320!).

**Distinguishing features:** Shrub up to 50 cm tall. Stems glabrous, young parts with verrucose glands. Leaflets 2–6 pairs per leaf. Flowers in terminal, elongated spikes. Calyx teeth, subulate, pilose or glabrous, the dorsal one the longest 1–3 mm long, all lobes splitting at the same height. Petals light yellow, turning brown, brown-orange to rubescent.

**Representative examined material:** Coahuila: 23-III-1992, *J.L. Neff 92-323-8* (MEXU); 08-VIII-1976, *J. Henrickson y B. Prigge 15102* (MEXU); 15-IV-2015, *J. A. Encina y J. M. Cárdenas-Villanueva 4437* (ANSM); 29-IV-1988, *J. A. Villarreal 4248* (ANSM); 9-V-1987, *J. A. Villarreal 2319* (ANSM). Nuevo León: 10-V-2003, *C. Yen y E. Estrada 15589* (CFNL); 29-VIII-1989, *E. Estrada C. 1737* (CFNL, MEXU, TEX-LL); 22-III-2003, *C. Yen y E. Estrada 15331* (CFNL); *Hinton* et al. *19705* (TEX-LL); 07-IV-2007, *E. Estrada 20106* (ANSM, CFNL); 30-VIII-2008, *G.B. Hinton 28696* (ANSM). Tamaulipas: 08-X-1982, *J. Hernrickson y W. Hess 19147* (ANSM).

**Distribution:** Endemic to Mexico, from Durango, San Luis Potoí, and Zacatecas to northeastern Mexico. In high plains with arid scrub, *Pinus cembroides* forests and dwarf oaks, on calcareous soils, 1600–2600 m.

***D. capitata*** S. Watson var. ***lupinocalyx*** Barneby, New York Bot. Gard. 27: 447. 1977.

**Type:** Mexico, Nuevo León, Sierra Madre Oriental: E of Puerto Los Encinos, Galeana District. Alt. 6050 ft., 29-X-1964, *H.D.D. Ripley*, *R.C. Barneby 13573* (Holotype: NY00006890!).

**Distinguishing features:** Very similar in growth form to var. *capitata*, although it is distinguished by its gland-dotted leaflets on both surfaces; the ventral pair of calyx teeth are united, forming a bidentate lip with the cleft between them less deep than that of the lateral teeth, the calyx silky pilose and its tube only 3–4 mm long.

**Representative examined material:** Nuevo León: 2-III-2003, *C. Yen y E. Estrada 15227* (CFNL); 26-XI-1961, *H.D. Ripley y R.C. Barneby 14778* (MEXU); 22-III-2003, *C. Yen y E. Estrada 15303* (CFNL); 22-III-2003, *C. Yen y E. Estrada 15339* (CFNL); 9-X-1959, *M.C. Johnston 4230* (TEX-LL); 17-XI-2007, *G. Hinton 28114* (ANSM).

**Distribution:** Narrow endemic to the state of Nuevo León, in white calcareous soils, in low hills of intermountain valleys between municipalities of Galeana and Iturbide, 1500–1800 m. This variety is the dominant element in some areas, in intermontane valleys with open desert scrublands, composed almost exclusively of this species. Local people call it “*engorda cabras*” (fatten goats).

***D. capitata*** S. Watson var. ***pseudohopses*** Barneby, New York Bot. Gard. 27: 447. 1977.

**Type:** Mexico, Tamaulipas, 10-VII-1949, *Stanford*, *Lauber*, *Taylor 2388* (Holotype: US, not seen).

**Distinguishing features:** Similar in growth and size to the two previous varieties, although the ventral pair of calyx teeth united and formed a bidentate lip with the cleft between them less deep than that of the lateral lobes, but its calyx is externally glabrous, with only the tube orifice silky-villose.

**Representative examined material**: Border of Nuevo León and Tamaulipas states: 3-VIII-1981, *G. Nesom 4272* (MEXU, TEX-LL). Nuevo León: 22-IV-1984, *C. P. Cowan 4629* (ANSM); 20-X-1978, *Hinton 17444* (ANSM, MEXU). 26-V-1983, *Hinton 18433* (MEXU). Tamaulipas: 19-XI-1991, *E. Estrada 2289* (CFNL, NY).

**Distribution:** Endemic to central and southern Nuevo León, on the slopes of the Cerro El Potosí in the municipalities of Galeana and Dr. Arroyo, and on the slopes of the Cerro Peña Nevada, municipality of Miquihuana at southwestern Tamaulipas. In calcareous soils. Restricted to the high mountain slopes of northeastern Mexico, in desert scrublads and pinyon pine (*Pinus cembroides*), 1750–2000 m.

***D. cliffortiana*** Willd., Willd., Sp. PI. 3: 1336. 1802. Basionym: *Psoralea dalea* L., Sp. PI. 764. 1753. *Psoralea annua* Houst. ex Mill. Gard. Dict., ed. 8.: 6. 1768. *Dalea linnaei* Michx., Fl. Bor. Amer. 2: 57. 1803. *Dalea acutifolia* (Moc. & Sessé ex DC.) Rose, Bot. Gaz. 40: 144. 1905. *Parosela acutifolia* (DC.) Rose, Bot. Gaz. 40: 144. 1905. *Dalea annua* Kuntze Revis. Gen. Pl. 1: 178. 1891. *Dalea annua* var. *willdenowii* Kuntze, Revis. Gen. Pl. 1: 178. 1891. *Dalea dalea* (L.) MacMill., Metasp. Minn. Vall. 330. 1892. *Parosela dalea* (L.) Britton, Mem. Torr. Bot. Club 5: 196. 1894. *Parosela cliffordiana* (Willd.) Rose, Contr. U.S. Natl. Herb. 10: 105. 1906. *Thornbera dalea* (L.) Rydb., N. Amer. Fl. 24: 120. 1920. *Dalea acutifolia* Moc. & Sessé ex DC., Prodr. 2: 245. 1825. *Dalea angustifolia* G. Don, Gen. Hist. Diehl. PI. 2: 223. 1832. *Amorpha glandulosa* Blanco, Fl. Filip. 555. 1837. *Dalea glandulosa* (Blanco) Merrill, Philip. Gov. Bur. Lab. Bull. 27: 37. 1905. *Dalea nigra* M. Martens & Galeotti, Bull. Acad. Roy. Sci. Brux. 10(2): 43. 1843. *Parosela nigra* (M. Martens & Galeotti) Rose, Contrib. U.S. Nat. Herb. 10: 105. 1906. *Dalea virgata* Michelli, Bull. Herb. Boiss. 2: 442. 1894. *Parosela dalea* var. *robusta* (Rydb.) J.F. Macbr. Contr. Gray Herb. 65: 22. 1922. *Dalea robusta* Rydb. N.L. Britton & al. (eds.), N. Amer. Fl. 24: 121. 1920. *Thornbera pumila* Rydb. N.L. Britton & al. (eds.), N. Amer. Fl. 24: 120. 1920. *Dalea pumila* (Rydb.) L. Riley, Bull. Misc. Inform. Kew 1923: 337. 1923.

**Type**: Lectotype: Linnaeus, Hort. Clifford. t. 22 (1738), designated by R.C. Barneby, Mem. New York Bot. Gard. 27: 193. 1977.

**Distinguishing features:** Herbaceous. Stems and leaves glabrous; leaflets acute, linear-oblanceolate or elliptic; calyx sessile; petals bicolored, the banner white, turning rubescent with age, the wings and keel blue, violet, pink, or purple, rarely whit; keel-petals with their margins overlapping, one over the other, never fused at their outer margins, 2.1–3.4 mm long; stamens immersed in the keel or exserted.

**Representative examined material**: Tamaulipas: 10-XII-1959, *M.C. Johnston*, *C. McMillan 4948E* (TEX00247566).

**Distribution:** Widespread in Mexico, most common in the northwestern and southern parts of the country, 10–1800 m, also from Central America to Venezuela and Ecuador. Rare in northeastern Mexico, only recorded in southern Tamaulipas (municipality of Aldama). A distinctive characteristic of this species and other related taxa is the presence of a keel, where its petals are not united at their edges (valvate) and constituting a conventional keel, but instead, their petals are imbricated (one on top of the other at their edges) in its initial stages of development; however, when the flower matures, the keel petals separate. In low plains, rare in northeastern Mexico, in desert scrublands, 150–250 m.

***D. dorycnioides*** DC., Prod. 2: 245. 1825. Basionym: *Parosela dorycnioides* (DC.) Rydb., N.L. Britton & al. (eds.), N. Amer. Fl. 24: 90. 1920. *Dalea pulchella* Moric, PI. Nouv. Amer. 9, Tab. VII. 1836. *Parosela pulchella* (Moric.) A. Heller, Cat. N. Amer. Pl, Ed 2: 6. 1900. *Dalea decora* S. Schauer, Linnaea 20: 743. 1847. *Parosela decora* (S. Schauer) Rydb., N.L. Britton & al. (eds.), N. Amer. Fl. 24: 90 (1920).

**Type:** Mexico, St. Louis de Potosi [in fact Tamaulipas], de Victoria a Tula, XI-1830, *Berlandier 2202* (Isolectotype: MO-126308!; BM000931511!). Holotype not seen.

**Distinguishing features:** Shrubby, perennial, with multiple stems, up to 1.1 m tall, young parts verrucose, velvety to silky pilose; petals bicolored, the banner opening white or yellow, but reddish with age, wings and keel petals white, pink, purple, magenta, or violet, never yellow; leaflets 2–4 pairs per leaf, silvery to gray pubescent on both surfaces, sometimes glabrous adaxially; inflorescences narrow oblong, conic, conic-ovoid, subglobose or short oblong racemes; petals without changing color when dried.

**Representative examined material**: Coahuila: 11-X-2008, *E. Estrada 20697* (CFNL, TEX-LL); 11-X-2008, *J. A. Alba 358* (ANSM); 11-X-2008, *J. A. Alba 336* (ANSM). Nuevo León: 23-IV-2023, *E. Estrada 26177* (CFNL); 20-X-1984, *J. Saunders-Scherer*, *K.C. Nixon 1343a* (TEX-LL); 13-XI-1964, *H.D.D. Ripley 13793* (NY01278732); 25-x-1981, *J.M. Poole*, *K.C. Nixon*, *D. Smith 2466* (TEX-LL). Tamaulipas: 6-X-1982, *J. Henrickson 19088* (NY01278774); 13-VIII-1941, *L.R. Stanford 880a* (NY01278768); 25-X-1942, 15-VIII-1972, *F. González-Medrano 4685* (UAT, ARIZ352801).

**Distribution**: Endemic to Mexico, from Chihuahua and Durango to Tamaulipas, as well as San Luis Potosí to Hidalgo and Oaxaca. Morphologically similar to *Dalea bicolor*; however, it is easy to distinguish them, because *D. dorycnoides* has hemispherical or ovoid inflorescences, while *D. bicolor* has elongated spikes commonly 3 cm long or longer. Mainly in calcareous soils, desert scrublands, and oak-pine forests, 1100–2200 m.

***D. emarginata*** (Torr. & A. Gray) Shinners, Field & Lab. 17: 84. 1949. Basionym: *Petalostemon emarginatum* Torr. & A. Gray, Fl. N. Am. 1: 311. 1838. *Kuhnistera emarginata* (Torr. & A. Gray) Kuntze, Revis. Gen. Pl. 192. 1891.

**Type:** USA, Rio Brazos, 1833, *T. Drummond n.n.* (Syntype: GH00274749!, L0018913!).

**Distinguishing features:** Herbaceous, annual, or biennial. Stems several suberect or incurved, up to 60 cm tall, monocephalous. Leaflets 4–8 pairs per leaf. Inflorescences in terminal spikes. Peduncles 6–40 cm long, far surpassing the leaves. Flowers purple magenta to violet. Stamens commonly 5, free filaments 1/3 or less of the length of the staminal column.

**Representative examined material**: Coahuila: 15-IV-1999, *A. Mora-Olivo 7575* (ANSM, UAT). Nuevo León, 15-X-2024, *E. Estrada 26636* (CFNL). Tamaulipas: 2-X-1984, *Baro*, *E. Aguilar*, *R. Funetes*, *S. Rodríguez 462* (UAT); 13-II-1985, *D. Baro*, *C. Aguilar*, *R. Fuentes 629* (UAT); 4-VII-1941, *R.L. Croquet 1076* (LL00247619);

**Distribution**: From southeastern USA, Louisiana, and Texas to Tamaulipas and Veracruz. A very peculiar and easily recognizable species by its commonly long terminal peduncles up to 40 cm long; the wings, and the keel petals inserted at or almost so at the apex of the staminal column, and by having only 5 stamens, inhabiting sandy soils, dunes, and sea beaches, 0–250 m, and also in calcareous soils with desert scrublands, and oak-pine forest, 1300 m.

***D. eriophylla*** S. Watson, Proc. Amer. Acad. Sci. 17: 340. 1882. Basionym: *Parosela eriophylla* (S. Watson) Rose, Contrib. U.S. Nat. Herb. 10: 106. 1906.

**Type**: Mexico, Sierra Madre, 40 miles south of Saltillo; vicinity of San Antonio de Alanzanes [Alazanas], Mpio Arteaga, Coahuila, VII-1880, *E. Palmer 211* (Holotype: GH00053642!. Isotype: NY00006901!).

**Distinguishing features:** Perennial, dwarf shrub, up to 30 cm tall. Stems white pubescent. Leaves digitate (palmately)-trifoliolate. Inflorescences in terminal, sessile short spikes and almost covered by the apical leaves. Leaflets obovate to oblanceolate, flattened, or slightly revolute. Petals bicolored, the banner opening white or yellow, but rubescent with age, wings, and keel petals pink, purple, or violet, never yellow.

**Representative examined material**: Coahuila: 15-IV-2006, *J.A. Encina 3695* (ANSM); 07-V-1983, *A. Rodríguez y M. Carranza 912* (ANSM); 08-X-2010, *J. A. Villarreal 9501* (ANSM). Nuevo León: 11-V-1989, *E. Estrada 1457* (CFNL, TEX-LL); 3-VIII-1981, *G. Nesom 4278* (MEXU, TEX-LL). 2-XI-1971, *Hinton 17313* (MEXU); 13-XI-1964, *H.D. Ripley*, *R.C. Barneby 1379* (MEXU); 26-VIII-1987, *D. Bogler*, *T. Atkins 147* (MEXU, TEX-LL); 05-XII-2003, *G.B. Hinton 28230* (ANSM). Tamaulipas: 25-X-1989, *L. Hernández. M. Martínez 2060* (UAT); 16-VI-1984, *J. V. Reyna y M. A. Carranza 114* (ANSM).

**Distribution**: Endemic to northeastern Mexico, and San Luis Potosí. Easily recognized by its palmate-trifoliolate white pubescent leaves, bicolored flowers, and sessile inflorescences, inhabiting almost always in chalky soils at intermontane valleys and high plains of northeastern Mexico. Morphologically similar to *D. dorycnioides* (this with a higher number of leaflets per leaf, and larger size), *D. bicolor* (higher number of leaflets per leaf, and with much longer inflorescences, 3–16 cm long), and *D. greggii* (this with a higher number of leaflets per leaf, and the apex of its branches rooting). In calcareous and gypsum soils, desert scrublands, arid oak-conifer forest, and conifer forest, 1700–2700 m. Locally abundant.

***D. foliolosa*** var. ***citrina*** (Aiton) Barneby, Phytologia 26: 1. 1973. Basionym: *Parosela citrina* Rydb., N.L. Britton & al. (eds.), N. Amer. Fl. 24: 81. 1920. *Parosela vernicia* var. *citrina* (Rydb.) J.F. Macbr., Contr. Gray Herb., New Ser. 65: 17. 1922. *Dalea citrina* (Rydb.) Bullock, Bull. Misc. Inform. Kew 1939: 195. 1939. *Parosela vernicia* Rose, Contrib. U.S. Nat. Herb. 8: 303. 1905. *Dalea vernicia* (Rose) Greenm., Publ. Field Mus. Nat. Hist. Bot. 2: 331. 1912.

**Type**: Mexico, fields about Tuxpan, state of Jalisco 27-X-1904, *C.G. Pringlei 8860* (Isotype: E00285886!, MO-125120!, HBG519986!, M0233352!).

**Distinguishing features:** Herbaceous, annual, arising solitary from the base and branching above the middle, glabrate. Leaflets 5–17 pairs per leaf. Inflorescences in long racemes 6–30 cm long. Longest calyx tooth 1.4–4.3 mm long. Petals bicolored, the banner opening white in part, but rubescent with age, wings, and keel petals dull pink, reddish-purple, magenta or violet.

**Representative examined material**: Nuevo León: 27-IX-1998, *M.A. Carranza*, *J. Valdés R. C-2978* (TEX00247723); 25-IX-1978, *J. Henrickson 17601* (TEX00418469). Tamaulipas: X-1932, *H.W. Von Rozynski 568* (NY01279102). 

**Distribution:** Not very frequent. Outside the area distributed to southern Mexico, Central America, Venezuela, and Colombia. In high plains and mountains, in desert scrublands and gypsophilous vegetation, 1450–2000 m.

***D. foliolosa*** (Aiton) Barneby, Phytologia 26: 1. 1973. var. ***foliolosa***. Basionym: *Psoralea foliolosa* Aiton, Hort. Kew. 3: 82. 1789. *Psoralea citriodora* Cav., Ic. 3: 36, 1796. *Dalea citriodora* (Cav.) Willd., Sp. PI. 3: 1339. 1801. *Parosela citriodora* (Cav.) Rose, Contrib. U.S. Nat. Herb. 10: 104. 1906. *Dalea polyphylla* M. Martens & Galeotti., Bull. Acad. Bruxelles. 10(2): 44. 1843. *Parosela polyphylla* (M. Martens & Galeotti) Rose, Contr. U.S. Nat. Herb. 10: 104. 1906. *Dalea platystegia* S. Schauer, Linnaea 20: 741. 1847. *Psoralea citrodora* Sesse & Moc., Fl. Nov. Hisp. 120. 1889. *Parosela roseola* Rydb., N.L. Britton & al. (eds.), N. Amer. Fl. 24: 81 1920. *Dalea roseola* (Rydb.) Cowan, Brittonia 8: 60. 1954.

**Type**: Mexico, *Sessé & Mociño 2666bis* (Neotype: MA601685!)

**Distinguishing features:** Very similar to the previous variety, but with the stems diffusely branched from the base, sometimes branched apically, and the stems erect. The longest calyx tooth up to 1.3 mm long. Petals of the wings and keel of pink, purple, light blue, or violet.

**Representative examined material**: Coahuila: 31-I-1964/13-XI-1964, *H.D.D. Ripley 13802* (NY01278949).

**Distribution:** Not very frequent in northeastern Mexico. Outside the area, from Chihuahua and Coahuila to Chiapas, extending its distribution to Guatemala and Honduras. Rare in the study area. Along roads and highways, and in disturbed areas, mainly in desert scrublands, 1500–2450 m.

***D. formosa*** Torr., Ann. Lyc. N.Y. 2: 177. 1827. Basionym: *Parosela formosa* (Torr.) Vail, Trans. N. Y. Acad. Sci. 14: 34. 1894.

Type: USA, [on the Platte], *E. James n.n.* (Holotype: NY00006828!).

**Distinguishing features:** Shrub 50 cm or taller. Leaves 2.5–11 mm long. Leaflets 3–7 pairs, pubescent. Flowers 2–9 in short subcapitate racemes, raceme axis 9 mm or less. Calyx 7.4–16.2 mm long, its teeth 4.5–8.5 mm long, longer than a tube. Petals bicolored, the banner opening white or yellow, but rubescent with age, wings, and keel petals pink, purple, magenta, or violet.

**Representative examined material**: Coahuila: 29-III-1992, *M. A. Carranza 1527* (ANSM); 10-VIII-1995, *M. A. Carranza C-2356* (ANSM); 27-III-1992, *M. A. Carranza 1342b* (ANSM); *J. Encina 5812* (BUAP72883!).

**Distribution**: Similar to *D. frutescens*, although, the latter has stems with warty or tuberculate glands, a glabrous calyx, and shorter teeth than the tube. South of USA (Texas, New Mexico, and Oklahoma to Northern Mexico (Sonora. Chihuahua, and Coahuila), in desert scrublands, arid conifer forest, mesquite scrubland, and desert grasslands, 540–1980 m, in plains with rocky soils, and disturbed areas.

***D. frutescens*** A. Gray, Boston Jour. Nat. Hist. 6 (PI. Lindh. 2): 175. 1850. Basionym: *Parosela frutescens* (A. Gray) Rose, Contrib. U. S. Nat. Herb. 8: 303. 1905. *Parosela laxa* Rydb., N.L. Britton & al. (eds.), N. Amer. Fl. 24: 85. 1920. *Parosela frutescens* var. *laxa* (Rydb.) B. L. Turner, Field & Lab. 24: 16. 1956.

**Type**: USA, Texas; Dry, rocky prairies, R. Colorado., 31-XII-1846/1-I-1847, *F.J. Lindheimer 376* (Isotype: NY00006838!). Holotype not seen.

**Distinguishing features:** Shrub up to 1.2 m tall. Stems tortuose, decumbent, and rarely rooting at the tips of branches, glabrous, with verrucose or tuberculate glands. Leaflets 4–10 pairs, glabrous. Flowers bicolored, very rarely all petals white, the banner opening whitish with a green-yellow spot, although turning rubescent, the wings and keel pink or purple. Calyx glabrous, its teeth shorter than the tube.

**Representative examined material**: Coahuila: 7-IX-1990, *M.A. Carranza C-688*, *J. Valdés*, *P. Fryxell*, *R. Vázquez* (UAT); 1-X-1993, *M.A. Carranza 1725*, *J. Encinas*, *F. Fierro*, *R. Rodríguez* (UAT); 18-VIII-2001, *J.A. Encina 796* (ANSM); 01-IX-2007, *J.A. Encina 2049* (ANSM); 07-IX-2007, *J.A. Encina 2169* (ANSM); 28-IX-1999, *J.A. Villareal 8954* (ANSM); 27-VII-1979, *D. Arredondo 129* (ANSM); 20-IV-2017, *J.A. Encina 5780* (ANSM); 12-VIII-2004, *D. Riskind*, *J. Valdez y Henrickson 23871* (ANSM); 17-IX-1999, *J.A. Villarreal 8793* (ANSM); 11-XI-1997, *M.A. Carranza 2740* (ANSM). Nuevo León: 24-VI-2001, *C. Yen y E. Estrada 12799* (CFNL); 23-VII-1977, *C. Wells*, *G. Nesom 132* (TEX-LL). 2-IV-1978, *J.G. Moya R. 47* (MEXU); 08-X-1995, *S. Rodríguez 131* (ANSM).

**Distribution**: From southern USA (New Mexico, Texas, and Oklahoma) to northern Mexico (Chihuahua, Coahuila, and Nuevo León to Zacatecas). Morphologically similar to *D. formosa*, although the latter species has stems without warty or tuberculate glands, pubescent calyx, and its teeth are longer than the tube. Frequent in calcareous soils, in low and high plains, in desert scrublands, oak-pine forest, arid conifer forest, 360–1980 m.

***D. greggii*** A. Gray, Mem. Amer. Acad. Arts, n.s. 5: 314. 1855. Basionym: *Parosela greggii* (A. Gray) A. Heller, Cat. N. Amer. PI. ed. 2, 6. 1900. *Parosela fulvosericea* Rydb., N. Amer. Fl. 24: 89. 1920. *Dalea fulvosericea* (Rydb.) Gentry, Madroño 10: 249. 1950.

**Type:** Mexico [Coahuila], Buenavista, from battlefield, 27-III-1847, *J. Gregg 348* (Isotype: MO126306!). Holotype not seen.

**Distinguishing features:** Small, scandent shrub, silvery-villous, arching, its branches rooting at the tips when they meet the ground, forming colonies up to 1 m in diameter. Leaflets 2–4 pairs per leaf. Spikes 0.5–3 cm long, rarely longer, compact, globose, or subglobose to oblong cylindric. Flowers bicolored, the banner opening pale yellow or cream, but soon rubescent, the wings and keel pink or purple, mm long. Stamens 10.

**Representative examined material**: Coahuila: 26-III-1981, *M. A. Carranza 1038* (ANSM); 20-VIII-1987, *J. A. Villarreal 3908* (ANSM); III-1985, *J. A. Villarreal 2886* (ANSM); 12-X-1991, *M. A. Carranza C852* (ANSM); 11-X-1991, *M. A. Carranza C981* (ANSM); 8-IX-1990, *R. Vásquez 205* (ANSM); 07-II-2015, *J. A. Encina 4321* (ANSM); 26-VIII-2010, *J. A. Encina 2900* (ANSM); 09-II-1987, *S. González 3942*(ANSM); 27-III-1992, *M. A. Carranza 1342d* (ANSM). Nuevo León: 2-III-2003, *C. Yen y E. Estrada 15265* (CFNL). 13-IV-2003, *C. Yen y E. Estrada 15523* (CFNL); 21-VI-2003, *C. Yen y E. Estrada 15795* (CFNL); 8-VI-2003, *C. Yen y E. Estrada 15752* (CFNL); *15-VI-1989*, *E. Estrada 1504* (CFNL, MEXU); 15-V-1986, *E. Estrada 439* (MEXU); 20-III-1983, *G. B. Hinton 18382* (ANSM); 11-X-2003, *G. B. Hinton 27905* (ANSM); 09-XI-1993, *G. B. Hinton 23845* (ANSM). Tamaulipas: 28-V-1986, *L. Hernández 1807* (UAT); 7-XII-1976, *F. Guevara 10132* (UAT); 7-XII-1993, *A. Mora-Olivo 5036* (UAT); 29-X-1982, *F. Uribe 137* (BCMEX); 19-VIII-1994, *L. Hernández 3161* (UAT); 31-VIII-1994, *L. Hernández 3305* (UAT).

**Distribution**: From southeastern USA (Texas) to Chihuahua, and extending to Hidalgo, Querétaro, Puebla, and Oaxaca. Distinctive species for its habit of forming colonies, with decumbent stems and the apices of its branches rooting when they touch the ground. In clay, silty, chalky, and calcareous soils, in desert low plains, high plains, and mountains. Desert scrublands, mezquital (*Neltuma* spp.), oak, and oak-pine forest, 0–2250 m.

***D. gypsophila*** Barneby, Mem. New York Bot. Gard. 27: 442–443. 1977.

**Type:** Mexico, 2.5 miles S of Galeana, mun. Galeana, Nuevo León, 29-X-1964, *H.D. Ripley & R.C. Barneby 13577* Holotype not seen. (Isotype: ENCB003352!; CAS0002168!).

**Distinguishing features:** Dwarf shrubs up to 25 cm high, rooting adventitiously in the basal or apical parts of the stems, forming small patches of 0.3–1 m diameter. Leaflets with tiny white papillae; calyx silky-pubescent; petals without tiny scattered glands. Flowers bicolored, the banner opening cream or light-yellow, commonly purple-edged, but soon rubescent, the wings and keel dark plum to purple,

**Representative examined material**: Nuevo León: 12-IV-2003, *C. Yen y E. Estrada 15486* (CFNL); *C. Yen y E. Estrada 15798* (CFNL); 15-V-2003, *C. Yen y E. Estrada 15599* (CFNL); 8-X-1993, *M.A. Carranza 1611* (TEX-LL); *H.D. Ripley y R.C. Barneby 13792* (MEXU); 17-V-1973, *M.C. Johnston*, *T.C. Wendt*, *F. Chiang 110591* (TEX-LL); 5-VIII-1993, *M. Cotera y E. Estrada 2453* (CFNL); 17-VI-1992, *J. A. Villarreal 6879* (ANSM); 12-V-1991, *M. A. Carranza 1473* (ANSM); 12-IV-1981, *G. B. Hinton 18231* (ANSM); 01-VI-1989, *E. Estrada 1491* (ANSM). Tamaulipas: 7-XII-1993, *A. Mora-Olivo 5037* (UAT).

**Distribution***:* Endemic to northeastern Mexico, restricted to gypsum soils with shalky grasslands. *D. greggii* is a similar species, although herbaceous, whose stems root at the tips of the stems, although it can be differentiated from *D. gypsophila* by its silvery-villous pubescence and absence of tiny white papillae on the leaflets. Although the stem tips of *D. frutescens* rarely root in the ground, it can be differentiated from *D. gypsophila* by its bushy habit, more than 50 cm tall, glabrous calyx, and the absence of white papillae on the leaflets. Desert scrublands, and arid conifer forest, associated with communities of Mexican prairie dog (*Cynomys mexicanus*), 1800–2100 m.

***D. hospes*** (Rose) Bullock, Kew Bull 1939: 196. 1939, Bull. 1939. Basionym: *Parosela hospes* Rose, Contrib. U. S. Nat. Herb. 12: 272. 1909.

**Type:** Mexico, Grassy slopes of the Sierra Madre near Monterey, State of Nuevo Leon, 28-VI-1888, *C.G. Pringlei 1904* (Isotype: P02773692!; BM000931525!). Holotype not seen.

**Distinguishing features:** Shrub up to 2.7 m. Leaflets 2–5 pairs per leaf, glabrate. Inflorescences in lax recemes. Calyx teeth shorter than the tube, triangular, the ventral pair wider than long, glabrate, or tiny pilose; keel and wing petals inserted near middle of the staminal tube, 3.4–5.4 mm from the base; wing petals 6–7.5 mm long.

**Representative examined material**: Coahuila: 29-VII-1998, *M. A. Carranza 2996* (ANSM); 26-VII-1977, *T. Wendt VR-1023* (ANSM); 08-IX-1990, *R. Vásquez 208* (ANSM); 20-VIII-1987, *J. A. Villarreal 3957* (ANSM); 05-X-1991, *J. A. Villarreal 6170* (ANSM); 31-X-1987, *J. A. Villarreal 4170* (ANSM). 19-IX-1989, *J.A. Villarreal 5216* (UAT). Nuevo León: 6-VII-2001, *J. Luna*, *M. González*, *C. Yen y E. Estrada 12953* (CFNL); 12-IV-2003, *C. Yen y E. Estrada 15516* (CFNL); 23-VII-2002, *C. Yen y E. Estrada 15101* (CFNL); 10-V-2003, *C. Yen y E. Estrada 15578* (CFNL); 21-IV-2000, *C. Yen y E. Estrada 11392* (CFNL); 30-VII-2002, *C. Yen y E. Estrada 15540* (CFNL). Tamaulipas: 27-II-1986, *l. Hernández 1664* (UAT); 15-V-1986, *M. Martínez 1055* (UAT); 15-X-1984, *McDonald 1065* (UAT); 15-VIII-1972, *F. González-Medrano 4681* (ARIZ352793).

**Distribution**: Endemic to northeastern Mexico. *Dalea hospes* is morphologically similar to the three varieties of *D. melantha* found in northeastern Mexico, although the leaves of *D. m.* var. *pubens* are softly puberulent (glabrate in *D. hospes*); in *D. m.* var. *berlandeiri* and *D. m.* var. *melantha* all the calyx teeth extend beyond the length of the tube, are triangular-subulate or aristiform and always plumose; the keel petals and wings are inserted below the middle of the staminal column, 0.8–1.7 mm from the base, and the keel petals are 4.8–5.2 mm long. Species easily recognized by its bushy habit but with thin and relatively thin glabrous stems and branches; flowers yellow or yellowish green, with the calyx glabrous externally, but ciliolate in the apical part of the tube hole. In different types of soils, desert scrubland (piedmont scrub), oak, oak-pine, and conifer forest, 400–2700 m.

***D. lachnostachys*** A. Gray, PI. Wright. 1: 46. 1852. Basionym: *Parosela lachnostachya* (Gray) A. Heller, Cat. N. Amer. PI. ed. 2, 6. 1900.

**Type:** USA, Western Texas to El Paso, New Mexico [hills about 80 miles beyond the Pecos], 21-VIII-1849, C. Wright 125 (Holotype: GH00053600!).

**Distinguishing features:** Herbaceous perennial. Stems with abundant verrucose glands, pubescent. Leaflets 2–5 pairs per leaf. Flowers in dense racemes, separated from each other less than 1 time the length of the calyx; glands when present, scattered. Hairs of the calyx 2.7–4.5 mm long. Petals blue, turning violet when dry. Keel-petals with their margins overlapping, one over the other, not fused at their outer margins. Filaments almost immersed in the keel.

**Representative examined material**: Coahuila: 10-VII-1941, *I.M. Johnston*, *C.H. Muller 1019* (LL00247952); 5-VIII-1976, *B. Prigge 14923-6* (TEX00046424); 5-VIII-1976, *J.S. Henrickson*, *Barry Prigge 14923* (MEXU).

**Distribution:** Southern USA (Arizona, New Mexico, and Texas) and northern Mexico (Sonora to Coahuila). Plains and foothills, desert scrublands and associations of scrub-grassland, 1000–1700 m.

***D. lanata*** Spreng. var. ***terminalis*** (M. E. Jones) Barneby, New York Bot. Gard. 27: 282. 1977. Basionym: *D. terminalis* Jones, Contrib. West. Bot. 12: 8. 1908. *Parosela terminalis* (Jones) A. Heller, Muhlenbergia 6: 96. 1910. *Dalea glaberrima* S. Watson, Proc. Amer. Acad. 22: 470. 1887. *Parosela glaberrima* (Wats.) Rose, Contrib. U. S. Nat. Herb. 10: 103. 1906. *Dalea arenaria* Jones, Contrib. West. Bot. 12: 8. 1908. *Parosela subvillosa* Rydb. N.L. Britton & al. (eds.), N. Amer. Fl. 24: 93.1920. *Dalea subvillosa* (Rydb.) B. L Turner, Field & Lab. 18: 46. 1950.

**Type:** Mexico, Chihuahua. State of Chihuahua. Near Paso del Norte [=Ciudad Juárez], 23-IX-1886, *C.G. Pringle 720* (Holotype: GH00053647!).

**Distinguishing features:** Herbaceous, perennial. Leaves 1–3 cm long. Leaflets 4–7 pairs per leaf, silky pilose, gland-dotted or gland-verrucose abaxially, each leaflet with a prominent, subapical gland. Flowers violet-reddish or purple to magenta, concolorous, with all petals free. Filaments exerted from petals.

**Representative examined material**: Coahuila: 02-IX-2006, *M. González 3936* (ANSM); 20-IX-1974, *J. Henrickson 14159* (ARIZ406402).

**Distribution:** From south USA (Colorado, Arizona, New Mexico, and Texas) to north of Mexico (Chihuahua and Coahuila). This is the only *Dalea* species in northeastern Mexico with 7–10 stamens, free petals, and filaments exerted from the petals. Sand dunes, in desert scrublands.

***D. laniceps*** Barneby, Southwest. Nat. 15(3): 390. 1971.

**Type:** Mexico, Nuevo León: 16 miles SW of Cerralvo, at Arroyo del Fraile, 27-X-1964, *H.D.D Ripley & Barneby 13547* (Holotype: NY00007003!. Isotype: (GH00053657!; CAS0002164!; ENCB003353!; US00003744!).

**Distinguishing features:** Herbaceous, perennial, 5–6 cm tall, aromatic if crushed. Pubescence gray or silvery, ascending, pilose. Stems commonly prostrate. Leaves almost always 3-foliolate, rarely 5-foliolate. Leaflets obovate to elliptic-obovate. Intercostal spaces of the calyx with a row of 3–4 small glands, its teeth 2.5 times longer than the tube. Flowers light-yellow, turning reddish-brown or pink with age. Banner blade up to 2.2 mm long, its claw 2–3 times longer than blade. Keel petals with their margins fused at their outer edges.

**Representative examined material**: Coahuila: 04-VIII-1979, *D. Arredondo 187* (ANSM); 15-IX-2017, *J. A. Encina 6027* (ANSM); 19-IX-1992, *J. A. Vilarreal 7009* (ANSM); 17-IX-1992, *M. A. Carranza C988* (ANSM). Nuevo León: 10-IV-2001, *C. Yen y E. Estrada 12115* (CFNL). 17-IV-2001, *C. Yen y E. Estrada 12440* (CFNL); 21-X-1963, *H.D. Ripley*, *R.C. Barneby 13249* (MEXU); 27-X-1964, H.D. *Ripley*, *R.C. Barneby 13549* (MEXU). 15-V-2003, *C. Yen y E. Estrada 15600* (CFNL). 9-IV-2001, *C. Yen y E. Estrada 12019* (CFNL). 15-IV-2001, *C. Yen y E. Estrada 12213* (CFNL); 3-VII-2024, *E. Estrada 26419* (CFNL).

**Distribution**: Southern Texas (USA) and north of Mexico (Chihuahua, Durango, Coahuila, Nuevo León, Zacatecas, and San Luis Potosí). One of the tiniest *Dalea* in northeastern Mexico. A distinctive feature of this species is the ratio of the size of the banner blade to its claw, the latter being 2–3 times longer. In desert scrublands, 670–2200 m.

***D. lasiathera*** A. Gray, PI. Wright. 1: 48. 1852. Basionym: *Parosela lasiathera* (A. Gray) A. Heller, Cat. N. Amer. PI. ed 2, 6. 1900. *P. lasianthera* Rydb., N. Amer. Fl. 24: 96. 1920.

**Type:** USA, Texas; Prairies, W of San Antonio, Texas, and Valley of the Limpia. V-1849/X-1849, *C. Wright 133* (Isotype: NY00006847!). Holotype not seen.

**Distinguishing features:** Herbaceous perennial. Stems ribbed, not lignified at the base, they disappear after fruiting in the following season. Leaflets 2–5 pairs per leaf, tick, glaucous, light blue-green. Calyx tube 3.3–4 mm long, pilose, the hairs 0.5–1 mm long. Flowers shiny violet, purple to magenta, bicolored, the banner stained yellow in the center and with small glands present, wings and keel petals pink, purple, magenta, or violet.

**Representative examined material**: Coahuila: 1-V-1977, *J. Henrickson 15941* (NY01279458); 4-VII-1936, *F.L. Wynd 488* (NY01279452); 10-VI-1972, *F. Chiang 7602* (NY01279451); 3-IV-1970, *W.F. Mahler 5649* (01279455); 9-VI-1955, *M. Johnston 2558* (MICH 1154657). Nuevo León: 12-X-1989, *E. Estrada 1846* (CFNL, TEX-LL); 4-VI-1987, *E. Estrada 896* (CFNL). 20-VII-1933, *C.H. y M.T. Mueller 496* (MEXU, TEX-LL).

**Distribution**: Endemic to southern Texas and northeastern Mexico. Morphologically similar to *D. pogonathera* (see Distribution for this species), although *D. lasiathera* has a calyx tube that is always longer than 3.3 mm, and longer than its teeth, the pubescence is silky, the trichomes up to 1 mm long, the banner has a yellow spot in the middle, and it has a wider geographical distribution. In Tamaulipan thorn scrub, piedmont scrub and oak-pine forest, also in barren soils, 250–1600 m.

***D. luisana*** S. Watson, Proc. Amer. Acad. 17: 341. Basionym: *Parosela luisana* (S. Watson) Vail, Bull. Torr. Club 24: 16. 1897. *Dalea ternata* T.S. Brandegee, Univ. Calif. Pub. Bot. 3: 380. 190.

**Type:** Mexico, San Luis Potosi, 1879, *J.G. Schaffner 808* (Isotype: NY00007005!). Hlotype not seen.

**Distinguishing features:** Herbaceous, up to 20 cm tall, perennial. Stems and leaves mostly, greenish-gray to canescent, pubescence appressed. Leaves mainly 3-foliolate, rarely 5 foliolate. Leaflets linear-elliptic or linear-oblanceolate. Calyx teeth 2.5 times longer than the tube. Flowers light-yellow turning reddish, orange to brown with age.

**Representative examined material**: Coahuila: 11-X-2008, *J.A. Alba 320* (ANSM); 12--XI-1963, *H.D.D. Ripley 13521* (NY01279683); Nuevo León: 21-VI-2003, *C. Yen y E. Estrada 15793* (CFNL); 23-VII-1999, *E. Estrada 10455* (CFNL). Tamaulipas: 8-VIII-1941, *L.R. Stanford 789* (NY01279689).

**Distribution**: Endemic to Mexico, from Durango to Tamaulipas, and from Zacatecas and San Luis Potosí to Querétaro and Puebla. Morphologically very similar to *D. boraginea*, *D. laniceps*, and *D. prostrata*, but those have elliptical or obovate leaflets and pubescence ascending and pilose. In desert scrublands and arid grasslands, 1400–2600 m.

***D. lutea*** (Cav.) Willd., Sp. PI. 3: 1341. 1801. var. ***lutea****. Basionym: Parosela lutea* Cav., Ic. 4: 12, Pl. 325. 1797. *Dalea ovalifolia* Ort., Dec. 1: 30, tab. 3. 1797. *Dalea leucostoma* Schltdl., Linnaea 12: 294. 1838. *Parosela leucostoma* (Schltdl.) Rose, Contrib. U. S. Nat. Herb. 10: 106. 1906. *Dalea plumosa* S. Watson, Proc. Amer. Acad. 21: 448. 1886. *Parosela plumosa* (S. Watson) Rose, Contrib. U.S. Nat. Herb. 10: 106. 1906. *Parosela painteri* Rose, Contrib. U.S. Nat. Herb. 10: 105. 1906. *Dalea painteri* (Rose) Bullock, Bull. Misc. Inform. Kew 1939: 197. 1939. *Parosela wardii* Rydb., N.L. Britton & al. (eds.), N. Amer. Fl. 24: 112. 1920. *Parosela caudata* Rydb., N.L. Britton & al. (eds.), N. Amer. Fl. 24: 112. 1920. *Parosela lutea* var. *caudata* (Rydb.) Macbr., Contrib. Gray Herb., New Ser. 65: 21. 1922. *Dalea caudata* (Rydb.) Bullock, Bull. Misc. Inform. Kew 1939: 195. 1939.

**Type**: Mexico, In the shade of cliffs, rocky hills near Chihuahua, X- 1885, *C.G. Pringle 621* (Isotype: MO-126274!; GH00053672!). Holotype not seen.

**Distinguishing features:** Subshrubby or perennial herb up to 0.8 m tall. Stems densely pilose. Young parts sparsely gland-tuberculate. Leaflets 5–10 pairs per leaf, dark-green, sometimes turning black when drying. Peduncles up to 7 cm long. Spikes 1–14 cm long. Bracts between flowers persistent. Flowers light-yellow, yellow-lemon or yellow-greenish, concolorous, the banner rubescent with age, the wings and keel turning brown or black.

**Representative examined material:** Coahuila: 2-IX-1941, *I.M. Johnston 8745* (LL); 31-x|VII-1973, *J.S. Henrickson 11690-A* (LL); 29-VIII-1971, *J.S. Henrickson 6127* (LL). Nuevo León: 16-IV-2001, *C. Yen y E. Estrada 12429* (CFNL); 13-VII-1989, *E. Estrada 1604* (CFNL, TEX-LL); 30-VII-2002, *C. Yen y E. Estrada 15539* (CFNL); 9-XI-2002, *C. Yen y E. Estrada 15184* (CFNL); 9-XI-2002, *C. Yen y E. Estrada 15167* (CFNL); 30-X-2002, *E. Estrada 15206* (CFNL); 16-IV-2001, *C. Yen y E. Estrada 12386* (CFNL). Tamaulipas: 20-IV-1985, *M. Martínez 363*, *M. Martínez*, *L. Hernández* (UAT); 13-X-1986, *M. Martínez 1354* (LL); 20-IX-1976, *F. González-Medrano 9927*, *A. Castellanos*, *P. Zavaleta* (UAT); 20-IX-1976, *F. González-Medrano 9872*, *A. Castellanos*, *P. Zavaleta* (UAT).

**Distribution:** Endemic to Mexico, from northern Coahuila to Tamaulipas, and extending southward from Zacatecas and San Luis Potosí to the State of Mexico and Puebla. Of the three infraspecific categories of this species (*D. l.* var. *lutea*, *D. l.* var. *arsenei*, and *D. l.* var. *gigantea* [29], only var. *lutea* is recorded in northeastern Mexico, the other two varieties are distributed south of the Tropic of Cancer. *Dalea lutea* is morphologically similar to *D. melantha* but easily differentiated by its persistent bracts between the flowers. Individuals of the three varieties of *Dalea melantha* in northeastern Mexico always have deciduous bracts between the flowers. In desert scrublands of low plains and mountain slopes, oak, oak-pine, and conifer forest, common along roads, mostly found between 700–2300 m.

***D. melantha*** S. Schauer var. ***berlandieri*** (A. Gray) Barneby, The New York Bot. Gard. 27: 485. 1977. Basionym: *Parosela berlandlerei* (A. Gray) Rose, Contrib. U. S. Nat. Herb. 10: 106. 1906.

**Type:** Mexico, Tamaulipas in mountains proper San Carlos, XI-1831, *J.L. Berlandier 2372 = 942* (Holotype: GH00053631!).

**Distinguishing features:** Shrub up to 1.6 m tall. Stems glabrous. Leaflets 2–3 pairs per leaf; racemes loose, the flowers separated from each other by almost the same width of their calyxes, its axis conspicuous. Flowers light yellow-green, turning dark brown (chocolate), purple or black. Calyx teeth longer than tube, narrowly triangular-subulate to aristiform, always plumose; keel and wing petals inserted below middle of staminal tube, 0.8–1.7 mm from base; wing petal 4.8–5.2 mm long.

**Representative examined material:** Coahuila: III-1905, *C.A. Purpus 1069* (NY 01279848!); 11-XI-1963, *H.D.D. Ripley 13515* (NY 01279849!). Nuevo León: 13-V-1992, *Hinton* et al. *21991* (TEX-LL); 21-VI-2003, *C. Yen y E. Estrada 15776* (CFNL); 13-IV-2003, *C. Yen y E. Estrada 15525* (CFNL). 22-III-2003, *C. Yen y E. Estrada 15353* (CFNL); 22-III-2003, *C. Yen y E. Estrada 15358* (CFNL); 11-V-2024, *E. Estrada 26230* (CFNL). Tamaulipas: 29-XII-1991, *E. Estrada 2400*, *2404* (CFNL, NY, TEX); 9-XII-1976, *F. Gonzlez-Medrano 10145* (UAT).

**Distribution:** Endemic to northeastern Mexico. Plains and slopes with highly calcareous soils. Easily recognizable by its leaves with only 2–3 pairs of glabrate leaflets per leaf, and its lax inflorescences. Frequent in chaparral, desert scrublands of the high plains, oak, oak-pine, and conifer forest, 1200–2000 m.

***D. melantha*** S. Schauer, Linnaea 20: 746. 1847. var. ***melantha***. Basionym: *Dalea melantha* S. Schauer, Linnaea 20: 746. 1847. *Parosela melantha* (S. Schauer) Rydb., N.L. Britton & al. (eds.), N. Amer. Fl. 24: 108. 1920. *Parosela fuscescens* Rydb., N.L. Britton & al. (eds.), N. Amer. Fl. 24: 109. 1920. *Dalea fuscescens* (Rydb.) Rzedowski, Ciencia (Mex.) 15: 93. 1955. *Dalea guadalcazarensis* Rzedowski, Ciencia (Mex.) 15: 92. 1955.

**Type**: Mexico, San Luis Potosí, 10 Km. al O de Guadalcázar, 1600 m. 1-X-1954, *J. Rzedowski 4911* (Holotype: MEXU01169314!).

**Distinguishing features:** Morphologically similar to the previous variety, with glabrous stems, but with 3–6 pairs of leaflets per leaf, up to 5 mm long, oblanceolate to obovate, truncate to retuse, the inflorescences in more compact spikes, and the calyces contiguous to each other, hiding the axis of the spike.

**Representative examined material:** Coahuila: Nuevo León: 12-IV-2003, *C. Yen y E. Estrada 15495* (CFNL); 21-VI-2003, *C. Yen y E. Estrada 15783* (CFNL); 12-IV-2003, *C. Yen y E. Estrada 15500* (CFNL); 2-III-2003, *C. Yen y E. Estrada 15221* (CFNL); 2-III-2003, *C. Yen y E. Estrada 15259* (CFNL). Tamaulipas: 13-X-1986, *M. Martínez 1354* (UAT).

**Distribution:** Endemic to Mexico, from Coahuila and Nuevo Léon to Querétaro, Puebla, and Oaxaca. In high plains of northeastern Mexico, frequent in calcareous soils, desert scrublands, oak, oak-pine, conifer forest, 1400–2200 m.

***D. melantha*** S. Schauer var. ***pubens*** Barneby, New York Bot. Gard. 27: 485. 1977.

**Type:** Mexico, Coahuila, ca. 26 (air) miles SE of Torreon in Sierra de Jimulco, ca 6 (air) miles SSW of La Rosita along trail to summit, 1 1/2 miles above roads’ end above main NE-SW running canyon. Near 25°10′ N 103°15′ W, 18-IX-1973, *J. Henrickson 13140* (Holotype: LL00371306!).

**Distinguishing features:** Morphologically similar to the two previous varieties, although this is the only one with conspicuous appressed pubescence on stems and leaflets.

**Representative examined material:** Coahuila: 17-IX-1989, *E. Estrada 1830* (CFNL, NY); 18-IX-1973, *J. Henrisckson 13140* (RSA0003258!).

**Distribution:** Endemic to the state of Coahuila. This is the variety with the most restricted distribution, only found in two areas of Coahuila (Sierra Maderas del Carmen, and Sierra de Jimulco). In desert scrublands and associations of chaparral-grasslands, 1900–2250 m.

***D. multiflora*** (Nutt.) Shinners, Field, and Lab. 17: 82. 1949. Basionym: *Petalostemon multiflorum* Nutt., Jour. Acad. Philad. 7: 92. 1834. *Kuhnistera multiflora* (Nutt.) A. Heller, Mem. Torrey Club 5: 197. 1894. *Kuhnistera candida multiflora* (Nutt.) Rydb., Contrib. U. S. Nat. Herb. 3: 154. 1895.

**Type:** USA, Red River, non date, *T. Nuttall n.n.* (Isotype: NY00026678!). Holotype not seen.

**Distinguishing features:** Herbaceous. Leaves 2–3 cm long. Leaflets 3–6 pairs per leaf. Petals white. Stamens 5, its free filaments as long as the staminal column, Bracts accompanied by persistent spiculiform or linear bracteoles, 0.5–1.6 mm long.

**Representative examined material:** Coahuila: 8-VII-1938, E.G. *Marsh 1252* (OKLA020016677!); 1-I-1935, E.G. *Marsh 30* (TEX00214206). Nuevo León: 23-VII-1933, *C.H. Muller & M.T. Muller 505* (TEX00214209).

**Distribution:** Widely distributed, from south-central USA to northeastern Mexico. This is the only species of herbaceous *Dalea*, erect and paniculately branched apically, with five stamens and free filaments as long as its staminal column in northeastern Mexico. Low plains with calcareous soils, commonly in desert scrublands, 370–650 m.

***D. nana*** Torr. ex A. Gray var. ***carnescens*** (Rydb.) Kearney & Peebles J. Washington Acad. Sci. 29: 483. 1939. Basionym: *Parosela carnescens* Rydb., Fl. Rocky Mts. 483. 1063. 1917. *Dalea carnescens* (Rydb.) Bullock, Kew Bull. 1939: 195. 1939. *Dalea rubescens* S. Watson, Proc. Amer. Acad. 17: 369. 1882. *Parosela rubescens* (S. Watson) Vail, Trans. N. Y. Acad. Sci. 14: 34. 1894. *Parosela elatior* Vail, Bull. Torrey Club 24: 15. 1897. *Dalea nana* var. *elatior* Gray ex B. L. Turner, Leg. Tex. 157. 1959. *Parosela whitehouseae* Tharp & F.A Barkley, An. Esc. Nac. Ci. Biol. 4: 285. 1946. *Parosela lesueuri* Tharp & F.A Barkley, An. Esc. Nac. Ci. Biol. 4: 286. 1946.

**Type:** USA [Texas], “in Pass of the Limpia”, northeast of Fort Davis, 24-VIII-1949, *C. Wright 124* (Lectotype: GH00053615!).

**Distinguishing features:** Herbaceous, perennial, gray, or silvery pilose. Leaves mainly 5-foliolate, rarely 3 to 7-foliolate. Inflorescence compact, its axis inconspicuous. Bracts 2.5–5.5 × 1.2–2 mm wide. Calyx 6.5 mm long or shorter, its teeth 2.5 times longer than the tube. Petals yellow, turning red with age; the banner up to 5.5 mm long; stems commonly diffuse.

**Representative examined material:** Coahuila: 19-IV-1900, *C.G. Pringle 9015* (NY 01305036); VIII-1880, *E. Palmer 227* (NY01074257!); 4-VII-1936, *F.L. Wynd 471* (NY01305033); 23-VI-1936, *F.L. Wynd 248* (NY 01305032). Nuevo León: 5-VII-2001, *E. Estrada 12891*. *C. Yen y E. Estrada 11872* (MEXU); 7-VII-2001, *C. Yen y E. Estrada 12987* (CFNL); 21-XI-1963, *H.D. Ripley*, *R.C. Barneby n.n*. (MEXU); 16-VI-1987, *E. Estrada 1226a* (MEXU). 23-VII-1989, *E. Estrada C. 1625* (TEX-LL). Tamaulipas: 25-XI-1984, *L. Hernández 1267* (UAT).

**Distribution:** Arizona and New Mexico (USA) to Chihuahua and Durango, as well as northeastern Mexico. The var. *nana* has relatively loose inflorescences, its rachis is visible between the flowers, its bracts are wider, 2–4 mm, and its calyx is bell-shaped. Species morphologically similar to *D. nana* var. *carnescens* are *D. wrightii* and *D. parrasana*; however, those have larger bracts, 6–12 mm long as well as calyx 6.5 or more mm long. Another related species is *D. aurea*; although its stems are erect, the petals do not turn red with age and the banner is longer, 6.2–8.6 mm long. In desert scrublands, 250–1600 m.

***D. nana*** Torr. ex A. Gray Torr. ex A. Gray, Mem. Amer. Acad. II, 4 (PI. Fendl. 1): 31. 1849. var. ***nana***. Basionym: *Parosela nana* (Torr.) A. Heller, Contrib. Herb. Franklin & Marshall Coll. 1: 49. 1895.

**Type:** USA, Sandy soil, Willow Bar, on the Cimmaron, 1-I-1847, *A. Fendler 130* (Lecotype: GH00053606!).

**Distinguishing features:** Herbaceous, perennial, mostly gray or silvery pilose; calyx teeth 2.5 times longer than the tube. Leaves mainly 5-foliolate, rarely 3 or 7-foliolate. Inflorescence axis conspicuous, inlorescence loose, flowers separated by at least 1 mm. Bracts 2.5–5.5 x 2–4 mm. Calyx commonly 6.5 mm long or shorter. Petals turning red with age; the banner up to 5.5 mm long; stems commonly diffuse.

**Representative examined material:** Nuevo León: 27-XI-1966, *H.D.D. Ripley 14784* (NY01305025!). Tamaulipas: 24-III-1944, *F.A. Barkley 14599B* (NY 01305026!).

**Distribution:** From Colorado and Oklahoma (USA) to northern Mexico. Found in central and northern regions of Nuevo León and Tamaulipas. It is different from var. *carnescens* by its conspicuous floral axes and by its wider bracts, 2–4 mm. In desert scrublands, 150–1600 m.

***D. neomexicana*** (A. Gray) Cory, Rhodora 38: 406. 1936 var. ***neomexicana***. Basionym: *D. mollis* var. *neomexicana* A. Gray, PI. Wright. 1: 47. 1852. *Parosela neomexicana* (Gray) A. Heller, Cat. N. Amer. PI. ed 2, 6. 1900. *P. mollis* var *neomexicana* (A. Gray) Macbr., Contrib. Gray Herb., New Ser. 65: 16. 1922.

**Type:** USA, Western Texas to El Paso, New Mexico, Hills beyond the Pecos and the Pass of the Limpia, 1-VIII-1849, *C. Wright 127* (Lectotype: GH00053605!).

**Distinguishing features:** Stems and leaves pubescent. Leaflets obovate, obovate-oblong, obtuse, cordate or truncate-cordate, its margins strongly wavy by the presence of submarginal glands, these 0.2–0.3 mm diameter, adaxially and abaxially pubescent. Pedicels with short glands, 0.15–0.4 mm long. Flowers in dense racemes. Calyx pedicellate, hairy, the trichomes 1–2.6 mm long, its teeth 3.5–5–7 mm long, the glands small but visible and prominent. Petals bicolored, white and crimson or lilac. Keel 4.5–7.1 mm long, its petals with their margins overlapping, one over the other, not fused at their outer margins.

**Representative examined material:** Coahuila: 17-IX-1940, *I.M. Johnston*, *C. Muller 1372* (LL00214257); 6-IX-1940, *I.M. Johnston*, *C. Muller 1009* (LL00214258).

**Distribution:** Distributed from southwestern USA (Arizona, and New Mexico to Texas) to northern Mexico (Sonora to Coahuila). Similar to var. *longipila*, although the margin of the leaflets in var. *longipila* is entire, the submarginal glands are 0.1–0.2 mm in diameter. In desert scrublands, including grasslands, 600–1600 m.

***D. neomexicana*** (A. Gray) Cory var. ***longipila*** (Rydb.) Barneby, Mem. New York Bot. Gard. 27: 159. 1977. Basionym: *Parosela longipila* Rydb., N. Amer. Fl. 24: 64. 1919. *Dalea longipila* (Rydb.) Cory, Rhodora 38: 406. 1936. *Dalea mollis* var. *longipila* B.L. Rob. ex Rydb. N. Amer. Fl. 24: 64. 1919.

**Type:** United States, expedition from Western Texas to El Paso, New Mexico, V-1849, *C. Wright 126* (Isotype: BM001042575!). Holotype not seen.

**Distinguishing features:** Leaflets margin entire, submarginal glands of 0.1–0.2 mm diameter, pubescent adaxially and abaxially. Pedicel glands relatively short, 0.15–0.4 mm long. Petals bicolored, white, and crimson or lilac. Calyx teeth 3.5–5.7 mm long, and the tube glands small but visible and prominent. Petal of the keel with their margins overlapping, one over the other, not fused at their outer margins.

**Representative examined material:** Coahuila: 15-X-1972, *P.A. Fryxell 2059* (01305067); 20-VIII-1968, *H.D.D. Ripley 14923* (NY01305058); 3-V-1959, *D. S. Correll 21393* (01305071). Nuevo León: 25-V-2001, *E. Estrada 12604* (CFNL, MEXU); 5-V-2003, *C. Yen*, *E. Estrada 15656* (CFNL); 5-VII-2001, *C. Yen y E. Estrada 12857* (CFNL); 5-VII-2001, *C. Yen*, *E. Estrada 12865* (CFNL).

**Distribution:** South-eastern Texas (USA) and northeastern Mexico. Similar to var. *neomexicana*, but the latter with the margin of leaflets strongly undulated due to the presence of submarginal glands, glands 0.2–0.3 mm diameter. In low and high plains, calcareous and gypsic soils. Mainly in desert scrublands, 300–1850 m.

***D. neomexicana*** (A. Gray) Cory var. ***megaladenia*** (Rydb.) Barneby, Mem. New York Bot. Gard. 27: 160. 1977.

**Type:** Mexico, Coahuila: W Coahuila, road from Guimbalete SE to Acatita via Laguna del Rey, 17-IX-1942, *R. Santos*, *R.M. Stewart 2646* (Holotype: NY00007017!).

**Distinguishing features:** Leaflets glabrous adaxially or only with trichomes on the margins. Pedicel glands large, 0.5–0.7 mm long. Calyx teeth 3.5 mm long or shorter, tube glands absent or almost invisible.

**Representative examined material:** Coahuila: 17-IX-1942, *R.M. Stewart 2646* (NY00007017!).

**Distribution:** Endemic to northeastern Mexico. This variety has the most restricted distribution of the species. Its leaflets are commonly glabrous adaxially. It has the shortest calyx size of the species, 3.5 mm long or shorter. Mainly in high plains with desert scrubland, 1000–1200 m.

***D. obovatifolia*** Ortega, Decades 32. 1797 var. ***obovatifolia***. Basionym: *Psoralea mutabilis* Cav., Descr. Pl.: 186. 1801. *Dalea mutabilis* (Cav.) Willd., Sp. PI. 3: 1339. 1801. *Parosela mutabilis* (Cav.) Cav., Descr. PI. 186. 1802. *Parosela attenuata* Rydb., N. Amer. Fl. 24: 82. 1920. *Dalea attenuata* (Rydb.) R.S. Cowan, Brittonia 8: 60. 1954.

**Type:** no typus found at MA; neotypus: “Ex regno Mexicano culta in hort. Mat. anno 1797”, MA (Barneby, 1977).

**Distinguishing features:** Herbaceous, annual, or rarely perennial. Stems purple. Leaflets 2–6 pairs per leaf, obovate, emarginate to oblanceolate, rarely short-acuminate. Bracts with awned apex, this equal to or longer than its body. Keel petals with their margins fused at their outer edges. Calyx tube 2.9–4.3 mm long. Stamens 7–10.

**Representative examined material:** Nuevo León: 26-X-1993, *Hinton* et al. *23762* (TEX-LL); 25-X-2003, *E. Estrada 15846* (CFNL); 25-X-2003, *E. Estrada 15861* (CFNL).

**Distribution:** Endemic to México and widely distributed from Nuevo León and San Lui Potosí to Jalisco and Puebla. Easily recognizable because it is the only herbaceous species of *Dalea* in northeastern Mexico with bicolored flowers (purple, blue-bronze, or violet combined with white petals), with bracts with an awn as long or as long, semi-straight, or sinuous as its body. High plains and mountains, in calcareous soils, commonly in oak, oak-pine, and conifer forests, 1500–600 m.

***D. parrasana*** Brandegee, Univ. Calif. Pub. Bot. 4: 179. 1911.

**Type:** Mexico, Coahuila, Sierra de Parras, X-1910, *C.A. Purpus 4741* (Holotype: UC148240!).

**Distinguishing features:** Herbaceous, perennial, strigose-pilose, the trichomes straight, subappressed mixed with few ascending hairs. Leaves mainly 5-foliolate, rarely 3 to 7-foliolate. Bracts 6–12 mm long. Calyx commonly longer than 7 mm, and its teeth 2.5 times longer than the tube. Petals mainly yellow, turning brown or pinkish-brown. Banner up to 6.3 mm long. Wings and keel petals inserted above middle of the staminal column.

**Representative examined material:** Coahuila: 24-X-1963, *H.D.D. Ripley 13282* (NY 01305195!); 18-X-1993, *A. Prahter 1495* (TEX00214292); 14-X-2010, *J. Hinton 29246* (TEX00452634). Nuevo León: 12-VIII-1988, *T.F. Patterson 6525* (TEX00214295).

**Distribution:** Endemic to northeastern Mexico, and San Luis Potosí. *D. wrightii* is morphologically similar to *D. parrasana*, although the latterhas a banner slightly longer, 7.3–9.5 mm long, and the wings and keel petals are inserted below middle of staminal column. Dry rocky or shaley hills, commonly on or near gypsum outcrops, stony slopes, adjacent to chalky and strongly calcareous soils, in desert scrublands, 1450–1850 m.

***D. pogonathera*** A. Gray, Mem. Amer. Acad. Arts, n.s., 4(1): 31. 1849. var. ***pogonathera*.** Basionym: *Parosela pogonathera* (A. Gray) Vall, Trans. N. Y. Acad. Sci. 14: 34. 1894.

**Type:** Mexico, near Monterrey, *L. A. Edwards & J. H. Eaton* 18 (Holotype: GH00053673!).

**Distinguishing features:** Herbaceous, perennial, its stems not lignified basally, and disappearing after fruiting in the following season. Leaflets, thick-textured, 2–4 pairs per leaf, oblanceolate—elliptic or linear-oblong, retuse. Calyx tube 2.6–3.3 mm long, the longest calyx tooth 4 mm long or longer; tooth trichomes 1.5 mm long or longer. Petals bicolored. Banner opening white or yellow, but rubescent with age, and neither stained yellow in the center nor small glands present, the wings and keel petals white, pink, purple, magenta, or violet, never yellow.

**Representative examined material:** Coahuila: 10-X-1993, *M.A. Carranza 1888* (UAT); 14-IV-1997, *J. Henrickson 22080* (ANSM); 18-IV-2017, *J. A. Encina 5755* (ANSM); 5-XII-1997, *M. A. Carranza C-2855* (ANSM); 18-IX-1990, *R. Vásquez 129* (ANSM). Nuevo León: 10-X-1993, *G. B. Hinton 23578* (ANSM); 28-IX-1996, *Hinton* et al. *25894* (TEX-LL).

**Distribution:** Southern USA (Arizona, New Mexico, and Texas) to North Mexico (Chihuahua to Nuevo León, including Durango, San Luis Potosí, and Zacatecas). Of the two varieties of this species distributed in northeastern Mexico, var. *pogonathera* has wider inflorescences, 1.4–2 cm, and longer calyx teeth, always longer than 4 mm, while var. *walkerae* has calyx teeth less than 4 mm long and thinner inflorescences, up to 1.2 cm wide. In alluvial fans and high plains, in desert scrublands, 450–1750 m.

***D. pogonathera*** A. Gray var. ***walkerae*** (Tharp. & Barkley) B.L. Turner, Field & Lab. 24: 16. 1956. Basionym: *Parosela walkerae* Tharp & Barkl., Bull. Torrey Club 73: 133. 1946. *Dalea penicillata* Moric, PI. Nouv. d’Amer. 66, minore pro parte. Tab. XLV, Figure 2. 1839.

**Distinguishing features:** Almost identical to the previous variety, although, the width of its inflorescence and the size of the calyx teeth can be useful for its discrimination (see distribution on var. *pogonathera*).

**Representative examined material:** Coahuila: non date, *J. A. Villarreal 7090* (ANSM); 17-IX-1992, *J.A. Villarreal 6910* (ANSM); 11-X-1991, *M. A. Carranza C-980* (ANSM); 30-III-1992, *M. A. Carranza 1428* (ANSM). Nuevo León: 7-VII-2001, *C. Yen y E. Estrada 12986* (CFNL); 22-X-2002, *C. Yen y E. Estrada 15165* (CFNL); 7-VII-2001, *C. Yen y E. Estrada 13029* (CFNL); 30-IX-1955, *M.C. Johnston 2796A* (TEX-LL); 26-V-1966, *J.S. Wilson 10829* (TEX-LL); 28-VII-2002, *C. Yen y E. Estrada 15145* (CFNL); 17-IV-2001, *J. Luna*, *C. Yen y E. Estrada 12479* (ANSM). Tamaulipas: *J.L. Berlandier 2444* (NY01277203!); 30-X-1964, *H.D.D. Ripley 13597* (NY 01305306).

**Distribution:** From Texas (USA) to northeastern Mexico, mainly in low plains, 100–650 m, in desert scrublands, and mezquitales.

***D. prostrata*** Ortega, Nov. Pl. Descr. Dec. 69. 1798. Basionym: *Parosela triphylla* (Sessé & Moc. ex G.Don) J.F. Macbr. Contr. Gray Herb. 65: 18. 1922. *Dalea trifoliolata* Moric. Pl. Nouv. Amér.: 3 1834. *Dalea triphylla* Pav. ex Schltdl. Linnaea 12: 280. 1838. *Dalea triphylla* Sesse & Moc. ex G. Don, Gen Hist. Diehl. PI. 2: 224. 1832. *Dalea triphylla* Sessé & Moc. ex G. Don, Gen. Hist. 2: 224. 1832. *Parosela trifoliolata* (Moric.) Rydb. N. Amer. Fl. 24: 99. 1920.

**Type:** No type survive at MA (Barneby, 1977).

**Distinguishing features:** Herbaceous perennial, prostrate, mostly gray or silvery pilose, the trichomes ascending. Leaves almost always 3-foliolate, rarely 5-foliolate. Leaflets obovate to elliptic-obovate. Calyx teeth 2.5 times longer than the tube, its intercostal spaces with a row of 3–4 small glands. Petals yellow, light-yellow. Banner blade 2.6 mm long or longer, its claw 2 times longer than blade.

**Representative examined material.** Nuevo León: 13-X-1986, *Hinton* et al. *19079* (TEX-LL). Tamaulipas: XII-1930, *H.W. Viereck 981* (US 02319895!).

**Distribution:** Endemic to Mexico. North and central Mexico, from Chihuahua and Durango, to Tamaulipas, extending south to Guanajuato, Mexico City, Querétaro, Hidalgo, and western Michoacán. Morphologically similar to *D. laniceps*, but the latter with a banner blade up to 2.2 mm long and its claw 2–3 times longer than blade. High plains and hillsides, frequent in desert scrublands, particularly in arid grasslands, 1300–2650 m.

***D. radicans*** S. Watson, Proc. Amer. Acad. 17: 341. 1882. Basionym: *Parosela radicans* (S. Watson) Rose, Contrib. U. S. Nat. Herb. 8: 305. 1905.

**Type**: Mexico [Coahuila], Sierra Madre, 40 miles south of Saltillo, VII-1880, *E. Palmer 214* (Holotype: GH00053679!).

**Distinguishing features:** Dwarf shrub, 10–20 cm in height, stems erect to suberect, rooting in the apical part of the branches, stems and leaves glabrate. Petals bicolored, the banner opening white or yellow, but rubescent with age, wings, and keel petals white, pink, purple, magenta, or violet, with scattered tiny glands.

**Representative examined material.** Coahuila: 12-VII-2016, *J. A. Encina 5589* (ANSM); 26-IV-2015, *J.A. Encina 4547* (ANSM); 27-VII-1979, *D. Arredondo 129* (ANSM); 02-XI-1988, *J.A. Villarreal 4800* (ANSM); 25-IX-1991, *J.A. Villarreal 6296* (ANSM). Nuevo León: 10-V-2003, *C. Yen y E. Estrada 15566* (CFNL); 19-VII-1999, *E. Estrada 10297* (CFNL).

**Distribution:** Endemic to northeastern Mexico. This species is similar to *D. gypsophila*, but the latter has rhizomatous stems, adventitiously rooting in the basal parts. In addition, the leaflets have tiny white papillae, its calyx has silky pubescence, and its petals without tiny, scattered glands. Calcareous, limestone, and gypsum soils, in desert scrublands, and oak-pine forest, 1600–2300 m.

***D. saffordii*** (Rose) Bullock, Kew Bull. 1939: 198. 1939. Basionym: *Parosela saffordii* Rose, Contrib. U. S. Nat. Herb. 12: 273. 1909.

**Type:** Mexico, [Coahuila], 3-II-1907, *W.E. Safford*, *1246* (Holotype: US00003782!).

**Distinguishing features:** Shrubs, erect 0.5–1.5 m tall. Stems glabrate, without verrucose or tuberculated glands. Leaves 10–16 mm long with 6–9 pairs of crowded, folded leaflets. Calyx pubescent, its teeth longer than the tube. Flowers 10 or more in elongated racemes, raceme axis 5–15 mm long. Calyx 4.8–7.7 mm long, and its teeth 2.3–4.7 mm long. Petals bicolored, the banner opening white or yellow, but rubescent with age, wings, and keel petals white, pink, purple, magenta, or violet.

**Representative examined material**. Coahuila: *A. Rodríguez y M. de la Rosa 541* (ANSM); 05-VI-1992, *J.A. Villarreal 6585* (ANSM); 09-X-1993, *M.A. Carranza 1786* (ANSM); 20-VI-1984, *J.A. Villarreal 2843* (ANSM); 21-III-1992, *J. A. Villarreal 6419* (ANSM). Nuevo León: 12-IV-2003, *C. Yen y E. Estrada 15493* (ANSM, CFNL, MEXU); 16-III-1993, *Hinton* et al. *22715* (TEX-LL); 15-V-1985, *F. González Medrano 14661* (MEXU, TEX-LL); 11-V-1989, *E. Estrada C. 1463* (CFNL, MEXU, TEX-LL); 10-V-2003, *C. Yen y E. Estrada 15580* (ANSM, MEXU); 15-V-2003, *C. Yen y E. Estrada 15630* (CFNL); 2-III-2003, *C. Yen y E. Estrada 15238* (CFNL); 10-V-2003, *C. Yen y E. Estrada 15580* (CFNL); 21-V-1992, *J. A. Villarreal 7177* (ANSM).

**Distribution:** Restricted to northeastern Mexico. *Dalea formosa* is another species morphologically related to *D. saffordii*, both have stems without warty glands, pubescent calyx, and the calyx teeth are longer than the tube. However, *D. formosa* has leaves 2.5–11 mm long with 3–7 pairs of unfolded leaflets; subcapitate racemes, with their axis up to 9 mm long and with only 2–9 flowers, its calyx 7.4–16.2 mm long and its teeth 4.5–8.5 mm long. In mountain slopes, oak-pine forests, and chaparral, 2200–2700 m.

***D. scandens*** (Mill.) R.T. Clausen var. ***paucifolia*** (J.M. Coult.) Barneby, Mem. New York Bot. Gard. 27: 527. 1977. Basiyonym: *Dalea domingensis* var. *paucifolia* Coult., Contrib. U. S. Nat. Herb. 1: 34. 1890. *Psoralea humilis* Mill., Gard. Diet., ed. 8, *Psoralea* No. 7. 1768. *Parosela humilis* (Mill.) Rydb., N. Amer. Fl. 24: 114. 1920. *Dalea thyrsiflora* A. Gray, Proc. Amer. Acad. 5: 177. 1861. *Parosela thyrsiflora* (A. Gray) Vail, Bull. Torrey Club 24: 14. 18971. *Dalea emphysodes* subsp. *thyrsiflora* (A. Gray) R. T. Clausen, Bull. Torrey Club 73: 85. 1946. *D. carthagenensis* subsp. *thyrsiflora* (A. Gray) R. T. Clausen, Bull. Torrey Club 73: 572. 1946.

**Type:** Mexico, Tamaulipas, XI-1830, *J.L. Berlandier 846 (=2266)* (Lectotype: GH00053684!)

**Distinguishing features:** Small shrub, up to 1.5 m tall. Leaves up to 4.6 cm long. Leaflets 7–11 pairs per leaf, mostly obovate. Inflorescences sessile, its apex slightly tilted. Dorsal teeth of the calyx hooked apically. Petals greenish-white or cream-colored at the beginning of anthesis, soon turning dull red-violet, its keel petals with their margins fused at their outer edges.

**Representative examined material:** Nuevo León: 16-V-2003, *C. Yen y E. Estrada 15657* (CFNL); 01-X-2011, *E. Estrada 21227* (ANSM); 9-XI-1959, *J. Graham & M.C. Johnston 4245* (MEXU). 4-VII-2002, *C. Yen y E. Estrada 14863* (CFNL), 28-XI-1964, *H.D. Ripley & R.C. Barneby 13551* (MEXU). Tamaulipas: 29-IV-1985, *M. Martínez 493* (UAT); 30-IV-1985, *M. Martínez 615* (UAT); 14-XI-1993, *A. Mora-Olivo 4894* (UAT); 25-XI-1985, *R. Diaz 206* (UAT); 18-XI-1992, *J.L. Mora-López 382* (UAT; 25-XI-1984, *L. Hernández 1295* (UAT); 21-X-1983, *L. Hernández* et al. *797* (UAT); 21-X-1984, *D. Baro* et al., *600* (UAT).

**Distribution:** Widely distributed from Texas (USA) throughout the states of the Gulf of Mexico, northeastern Mexico, and San Luis Potosí to Yucatán, extending to Cuba. In northeastern Mexico, *D.* s. var. *paucifolia* is the only species whose dorsal tooth of the calyx is sigmoid in shape, culminating in a hook-shaped (uncinate). In low deciduous forests, evergreen tropical forests, and desert shrublands, 20–800 m.

***D. uniflora*** (Barneby) G.L. Nesom, Phytologia 73(5): 427. 1992. Basionym: *Dalea eriophylla* Barneby var. *uniflora* Barneby, Sida 10(1): 14. 1983.

**Type:** Mexico, Nuevo León, On open pine slope 4 miles south of Pablillo, 20-VII-1958, D. S. Correll & I. M. Johnston 19,903 (Holotype: LL00371300!).

**Distinguishing features:** Dwarf shrub, stems suberect to decumbent, 15–35 cm tall. Densely white-satiny and appressed-pubescent, foliage abundant, tiny satiny-silky-pubescent. Leaves palmate-trifoliolate, 2.2–6 mm long. Leaflets linear, tightly revolute, cylindrical in appearance. Inflorescences distal of only one flower, barely visible, surrounded, and almost covered by the distal leaves.

**Representative examined material:** Nuevo León: 12-IV-2003, *C. Yen y E. Estrada 15498* (CFNL); 19-VII-1984, *G. B. Hinton 18752* (MEXU); 08-IX-1989, *E. Estrada 1773* (MEXU); 27-VII-1989, *E. Estrada 1615* (CFNL, TEX-LL).

**Distribution:** Endemic to the south-central part of Nuevo León. Species morphologically similar to *D. uniflora* is *D. eriophylla*, but the latter has inflorescences in distal compact, subcapitate racemes of 3–6 flowers, almost covered (but visible) by distal leaves. In gypsum or strongly calcareous soils, oak-pine, pinyon-pine forests in semi-arid areas and chaparral of Rosaceae and Fagaceae, 1900–23,000 m.

***D. wrightii*** A. Gray, PI. Wright. 1: 49. 1842. Basionym: *Parosela wrightii* (A. Gray) Vail, Bull. Torrey Club 24: 16. 1897. *Dalea sabulicola* Brandegee, Univ. Calif. Publ. Bot. 4: 179. 1911. *Parosela warnockii* Tharp & F.A.Barkley) B.L.Turner, Sida, Bot. Misc. 24: 7. 2003.

(Coah., N.L.).

**Type:** USA, Western Texas to El Paso, New Mexico, 1849, *C. Wright 134* (Syntype: GH00053625!). Holotype not found.

**Distinguishing features:** Dwarf, herbaceous, perennial, mostly gray or silvery pilose. Calyx teeth 2.5 times longer than the tube. Leaves mainly 5-foliolate, rarely 3 to 7-foliolate. Petals yellow, sometimes turning orange to brown with age, often dull pink when dry. Bract 6–12 mm long. Calyx commonly longer than 7 mm long. Banner 7.3–9.5 mm long. Wings and keel petals inserted below middle of androecium.

**Representative examined material:** Coahuila: 25-VI-2007, *J. A. Encina 2509* (ANSM) 10-IX-1991, *M. A. Carranza C-1110* (ANSM); 08-IX-1990, *M. A. Caranza C-736* (ANSM); 05-X-1985, *A. Rodríguez 1466* (ANSM); 07-X-1989, *A. Rodríguez 1283* (ANSM); 13-X-1989, *J. A. Villarreal 5459* (ANSM); 09-V-1992, *J. A. Villarreal 6643* (ANSM); 08-IX-1990, *R. Vásquez 165* (ANSM)Nuevo León: 15-V-2004, *E. Estrada 16034* (ANSM); 1-X-1993, *M. A. Carranza 1727* (ANSM); 7-XII-2000, *J.A. Villarreal* et al. *9058* (ANSM, MEXU).

**Distribution:** Widely distributed in southern USA (Arizona to Texas) and northern Mexico (Sonora to Nuevo León). The wings and keel petals inserted below middle of the staminal column. The banner 7.3–9.5 mm long. *Dalea wrightii* is morphologically similar and often confused with *D. parrasana*, although, the latter species can be differentiated by its shorter banner, up to 6.3 mm long and the keel and the wings petals are inserted above middle of the staminal column. In low hills, desert scrubland (300–450 m) to grasslands and desert scrublands (800–1700 m).

***Eysenhardtia*** Kunth, Nov. Gen. Sp. 6: 489. 1824. Basionym: *Varennea* DC., Prodr. 2: 522 (1825) *Viborquia* Ortega, Nov. Pl. Descr. Dec.: 66. 1798. *Wiborgia* Kuntze, Revis. Gen. Pl. 1: 213. 1891. nom. illeg.

**Type:** Eysenhardtia amorphoides Kunth = E. polystachya (Ortega) Sarg.

Trees or shrubs. Leaves compound, imparipinnate. Petiolules with a basal gland. Leaflets glandular-punctate. Inflorescences in racemes or spiciform racemes. Calyx tube oblique, entire or incised, its teeth unequal in size, the ventral one the longest. Corolla partially irregular, sub-papilionate. Petals 5, free, white, inserted basally in the hypanthium, gradually narrowing towards the base. The banner, the widest petal, obtuse, truncate, or retuse. Stamens 10, diadelphous, the ventral one is the longest and free of the remaining 9 stamens, which are fused for at least half their length in an apically oblique tube, with alternating short and long filaments. Ovary sessile, glabrate. Ovules 2. Style pubescent, curved near the tip with or without an evident gland at the bend. Stigma capitate. Fruit oblong, ovate to oblong-oblanceolate, laterally compressed, rarely globose, indehiscent. Seed one.

A typically Mexican genus, distributed from southern USA through Mexico to Guatemala and El Salvador. According to the last two species additions to the flora of Mexico, *E. officinalis* [36] and *E. byei* [37], the genus is composed of 13 species. Four species are known in northeastern Mexico.
1A.Leaflets 7–13; stipels absent; style eglandular; racemes 1 cm long or shorter; fruits always ascending ***E. parvifolia***1B.Leaflets 15 or more; stipels present; style glandular or eglandular; racemes 2 cm long or longer; fruit ascending or reflexed22A.Leaflets with glands of two sizes, the large ones adjacent to the leaf margin and in parallel lines on both sides of the midvein, the smaller glands dispersed between the midvein and the margin; fruits always ascending***E. texana***2B.Leaflets with glands of the same size33A.Leaflets with revolute margin, the midvein sunken; calyx not split to the base in fruit stage; fruit 3–4 times longer than wide***E. polystachya***3B.Leaflets with entire margin, the midvein not sunken; calyx split to the base in fruit stage; fruit less than 3 times longer than wide***E. schizocalyx***

***Eysenhardtia parvifolia*** Brandegee, Univ. Calif. Publ. Bot. 4: 180. 1911.

**Type:** México, Coahuila, Sierra de Parras, X-1910, C.A. Purpus 5074 (Holotype: UC150014!).

**Distinguishing features:** Small shrub up to 1 m tall, intricately branched. Leaflets 7–13 per leaf, bicolored, gland-dotted, the glands located in a line along each side of midvein. Stipels absent. Inflorescences short, 1 cm or shorter. Fruits ascending, gland-dotted with large glands.

**Representative examined material**: Coahuila: 6-IX-1988, *Villarreal 4485* (ANSM, ASU0035784!). Nuevo León: 13-V-1992, *Hinton* et al. *21985* (TEX). Tamaulipas: 23-VIII-1984, *F. González-Medrano 283* (UAT); 23-V-1976, *F. González-Medrano 9024* (UAT).

**Distribution:** Endemic to Mexico, in the north of Mexico from Chihuahua to Tamaulipas, and San Luis Potosí. Easily distinguishable from the rest of the remaining *Eysenhardtia* species in northeastern Mexico, because all have more than 15 leaflets per leaf, stipels present, and racemes of 2 cm long or longer. Desert scrubland, 1400–1600 m, rare.

***E. polystachya*** (Ortega) Sarg., Silva. N. Amer 3: 29. 1891. Basionym: *Viborquia polystachya* Ortega, Nov. Rar. Pl. Hort. Matr. Descr. 5: 66. Tab 9. 1798. *Verennea polystachya* (Ortega) DC., Prod. 2. 522. 1825. *Wiborgia polystachya* (Ortega) Kuntze, Rev. Gen. Pl. 1: 213. 1891. *E. amorphoides* Kunth, Nov. Gen. Sp. Plantarum ed. Quar.6: 491. Tab 592. 1824. *Dalea fruticosa* G. Don, Gen. Hist. Pl. 2: 226. 1832. *Psoralea fruticosa* Sessé & Mociño, Pl. Nov. Hisp. 121. 1889.

**Type:** Mexico, S. Augustin [Nov. gen. sp.: Crescit in Regno Mexicano, prope San Augustin de las Cuevas et Guanaxuato], non-date, *A.J.A. Bonpland*, *F.W.H.A. von Humboldt 4128* (Holotype: P00659956!).

**Distinguishing features:** Shrub or tree up to 7 m tall. Stipels longer than petiolules. Leaflets 20–61, the midvein sunken, the margin not revolute, bicolored, darker adaxially, gland-dotted, the glands of the same size abaxially. Inflorescences in racemes 3–13 cm long. Calyx shallow split on the dorsal side. Fruit reflexed-spreading, with small or tiny glands apically.

**Representative examined material**: Tamaulipas: 24-IX-1984, *R. Díaz P. 60* (UAT); 6-VIII-1994, *D. Seigler 14240* (UAT); 21-IX-1984, *P. Hiriart*, *F. G. Medrano*, *Deborah Baro 386* (UAT); 10-V-1985, *R. Díaz 407* (UAT); 23-IX-1984, *F. González-Medrano 14221* et al. (UAT); 21-VI-1985, *M. Yanez 254* (UAT); 21-II-1998, *M. Galván 705* (UAT); 22-VI-1996, *C. Ramos n.n.* (UAT).

**Distribution:** Endemic to Mexico, one of the species of this genus with the wider distribution, from Durango to Tamaulipas, extending south to Oaxaca. The presence of this species has been reported for the states of Nuevo León and Coahuila, although to date we have not recorded herbarium samples with this species. Low deciduous forest, oak-pine forest, cloud forest, chaparral, and disturbed areas, 1400–2200 m.

***E. schizocalyx*** Pennell, N. Am. Fl. 24: 39. 1919.

**Type:** Mexico, Durango, Mapimi, Durango, 21/23-X-1898, *E. Palmer 528* (Holotype: NY00008072!).

**Distinguishing features:** Shrub to low tree, up to 6 m tall. Leaflets with flat margins, the midvein not sunken, gland-dotted, the glands of the same size, the midvein not sunken. Calyx split to the base in the fruit stage; fruit less than 3 times longer than wide, ascending. Inflorescences in racemes, 5–9.8 cm long.

**Representative examined material**: Coahuila: 17-IX-1973, *J. Henrickson 13068* (LL00432446); 28-VI-1941, *L. R. Standford*, *K. L. Retherford*, *R. D. Northcraf n.n.* (ARIZ120447).

**Distribution:** Endemic to northern Mexico (Chihuahua, Durango, and Coahuila. In low arid mountains, desert scrublands, along stream beds, 700–2000 m.

***Eysenhardtia texana*** Scheele, Linnaea 21: 462. 1848. Basionym: *Eysenhardtia angustifolia* Pennell, N. Amer. Fl. 24: 38. 1919.

**Type:** USA, Texas, “An hohen Ufferand and trocken Platzen in der Nahe des Wasser bei Neubraunfels”, 19-VII-1846, *J.L. Ferdinand 268* (Holotype: MO256443).

**Distinguishing features:** Shrub or tree up to 7 m tall. Leaflets with glands of two sizes, the large ones adjacent to the leaf margin, in parallel lines on both sides of the midvein, the smaller glands dispersed between the midvein and the margin. Fruits always ascending.

**Representative examined material**: Coahuila: 17-VIII-1971, *L. McGill 7851* (ASU0035791!); 25-VI-1979, *M. Mittleman 26* (ASU0035794!); 10-VIII-1970, *E. Meyer 67272* (ASU0035795!). Nuevo León: 12-IV-2003, *C. Yen y E. Estrada 15514* (CFNL); 21-VI-2003, *C. Yen y E. Estrada 15785* (CFNL); 12-IV-2003, *C. Yen y E. Estrada 11510* (CFNL); 2-VII-2004, *C. Yen y E. Estrada 11638* (CFNL); 3-VII-2003, *C. Yen y E. Estrada 13053* (CFNL). Tamaulipas: 18-XI-1992, *J.L. Mora-López 387* (UAT); 3-X-1984, *D. Baro*, *C. Aguilar*, *S. Rodríguez*, *R. Fuentes 490* (UAT); 18-IX-1976, *F. González-Medrano 9819* (UAT); 21-VI-1985, *D. Méndez 43* (UAT); 20-IX-1975, *F. González-Medrano 9895* (UAT).

**Distribution:** From central Texas, USA to northeastern Mexico, extending to San Luis Potosí and Veracruz. Easily distinguishable from the rest of the species from northeastern Mexico, because has glands of two different sizes on leaflets. Frequently confused with *E. polystachya*, but the latter has leaflet glands of similar size. In desert scrublands, oak, oak-pine, conifer forest, 360–2200 m.

***Marina*** Liebm., Vidensk. Meddel. 1853: 103. 1853.

**Type:** *Marina gracilis* Liebm., Vidensk. Meddel. Naturhist. Foren. Kjøbenhavn 1853: 103. 1853.

Herbaceous or shrubs. Leaves imparipinnate. Leaflets with parallel white sinuous lines ascending from the middle vein adaxially and sometimes abaxially. Inflorescences in racemes moderately dense or loose, never compact, capitate, or conical. Pedicels accompanied by a pair of glands at the base and another pair above the middle. Calyx campanulate, 5-toothed and with 10 ribs, ribs of the calyx without anastomosing or without ever reaching the apex of each of the teeth. Petals 5, one, the banner, inserted on the hypanthium, the other four, the keel and the wings, inserted near base, medially or apically on the staminal column. Stamens 5–10, monadelphous. Fruit small, immersed or slightly protruding from the calyx, compressed, oblique-ovoid but compressed, and inflated but obovoid, always with glands, these forming crescent-shaped patterns or with scattered glands in irregular patterns. Ovules 1.

Almost a Mexican genus, composed of 38 species [29]), and one recently added (*Marina filiciformis* [21], distributed from southwest USA (California and New Mexico) to Guatemala and Venezuela, with number of species in the south, west, and northwest of Mexico, rare and with few species in northeastern of Mexico.
1A.Calyx pubescent, its dorsal tooth 3.7–7.5 mm long, ***M. filiciformis***1B.Calyx glabrous, its dorsal tooth 1.4–2.6 mm long22A.Banner 2.6–3.8 mm long, not peltate, its basal lobes opened, not attached at the top of the claw ***M. scopa***2B.Banner 3.6–5.2 mm long, peltate, its basal lobes attached across the top of the claw***M. nutans***

***Marina filiciformis*** (B.L. Rob. & Greenm.) Piñeros-U. & F. González, Caldasia 45(1): 49–65. 2023. Basionym *Dalea filiciformis* B.L. Rob. & Greenm., Proc. Amer. Acad. 29: 382. 1894. *Parosela filiciformis* (B.L. Rob. & Greenm.) Rose, Contrib. U. S. Nat. Herb. 8: 303. 1905.

**Type**: Mexico, Villar [=Puerto de San Jose, a railroad halt on the Tampico line ± 50 mi n.-e. of San Luis Potosí San Luis Potosí], 14-IX-1893, *C.G. Pringle 5472* (Holotype: GH00053645!).

**Distinguishing features:** Herbaceous, perennial. Stems gland-tuberculate. Leaflets 5–14 pairs per leaf, ovate, orbicular, oblong-ovate, broad-ovate, obtuse, or retuse. Inflorescences in terminal long spikes, far surpassing the leaves, 4–27 cm long, the flowers in loose racemes, separated from each other by at least 2–3 times the length of the calyx. Flowers pedicellate, pink, lavender, or purple, sometimes turning brownish with age. Calyx pubescent, its dorsal tooth 3.7–7.5 mm long. Keel-petals with their margins overlapping, one over the other (not fused).

**Representative examined material**: Nuevo León: 13-IV-2003, *C. Yen y E. Estrada 15524* (CFNL); 10-V-2003, *C. Yen y E. Estrada 15570* (CFNL); 21-VI-2003, *C. Yen y E. Estrada 15791* (CFNL); 10-V-2003, *C. Yen y E. Estrada 15565* (CFNL); 22-III-2003, *C. Yen y E. Estrada 15342* (CFNL).

**Distribution**: Recent molecular studies have shown that *Dalea* is paraphyletic with respect to *Marina*, because *D. filiciformis* is sister to *Marina* [21]. Endemic to Mexico, from the northeastern region, recorded only in Nuevo León, and extending its distribution to Oaxaca. *Marina filiciformis* is quite distinctive from the other species of the genus, due to the conformation of its keel petals, imbricated (overlapping) and adhered by their margins, carunculous and glaucous leaflets, long, loose racemose inflorescence, and comparatively long flowers. Mostly in plains, hills, and mountains with gypsum soils, associated with gypsophilous grasslands, *Pinus arizonica*, and *P. cembroides* forest, oak forest, and desert scrublands, 1500–2400 m.

***M. nutans*** (Cav.) Barneby, Phytologia 26: 2. 1973. Basionym: on *Psoralea nutans* Cav., Icones 3: tab. 201.1794. *Dalea nutans* (Cav.) Willd., Sp. PI. 3: 1339. 1801. *Parosela nutans* (Cav.) Rose, Contrib. U. S. Nat. Herb. 8: 306. 1905. *Dalea pulcherrima* Sessé & Moc. ex G. Don, Gen. Hist. Diehl. PI. 2: 225. 1832. *Parosela submontana* Rose, Contrib. U. S. Nat. Herb. 8: 306. 1905. *Dalea submontana* (Rose) Bullock, Kew Bull. 1939: 198. 1939.

**Type:** Mexico, Mejico y en Horto R. Matr., XII-1793, *not collector*, *n.n.* (Holotype: MA476146!, material seen but labeled as Type of *Psoralea nutans*, not Holotype, as mentioned by [29].

**Distinguishing features:** Herbaceous perennial or suffruticose, glabrous. Stem erect, rarely diffuse, simple to near middle, paniculate branched apically. Leaves up to 5 cm, subsessile. Leaflets 8–20 pairs. Racemes loose, the flowers ascending then reflexed with age. Flowers bicolored, pink, magenta, or violet, mixed with white. The banner peltate, its basal lobes attached across the top of the claw, commonly opening bicolored, white, and purple, but soon rubescent. Stamens 10. Fruit subglobose, with many sub-agglomerated blister glands.

**Representative examined material:** Tamaulipas: 15-XI-1931, *H.W. von Rozynski* (MICH1171804; NY01307228).

**Distribution:** Endemic to Mexico, rare in the northern part of the country, recorded only in the mountains of Chihuahua and Tamaulipas, 1700 m. Frequent from Sinaloa, Durango and San Luis Potosí to south to Guerrero, Puebla, and Oaxaca. Almost always in mountain, oak-pine forest, 1600–2400 m.

***M. scopa*** Barneby, Mem. New York Bot. Gard. 27: 134. 1977.

**Type:** Mexico, Oaxaca, roadside weed at 1740 m, on mountain road e. of Teotitlan del Camino, 18-XI-1966, *Ripley & Barneby 17729* (Holotype: NY00016286!).

**Distinguishing features:** Herbaceous, annual. Stems simple basally and paniculately branched apically, up to 1.5 m tall, glabrous. Leaves 2.8–7.7 cm long. Leaflets 8–20 pairs, its rachis winged. Racemes loose with 2–13 flowers. Flowers bicolored, the banner white and magenta-purple like the wings and keel. The banner not peltate, its basal lobes opened, not attached at the top of the claw. Stamens 10. Fruit inflate, obovoid, ventrally crested, with a dorsal rounded suture, and with many large blister glands.

**Representative examined material:** Nuevo León: 25-X-2003, *E. Estrada 15831* (CFNL); 22-X-1981, *J.M. Poole 2406* (TEX-LL). Tamaulipas: 24-XI-1966, *H.D.D. Ripley 14755* (NY01307419; MICH 1171918).

**Distribution:** Widely distributed from north of Mexico to Venezuela. In oak-pine forest, 1300–1800 m.

***Psorothamnus*** Rydb., N. Amer. Fl. 24: 45. 1919. *Asagraea* Baill., Adansonia 9: 232. 1870. nom. illeg. *Psorodendron* Rydb., N. Amer. Fl. 24: 41. 1919.

**Type**: *Psorothamnus emoryi* (A. Gray) Rydberg, N. Amer. Fl. 24: 47. 1919.

Sub-shrubs (stoloniferous), shrubs, and trees. Stems retrorse with abundant glands (lens-like), pubescent, rarely glabrate, branches sometimes spinose. Leaflets trifoliolate, rarely unifoliolate or pinnate, 2–8 pairs per leaf, articulate. Inflorescences in racemes or spikes. Pedicels bracteolate or with a pair of sessile glands, sometimes both. Calyx campanulate, the ventral pair of teeth broader and almost always longer than the others, and frequently partly united behind banner. Petals inserted on hypanthium rim, pink, purple, violet, or vivid blue, glabrous or pubescent outside, occasionally gland-tipped. Banner with a linear short claw. Keel as long or longer than banner, rarely shorter, its petals overlapping by their external margins and adherent, enclosing the androecium. Stamens 10, the filaments alternate 5 often shorter. Ovules 2, Seeds 1–2.

A genus with 9 species [29] found in desert lands, from the central-west to southern USA (from Nevada and Utah to Arizona, California and New Mexico) to northern Mexico (Baja California, Sonora, Chihuahua, and Coahuila). Rare in the study area, recorded only in the state of Coahuila.

***Psorothamnus scoparius*** (A. Gray) Rydb, N. Amer. Fl. 24: 48. 1919. Basionym: *Dalea scoparia* A.Gray, Mem. Amer. Acad. Arts, n.s., 4(1): 32. 1849. *Dalea scoparia* f. *suberosea* Cockerell, Science, n.s., 7: 625. 1898. *Parosela scoparia* (A.Gray) A. Heller, Cat. N. Amer. Pl., ed. 2: 7. 1900. *Parosela scoparia* f. *arsenei* Standl., Publ. Field Mus. Nat. Hist., Bot. Ser. 17: 195. 1937.

**Type:** USA, Jornado del Muerto [between Santa Fe and El Paso del Norte], VIII, *F. A. Wislizenus 86* (Holotype: GH00053619!).

**Distinguishing features:** Shrub, up to 1 m tall. Stems many, branched, erect-incurved, decumbent, densely gray-strigose. Leaves up to 2 cm long, mainly simple, with a petiolule and a terminal, linear-oblanceolate, retuse, leaflet, prominently tuberculate beneath, rarely trifoliolate. Inflorescence lax, up to 9 cm long, gray-pilose in the apical branchlets. Pedicels densely pilosulous, with a pair of orange glands distally; calyx 3.5–4.5 mm long, densely gray-pilose, sub- spathaceous, the ribs with 1 row of 1–5 orange glands, the ovate teeth unequal, the ventral pair longest and broadest. Petals blue to blue-violet (rarely white), all almost always with apical glands.

**Representative examined material:** Coahuila: 13-X-1989, *J. Valdés R. 2009* (ANSM, TEX00273627); 23-VIII-1971, *D. Keil 8114* (OBI122750!); 18-X-1971, *W.R. Laverich 1215* (TEX00273636!).

**Distribution**: This is the only species of *Psorothamnus* present in northeastern Mexico. Outside the area, in southwestern USA (Arizona and New Mexico) and north of Mexico (Chihuahua). In desert scrublands, 1200–1400 m.

**Tribe Brongniartieae** (Benth.) Hutch., Gen. Fl. Pl. **1**: 393. 1964.

Herbaceous, subshrubs, shrubs or trees. Leaves pinnate, multifoliate, sometimes with glands. Stipules present, sometimes very large. Inflorescences in axillary racemes or panicles, rarely solitary. Calyx 5-toothed of 2-labiate. Stamens monadelphous or diadelphous. Anthers similar or alternately shorter. Style glabrous. Stigma terminal or oblique. Fruit 1-several seeds, dehiscent, sometimes explosively. Seeds surrounded by spongy tissue or those separated by transversal septa.

**Type:** *Brongniartia* Kunth, Nov. Gen. Sp. 6: 465. 1823.

The tribe includes 10 genera and approximately 152 species [31]. In our study area only two genera *Harpalyce* and *Brongniartia*, both with amphitropical disjunct distributions [38].
1A.Stamens diadelphous; calyx slightly bilabiate, 5-toothed, the upper two teeth united almost to the apex, the lower 3 free almost to the base; patelliform glands absent; low shrubs 1 m or shorter (in our area)***Brongniartia***1B.Stamens mondaelphous; calyx strongly bilabiate; the upper lip bidentate, the lower lip 3-toothed, the teeth inconspicuous; patelliform glands present; trees***Harpalyce***

***Brongniartia*** Kunth, Nov. Gen. Sp. 6: 465. 1823. *Megastegia* G.Don, Gen. Hist. 2: 468. 1832. *Peraltea* Kunth Nov. Gen. Sp. 6: 469. 1824.

**Type:** *Brongniartia mollis* Kunth, Nov. Gen. Sp. 6: 465. 1824.

Herbaceous, shrubs or trees. Stipules present, sometimes large. Leaves imparipinnate. Inflorescences axillary, in racemes, or few 1–3. Bracts present. Bracteoles large or tiny, at the base of the calyx, rarely in the form of a tuft of hairs. Calyx slightly bilabiate, the tube short, 5-toothed, the upper two teeth united almost to the apex, the lower 3 free almost to the base. Flowers papilionate, red, white, red-purple, yellow or brown. Banner orbicular. Wings oblong. Kell obtuse or incurved, all of them unguiculate basally. Stamen 10 diadelphous. Ovary short-stipitate. Ovules several. Style glabrate, filiform. Stigma terminal. Fruit short stipitate, oblong, oblong-obovate, coriaceous, with one of its margins narrowly winged. Seeds compressed, arranged transversely in the fruit, strophiolate.

Genus of ca. 63 species, and the 98% of those occurring in Mexico [1]. The species of this genus are distributed from west Texas to South America. Only two species are known from South America, and one species is present in the United States in Texas [38]. *Brongniartia* is mostly a Mexican genus, because most of the species are distributed within its geopolitical boundaries [39]. Most of the species are found in western Mexico, in tropical deciduous forests, and surrounding derived secondary vegetation. Just over a third of the recognized species are microendemic, recorded only in their type localities or adjacent areas. Thirteen species are endemic to southwestern Mexico, at the Rio Balsas Basin [40].
1A.Bractlets subtending the calyx glabrous21B.Bractlets subtending the calyx sericeous or pilose32A.Bractlets basally obtuse or rounded; leaflets densely pubescent when young***B. magnibracteata***2B.Bractlets basally cordate; leaflets glabrous***B. foliolosa***3A.Calyx pilose o sericeous***B. intermedia***3B.Calyx glabrous44A.Leaflets 5–17 mm wide***B. discolor***4B.Leaflets 2–2.5 mm wide***B. rozynskii***

***Brongniartia discolor*** Brandegee, Univ. Calif. Publ. Bot. 4: 272. 1912.

**Type:** Mexico, San Luis Potosi, VII-1911, *C.A. Purpus 5201* (Isotype: NY00006258!). Holotype not found.

**Distinguishing features:** Shrub 1–2 m tall. Young branches densely pubescent. Leaflets up to 25, 5–17 mm wide, long, oblong, bright green, thick, reticulate veined, basally cordate. Bracteoles pilose o sericeos. Calyx glabrous. Fruit glabrous.

**Representative examined material:** Nuevo León: 28-V-1970, *F. González Medrano*, *V. M. Toledo*, *E. Martínez S.*, *3007* (MEXU). Tamaulipas: 28-V-1986, *L. Hernández 1810* (CFNL); 20-VI-1986, *M. Martínez 1194* (CFNL); 23-V-1984, *A. Brito 245* (CFNL); 2-VI-1983, *L.J. Dorr*, *2654* (OBI117062!); 23-IX-1971, *F. González Medrano*, *E. Martínez S. 3723* (MEXU); 24-IX-1976, *F. González Medrano*, *P. Zavaleta*, *A. Sandoval 9977* (MEXU); 22-V-1977, *F. González Medrano; F. Guevara; P. Hiriart 10462* (MEXU); 20-V-1973, *M. C. Johnston*, *T. L. Wendt*, *F. Chiang 11145* (MEXU).

**Distribution:** Endemic to Mexico, from Tamaulipas, through Hidalgo and San Luis Potosí to Oaxaca. In desert scrublands, oak, and oak-pine forest, 1500–2000 m.

***Brongniartia foliolosa*** Benth. ex Hemsl., Diagn. Nov. Pl. p. 7. 1878.

**Type:** Mexico, [Hidalgo] Zimapan, non date, *T. Coulter 555* (Holotype: GH00059826!).

**Distinguishing features:** Shrub 0.5–3 m tall. Leaves 4–9 cm long. Leaflets 15–45, 4–10 mm long, sometimes strongly reticulated, coriaceous, glabrous. Flowers solitary axillary, or in fascicles of 2. Bractlets 0.7–1 cm long, basally cordate, glabrous. Corolla reddish to purple. Banner 1.3–1.8 cm long. Fruit long stipitate, the body 3–4 cm long, oblong, without winged margins. Seeds 4–6.

**Representative examined material:** Nuevo León: 22-IX-1993, *G.B. Hinton 23474* (MEXU); 21-VIII-1991, *Hinton 21267* (MEXU).

**Distribution:** Endemic to Mexico, from Nuevo León, Veracruz, Hidalgo, and San Luis Potosí to Puebla and Oaxaca. In oak-pine forest, 850–2000 m.

***Brongniartia intemedia*** Moric, Mém. Soc. Phys. Genève 7: 253. 1836.

**Type**: Mexico, 1834–40, *C. Ehrenberg n.n.* (Holotype: HAL-098426) [33].

**Distinguishing features:** Shrub, 0.5–1.5 m tall. Stems pubescent. Leaves up to 7–18 cm long. Leaflets 10–37 per leaf, ovate, up to 2.5 long, apically mucronate, densely pubescent when young, turning glabrate with age. Bract 1. Bractlets 2, obtuse or rounded basally, glabrate, located at the apex of the pedicel. Flowers papilionate mainly red, sometimes purple. Stamens 10, diadelphous. Fruit pendulous, stipitate, 4–7.5 cm long, wide-oblong, flattened, coriaceous, glabrate. Seeds 1 cm long, globose, brownish-red.

**Representative examined material:** Nuevo León: 22-IX-2001, *E. Estrada 13093*, *C. Yen* (CFNL); 15-IV-1988, *E. Estrada 1440* (CFNL); 6-VII-2003, *C. Yen*, *E Estrada 15681* (CFNL); 5-VII-1989, *E. Estrada 1576* (CFNL, TAES); 23-VII-2002, *C. Yen*, *E. Estrada 15070* (CFNL, TEX00438676); 29-IV-2007, *M. Barba 772*, *E Estrada* (CFNL). Tamaulipas: 19-VI-1995, *L. Gracía 006* (CFNL); 6-X-1982, *J. Henrickson 19075* (IBUG214001!).

**Distribution:** Endemic to Mexico. From Sinaloa, Nuevo León, and Tamaulipas, extending south through San Luis Potosí, Michoacán, and Guerrero. Frequently found in northeastern Mexico. In desert scrublands (mainly piedmont scrub) adjacent to oak forest (950 m), oak-pine forest, and conifer forest, 1000–2000 m.

***Brongniartia magnibracteata*** Schltdl., Linnaea 12: 338. 1838. Basionym: *Brongniartia obliqua* Schltdl., Linnaea 12: 339. 1838.

**Type:** Mexico, in Barranca prope S. Bartolo, 1834, *C.A. Ehrenberg 303* (Holotype: HAL0098425!).

**Distinguishing features:** Erect subshrub or shrub, 60–70 cm tall. Stems sericeous. Leaves 3–5.5 cm long. Leaflets 13–27 per leaf, 1.5–1.8 cm long, mucronate at apex, basally asymmetrical, not strongly reticulate, densely pubescent when young. Flower axillary, red, solitary, or in pairs. Bractlets subtending the calyx glabrous, basally obtuse, or rounded. Fruit 5–6 cm long, acute at both ends, coriaceous, with the upper suture marginal-winged, glabrous.

**Representative examined material:** Nuevo León: 26-III-1993, *Hinton* et al. *22735* (TEX-LL); 16-VI-1990, *Hinton* et al. *20369* (TEX-LL); 20-VI-1986, *E. Estrada 457* (CFNL, MEXU). Tamaulipas: 9-Xii-1976, *F. Guevara 10143* (UAT); 13-VII-1984, *P. Hiriat 248* et al. (UAT); 10-IX-1986, *M. Martínez 1295* (UAT); 20-VI-1986, *M. Martínez 1194* (UAT).

**Distribution:** Endemic to Mexico. From Nuevo León and Tamaulipas to Jalisco, Hidalgo, Veracruz, and Puebla. Common in oak-pine forests, desert scrublands, and rosetophyllous scrublands. In canyons, on the edges of gaps and roads, 90–1700 m.

***Brongniartia rozynskii*** Standl., Publ. Field Mus. Nat. Hist., Bot. Ser. 22: 23. 1940.

**Type:** Mexico, Tamaulipas, Jaumave, Sierra near San Lunar, VII-1932, *H.W. Von Rozynski 524* (Holotype?: P02950007!).

**Distinguishing features:** Shrub with short nodes. Leaves 6–8 cm long. Leaflets up to 21, 4–8 x 2–2.5 mm, oblong, base obtuse, apex rounded or acute, strigose in both surfaces, midvein, and lateral veins prominently reticulate in both surface, the margins revolute, Stipules caducous. Flowers solitary, axillary. Pedicel 1.5–2 cm long. Bracts 8 mm long, ovate, membranous, ciliate, rounded, or subchordate basally. Calyx 1 cm long, glabrous outside tube widely campanulate, the teeth longer than tube, lanceolate, atenuate, ciliate.

**Representative examined material:** Tamaulipas: VII-1932, *H.W. Von Rozynski 524* (P02950007!).

**Distribution:** Endemic to Mexico, apparently in desert scrubland.

***Harpalyce*** Moc. & Sessé ex DC., Mém. Légum.: 496. 1827.

Subshrubs, shrubs or trees. Leaves imparipinnate with conical, lepidote, globose, or patelliform multicellular glands, united on the surface of the vegetative structures. Leaflets opposite or sub-opposite on the leaf rachis. Inflorescences in racemes, panicles, or sub-corymbs. Flowers papilionate, white, white-greenish, dark purple, orange-red or white-pinkish, Stamens 10, monadelphous, the filaments of different size, alternately dimorphic, the upper ones with basifixed anthers, the lower ones with dorsifixed anthers. Ovary and style glabrous. Stigma terminal, small, penicillate. Ovules two. Fruit ovate or ovate-oblong, coriaceous, with or without septa, dehiscent. Seeds 1-several, laterally compressed round or square, glabrous or minutely pubescent.

Genus of almost 20 [38]-24 [1] species, from Mexico and West Indies to Brazil, mainly in tropical regions. Most species are endemic and have restricted distribution. Seven species and two varieties of *Harpalyce* are reported for Mexico [1], three of which reach distribution as far as northeast Mexico, exclusively in the state of Tamaulipas.
1A.Abaxial surface of the leaflets prominent and reticulate-veined***H. mexicana***1B.Abaxial surface of the leaflets not prominent and not reticulate-veined22A.Leaflets ovate to rounded-ovate, glabrate underside when mature, notched, or retuse apically***H. arborescens***2B.Leaflets oblong, densely sericeous underside, scarcely or not retuse apically***H. formosa*** var. ***formosa***

***Harpalyce arborescens*** A. Gray, Proc. Amer. Acad. Arts 5: 178. 1861. Basionym: *Brongniartia retusa* Benth., Diagn. Pl. Nov. Mexic.: 8. 1878. *Harpalyce hidalgensis* Taub. Bull. Herb. Boissier 3: 613. 1895. *Harpalyce retusa* (Benth.) Rose, Contr. U.S. Natl. Herb. 8: 43. 1903.

**Type:** Mexico, Huasteca: Wartenberg, near Tantoyuca, 1858, *L.C. Ervendberg*, *L.C.*, *18* (Holotype: PH00013614!).

**Distinguishing features:** Tree or shrub 3–8 m tall. Branches beige to reddish, tomentose color and mottled with yellow glands, covered with multiple white lenticels. Leaflets 9–11, 1.5–5 cm long, ovate or elliptic, abaxially covered with golden, biseriate, pateriform glands, veins not prominent abaxially. Inflorescences axillary, in racemes or panicles. Flowers papilionate, bicolored, pink to purplish-red, mixed with white or cream tones. Fruit 5–7 cm long, obovate, tick, coriaceous, reddish-brown, striate, rugose.

**Representative examined material**: Tamaulipas: 7-XII-1993, *A. Mora-Olivo 5056* (UAT); 15-XI-1985, *J. Jiménez 354* (UAT); 12-V-1982, *F. González Medrano*, *P. Hiriart 12472* (MEXU); 2-XII-19850, *L. Hernández 1620* (CFNL, MEXU, UAT); 7-V-1982, *A. Valiente Banuet*, *L. Hernández*, *F. González Medrano*, *P. Hiriart 31* (MEXU); 7-XI-1972, *J. Marroquín*, *H. Sánchez*, *G. Alanís 2378* (MEXU).

**Distribution**: Endemic to Mexico. In Tamauliupas, San Luis Potosí, Hidalgo, Querétaro, Guanajuato, and Yucatán. Limestones and basalt, tropical deciduous forest, oak, oak-pine forest, 100–1200 m.

***Harpalyce formosa*** var. ***formosa*** Mociño & Sessé ex DC., Prodr. 2: 523.1825. Basionym: *Harpalyce ferruginea* Brandegee, Zoe 5: 234. 1907. *Harpalyce loeseneriana* Taub., Bull. Herb. Boissier 3: 612. 1895.

**Type:** Mexico, Tehuacán, 1-VI-1905, *C.A. Purpus 1196* (Isotype: MO-128737!). Holotype not found.

**Distinguishing features:** Shrub up to 3 m tall. Branches with ferrugineous pubescence. Leaves up to 25 cm long. Leaflets 7–19, 1–5 cm long, ferrugineous abaxially. The veins not strongly reticulated abaxially. Inflorescences in racemes 7–12 cm long. Pedicels alternate. Flowers papilionate, pink, reddish-purple, or purple with dark red veins, green stained at the base. Fruit 2–2.5 cm wide, commonly tan-brown to reddish-brown.

**Representative examined material**: Tamaulipas: 19-VI-1985, *L. Hernández 1489* (CFNL); 27-VI-1971, *J.R. Sullivan 641* (TEX00562314!); 16-III-1994, *L. Hernández 3037* (MEXU).

**Distribution**: Endemic to Mexico, from Tamaulipas, State of Mexico, and Puebla extending south to Guerrero, Oaxaca, and Chiapas. In lower slopes in limestones, tropical deciduous forest, and desert scrublands (piedmont scrub), 700–1300 m.

***Harpalyce mexicana*** Rose, Contr. U.S. Natl. Herb. 8: 42. 1903.

**Type:** Mexico, Jalisco: West of Bolaños, 15/17-IX-1903, *Rose 2944* (Holotype: US00003856!).

**Distinguishing features:** Tree. Leaflets 11–15, 4–7 cm long, ovate to ovate-lanceolate, coriaceous, with orange, biseriate, pateriform, peltate glands, deeply embedded in the leaflets but evident to the naked eye, and strongly reticulated-veined abaxially (below). Inflorescences axillary, in few-flowered racemes. Fruit 5–6 cm long, oblong, apiculate, base obtuse, somewhat coriaceous, strongly rugose, internally partitioned, dark brown.

**Representative examined material**: Tamaulipas: 1-VI-1971, *J.R. Sullivan 664* (TEX00562346!).

**Distribution***:* Endemic to Mexico, mainly distributed in southern Mexico, from Tamaulipas and Veracruz to Guanajuato, Puebla, and Nayarit, extending far south to Guerrero and Oaxaca. Oak forest, 800–1200 m.

**Tribe Dalbergieae** DC., Prodr. 2:415. 1825, descr. emended B.B. Klitgaard & M. Lavin. In: Legumes of the World 2005. eds: Lewis, W.; Schrire, B.; Mackinder, B.; Lock, M. Dalbergieae sensu lato 307–335. Dalbergieae Bronn ex DC., Prodr. Syst. Nat. Reg. Veg. 2: 415. 1825. Tribe Aeschynomeneae (Benth.) Hutch., G.F.P. 1: 470. 1964.

**Type:** *Dalbergia* L.f. Suppl. Pl.: 52. 1782.

Trees, shrubs, or woody lianas. Sometimes glandular with pellucid punctate or with tuberculate-based hairs. Stipules sometimes appendiculate below point of attachment, sometimes spinescent. Leaves paripinnate or imparipinnate, 1–many-foliolate. Leaflets opposite to alternate. Inflorescences in racemes or panicles or the flowers solitary, axillary or terminal. Bracts similar to stipules or large and circular, often enclosing flowers and fruit. Corolla papilionate. Calyx campanulate with subequal lobes or teeth, or bilabiate, subtruncate to shortly toothed, the 2 upper teeth generally connate and higher than the other teeth, rarely the upper lobes large and wing-like. Stamen-filaments 9–10, monadelphous or diadelphous (5 + 5) or tube or the vexillary filament free or (*Dalbergia*) further subdivided. Anthers uniform or dimorphic, opening apically or longitudinally. Style glabrous or nearly so on upper part. Stigma small, terminal. Fruit flattened or drupe-like, indehiscent or a loment or lomentaceous (except *Arachis*, unjointed and geocarpic), straight or slightly curved, or sometimes coiled or plicate, enclosed in calyx.

Phylogenetic studies include 49 genera and around 1300 species within this tribe, distributed mainly in the neotropics [32]. In northeastern Mexico, there are nine genera, *Aeschynomene*, *Amicia*, *Arachis*, *Ctenodon*, *Dalbergia*, *Diphysa*, *Nissolia*, *Stylonsanthes*, and *Zornia*.
1A.Anthers erect, opening apically by horizontal slits or anthers divergent and opening by longitudinal slits; flowers white***Dalbergia***1B.Anthers parallel, opening by longitudinal slits; flowers yellow or yellow-orange22A.Fruits inflated bladder-like, pericarp papery; pedicels articulated ***Diphysa***2B.Fruits flattened or cylindrical, constricted regularly in the interseminal spaces, not inflated like a bladder, pericarp not papyraceous33A.Fruit not articulated, subcylindrical, developing below ground***Arachis***3B.Fruit articulated, or if subcylindrical, tetragonal in appearance, developing above ground44A.Leaves paripinnate, leaflets 2 or 454B.Leaves imparipinnate with 3, 5, 7 or more leaflets, 65A.Flowers sessile or subsessile; stipules foliaceous, ovate, acute apically; bracts ovate, enclosing or covering the flowers***Zornia***5B.Flowers pedicellate; stipules large, orbicular, obtuse, or emarginate apically; bracts orbiculate, obtuse, or emarginate, without enclosing or covering the flowers***Amicia***6A.Fruit with 1–2 articles, immersed in the calyx or slightly exceeding it; flowers yellow or yellow-orange; stipules fused to the petiole, frequently sheathing the stem***Stylosanthes***6B.Fruits with 3-more articles, significantly exceeding the calyx 77A.Distal article of fruit with a papery wing; plants scandent; leaflets 5–7***Nissolia***7B.Distal article of fruit without a wing; plants erect or suberect; leaflets more than 7**8**8A.Calyx with 5 subequal teeth; stipules without lateral appendages, not peltate***Ctenodon***8B.Calyx bilabiate, a lip bidentate, the other one tridentate; stipules peltate, appendiculate below the point of attachment***Aeschynomene***

***Aeschynomene*** L. Sp. Pl. 713. 1753, Gen. Pl. ed. 5 319. 1754. *Aedemone* Kotschy, Oesterr. Bot. Z. 8: 116. 1858. *Bakerophyton* (J. Léonard) Hutch., Gen. Fl. Pl. 1: 474. (1964. *Balisaea* Taub., Bot. Jahrb. Syst. 21: 436. 1896. *Climacorachis* Hemsl. & Rose, Contr. U. S. Nat. Herb. 8: 43. 1903. *Cajati* Rumph. ex Adans. Fam 2: 238. 1763. *Herminiera* Guill. & Perr., Fl. Seneg. Tent.: 201. 1832. *Macromiscus* Turcz. Bull. Oc. Nat. Mosc. 19(2): 507. 1846. *Mantodda* Adans., Fam. Pl. 2: 508. 1763. *Rochea* Scop. Intr. Hist. Nat.: 296. 1777. nom. rej. *Rueppelia* A. Rich., Tent. Fl. Abyss. 1: 203. 1848. *Secula* Samll, Fl. Miami 90: 200. 1913. *Segurola* Larrañaga, Escritos D. A. Larrañaga, Atlas 1: t. 93. 1927.

**Type:** *Aeschynomene indica* L. Sp. Pl.: 713. 1753.

Herbaceous or sub-shrubs (in our area). Leaves pinnate, commonly paripinnate. Stipules peltate and appendiculate basally near the point of insertion. Leaflets frequently numerous. Inflorescences usually axillary, in racemes or panicles. Bracts commonly similar to the stipules. Flowers papilionate, yellowish, almost white to yellow-orange. Calyx bilabiate, a lip bidentate, the other one tridentate. Stamens 10, the filaments united to form a sheath, forming 2 groups of 5. Ovaries frequently stipitate. Ovules 2-many. Fruit a loment, with 1–18 articles. Seeds reniform, smooth, and shiny.

Genus of approximately 175–180 species, although with higher estimates reaching up to 250 species [1]. Recent molecular biology studies [41] have divided *Aeschynomene* into two genera, adding *Ctenodon*, which comprises 66 species in America, but possibly reaches up to 120 species on the planet given that the African species have not yet been studied using molecular phylogenetic methods [41]. Of the approximately 80 species of *Aeschynomene* recognized by [1] for Mexico and Central America, half of them are now included within *Ctenodon*, such that *Aeschynomene* may now be represented by at least 40–50 species in this area. We recorded three species for northeastern Mexico.
1A.Leaflets 1-costate***A. scabra***1B.Leaflets 2-several costate22A.Ovary and fruit glabrous; fruit articulated between seeds, its margins reticulate-veined***A. americana*** var. ***americana***
2B.Ovary villose, with stipitate glands 1 mm long, its bases dark; not evidently or weak articulated between seeds, not reticulated-veined ***A. villosa*** var. ***villosa***

***Aeschynomene americana*** L., Sp. Pl. 713. 1753. var. ***americana*.** Basionym: *Aeschynomene americana* var. *depila*, Millsp. Field. Mus. Publ. Bot. 1: 363. 1898. *Aeschynomene americana* var. *glandulosa* (Poir.) Rudd, Contr. U.S. Natl. Herb. 32: 26. 1955. *Aeschynomene divisa* Nees & Mart., Nova Acta Phys.-Med. Acad. Caes. Leop.-Carol. Nat. Cur. 12: 31. 1826. *Aeschynomene guayaquilensis* G.Don, Gen. Hist. 2: 284. 1832. *Aeschynomene javanica* var. *luxurians* Miq., Fl. Ned. Ind. 1(1): 276. 1855. *Aeschynomene mexicana* Biroli ex Colla, Herb. Pedem. 2: 195. 1834. *Aeschynomene mimulosa* Blume ex Miq., Fl. Ned. Ind. 1(1): 276. 1855. *Aeschynomene tricholoma* Standl. & Steyerm., Publ. Field Mus. Nat. Hist., Bot. Ser. 23: 10. 1943. *Aeschynomene versicolor* Wender., Bot. Zeitung (Berlin) 1: 347. 1843. *Colutea aeschinomenoides* Scop., Delic. Fl. Faun. Insubr. 3: 22. 1788. *Hedysarum acayucense* Sessé & Moc. Fl. Mexic., ed. 2: 171. 1894. *Hippocrepis mimulosa* Noronha, Verh. Batav. Genootsch. Kunsten 5(4): 18. 1790. nom. inval.

**Type**: H. Sloane, Voy. Jamaica 1: 186, t. 118, f. 3. 1707. Lectotype designed by Howard, Fl. Lesser Antilles 4: 443. 1988. Herb. Sloane 3: 90 (BM000589674!).

**Distinguishing features:** Herbaceous, up to 1.5 m tall. Leaves 2–7 cm long. Leaflets 20–60, 2-several costate. Inflorescences in short in few-flowered racemes, those as long as the subtending leaves. Flowers 6–8 mm long. Calyx bilabiate, one lip didentate, the other one tridentate. Ovary and fruit glabrous. Fruit 3–9 articulate, articulated between seeds, frequently muricate at the center of the articles, glabrate to puberulent, sometimes with sparse glandular trichomes, or if there is pubescence or glandular trichomes present, this is slight and localized on the surface or margins.

**Representative examined material:** Tamaulipas: 3-II-2000, *A. Mora-Olivo 7864* (UAT).

**Distribution:** Widely distributed in America, from Florida (USA), throughout Mexico and Caribbean islands, extending to Venezuela and Brazil. In northeastern Mexico inhabiting low deciduous forest, 300–450 m.

***Aeschynomene scabra*** G. Don, Gen. Hist. 2: 284. 1832.

**Type:** Ecuador, Guayas, Native of Guayaquil, 1778, *H. Ruíz L. & J. A. Pavón n.n.* (Presumably Holotype: F0058819F!. According to Rudd [42], the type species label says “Native of Guayaquil”, and collected presumably by Ruiz and Pavón. Although the author does not detail or indicate where she reviewed the Ruiz & Pavón material, she does mention [42] that the sample consists of two separate branches, one with flowers and fruits and another with fruits, which corresponds to the sample stored in the Field Museum of Natural History, Chicago (F0058819).

**Distinguishing features:** Herbaceous to sub-shrubby, up to 2 m tall. Leaves 5–12 cm long. Leaflets 30–55, 0.5–1.5 cm long, 1-costate. Flowers yellow, 8–11 mm long. Calyx 6–7 mm long. Fruit stipitate, the stipe 0.5–1.5 cm long, the body with 10–14 articles, the upper margin entire, the lower one subentire or crenate, hispidulose, rarely glabrate, verrucose to muricate at central region.

**Representative examined material:** Tamaulipas: 9-IX-1994. *Arturo Mora Olivo*, *Jorge L. Mora López 05495* (MEXU).

**Distribution:** Widely distributed from north of Mexico and Central America to Brazil. In the study area at permanent puddles, aquatic vegetation, sandy-clayish soils, 30 m.

***Aeschynomene villosa*** Poir. Encycl. Suppl. 4: 76. 1816. var. ***villosa*.** Basionym: *Adesmia fruticulosa* G. Don, Gen. Hist. 1: 282. 1831. *Aeschynomene americana* var. *longifolia* Micheli, Bot. Gaz. 20: 284. 1895. *Aeschynomene decumbens* Zipp. ex Span., Linnaea 15: 193. 1841. *Aeschynomene floribunda* M.Martens & Galeotti, Bull. Acad. Roy. Sci. Bruxelles 10(2): 186. 1843. *Aeschynomene fruticulosa* Nees ex G. Don, Gen. Hist. 2: 282. 1832. *Aeschynomene glandulosa* Benth. Pl. Jungh.: 210 (1852) nom. illeg. *Aeschynomene hirsuta* DC., Prodr. 2: 322. 1825. nom. illeg. *Aeschynomene javanica* Miq., Fl. Ned. Ind. 1(1): 275. 1855. *Aeschynomene pseudoviscosa* Blume ex Miq., Fl. Ned. Ind. 1(1): 276. 1855. *Aeschynomene pudica* Zoll. & Moritzi Natuur- Geneesk. Arch. Ned.-Indië 3: 55. 1846. nom. inval. *Aeschynomene timoriana* Span. Icon.: 62. 1791. *Aeschynomene villosa* var. *longifolia* (Micheli) Rudd, Contr. U.S. Natl. Herb. 32: 35. 1955. *Cassia tenuicaulis* M.E.Jones, Contr. W. Bot. 18: 40. 1933.

**Type**: Guatemala, Santa Rosa de Casillas, X-1892, E.T. Heyde, E. Lux 4172 (Lectotype: US00001869!).

**Distinguishing features:** Herbaceous, up to 65 cm tall. Stems frequently decumbent. Leaflets 5 mm long or less, 3-costate. Inflorescences in few-flowered racemes, longer than subtending leaves. Flowers yellow, 3–7 mm long. Ovary villous. Fruits commonly articulated, its surface lacking conspicuous venation or murication, hispid with yellow glandular trichomes 1 mm long, their bases, tuberculate and dark.

**Representative examined material:** Nuevo León: 26-VI-2004, *E. Estrada* et al. *16237* (CFNL); 9-XI-2001, *C. Yen y E. Estrada 13189* (CFNL); 6-X-2000, *C. Yen*, *E Estrada 13145* (CFNL). Tamaulipas: 9-XI-1994, *Hinton* et al., *25049* (ARIZ325448!); 11-XI-1975, *A. Lasseinge n.n.* (MEXU); 27-IX-1959, *J. Graham. M.C. Johnston 4078* (MEXU).

**Distribution:** Widely distributed, from southern USA (Arizona), throughout Mexico, Central America, and Venezuela to Colombia, and Ecuador. This variety includes *A. villosa* var. *longifolia* (Micheli) Rudd., previously recognized as a different variety. Desert scrublands (piedmont scrub), deciduous forest, 750–950 m.

***Ctenodon*** Baill., Adansonia 9: 236. 1870. Basionym: *Balisaea* Taub., Bot. Jahrb. Syst. 21: 436. 1895. *Secula* Small, Fl. Miami [Small] 90: 200. 1913. *Aeschynomene* sect. *Ochopodium* Vogel, Linnaea 12: 86. 1838. *Aeschynomene* subgen. *Ochopodium* (Vogel) J. Léonard, Bull. Jard. Bot. État. Brux. 24: 84. 1954.

**Type***: Ctenodon weddellianus* Baill., Adansonia 9: 237. 1870.

Herbaceous or shrubby (in our area). Leaves pinnate, commonly paripinnate. Stipules attached at the base, non-peltate. Leaflets frequently numerous. Inflorescences usually axillary, in racemes or panicles. Flowers papilionate, yellowish, almost white to to yellow-orange. Calyx campanulate, 5-dentate with subequal teeth. Stamens 10, monadelphous or diadelphous. Ovary frequently stipitate. Ovules 2-many. Fruit, an articulate loments, 1–18 articled. Seeds reniform, smooth, and shiny. Genus of approximately 78 recognized and accepted species (Fernando Tapia-Pastrana, first karyotypic register of *Ctenodon elegans* (Leguminosae: Papilionoideae: Dalbergieae).

A recent molecular phylogenetics [41], proposed that all species previously included within *Aeschynomene* sect. *Ochopodium* [42] were moved to the reinstated genus *Ctenodon* Baill. [41]. *Ctenodon* as genus can be differentiated from *Aeschynomene* (sect. *Aeschynomene*) by having non-peltate, basifixed stipules, calyx campanulate, 5-dentate with subequal teeth, and articulate loments [42,43]. In northeastern Mexico, only one species was recorded.

***Ctenodon fascicularis*** (Schltdl. & Cham.) A. Delgado, Neodiversity 13: 18. 2020. Basionym: *Adesmia mimosoides* G. Don, Gen. Hist. 2: 282. 1832. *Aeschynomene fascicularis* Schltdl. & Cham., Linnaea 5: 584. 1830. *Aeschynomene fruticosa* Sessé & Moc., Naturaleza (Mexico City), ser. 2, 1(App.): 122. 1890. *Aeschynomene mimosoides* Nees ex G.Don, Gen. Hist. 2: 282. 1832. *Aeschynomene oligantha* Micheli, Mém. Soc. Phys. Genève 34: 256. 1903. nom. illeg.

**Type:** MEXICO, Veracruz, “inter la Laguna Verde et Actopan”, III-1829, C.J.W. Schiede & F. Deppe *n.n.* HAL0098430!. Lectotype Designated by Cardoso, D. B. O. S., C. M. J. Mattos, F. Filardi, A. Delgado Salinas, M. Lavin, P. L. R. de Moraes, F. Tapia-Pastrana & H. C. Lima: *Neodiversity* 13: 18. 2020.

**Distinguishing features:** Shrub 1–2 m tall. Stipules basifixed, 5–8 mm long. Leaflets 40–50, 1–2 cm long, oblong. 1-costate, it excentric, but not marginal. Inflorescences short, axillary, in racemes shorter than subtending leaf. Flowers yellow-orange, turning chocolate with age, 0.8–1.5 cm long. Calyx 4–5 mm long. Stamens 8–9 mm long. Fruit stipitate. Stipe 3–4 mm long, the articles 3–5, 6–8 × 45 mm, reticulate. Seeds up to 5 mm long, brown.

**Representative examined material:** Tamaulipas: 31-VIII-1984, *L. Hernández 1178* (MEXU); 8-XI-1996, *C. Ramos 151* (MEXU); border Tamaulipas-San Luis Potosi: 16-XI-1959, *M. Johnston*, *J. Graham 4727* (TEX00261190).

**Distribution:** Widely distributed, from northern Mexico throughout Central America, and Venezuela. In northeastern Mexico and adjacent to the geopolitical border with the state of San Luis Potosí, recorded in low deciduous forest, 250 m.

***Amicia*** Kunth, Nov. Gen. et Sp. 6: 511. 1823. *Zygomeris* DC., Prodr. 2: 315. 1825. nom. inval.

**Type:** *Amicia glandulosa* Kunth, Nov. Gen. Sp. 6: 511. 1824.

Plants herbaceous or shrubby. Stipules foliaceous, broad, orbicular or reniform. Leaflets 4, heart-shaped leaflets facing downwards. Glands sessile, round, present in stems, leaves, and flowers. Inflorescences in axillary or terminal racemes. Bracts broad, orbicular, obtuse, or reniform provided with small glands. Flowers papilionate, yellow, large, and showy or small. Calyx deeply divided, campanulate, 5-toothed, the teeth equal to or longer than the tube, the 2 upper ones wide and large, the lateral ones the smaller, the lower one narrow and long. Stamens 10, diadelphous, anthers uniform, elliptic and dorsifixed, style straight, apically short hooked. Fruit a loment laterally compressed with 2 to several indehiscent articles, separating from each other at maturity.

Genus with approximately 7 species, distributed in tropical and subtropical areas of Mexico (1 species) and South America [1,44].

***Amicia zygomeris*** DC., DC., Prodr. 2: 315. 1825. Basionym: *Hedysarum grandiflorum* Sessé & Moc., Naturaleza (Mexico City), ser. 2, 1(App.): 123. 1890. nom. illeg. *Zygomeris flava DC.*, Prodr. 2: 315. 1825. nom. inval.

**Type:** Mexico, Pátzcuaro, Michoacán, in Patzquari montibus, floret Augusto et Septembri (lectotype Plate Number 0523 in Torner Collection (photo!), Hunt Institute for Botanical Documentation, Carnegie Mellon University) (Särkinen & Hughes, 2015).

**Distinguishing features:** Herbaceous 1–2.5 m tall, stems erect, striate, hollow. Stipules orbicular, reniform, up to 2 cm long. Leaves paripinnate, 8–12 cm long. Leaflets 4, 4–5.8 cm long, obovate or cordate. Inflorescences in racemes, 4–12 cm long, almost double when the fruit ripens. Bracts 10–13 mm long, orbicular, foliaceous, papery, greenish, reticulate. Flowers 2–2.5 cm long. Calyx with transparent glands, strongly reticulate. Corolla up to 2.2 cm long. Stamens 10, diadelphous or monadelphous, filaments dimorphic, up to 2.9 cm long. Ovary minute, slightly constricted between the ovules, style filiform, ovules 2–5. Fruit a loment, up to 2.5 cm long, cylindrical, torulose, constricted between the interseminal spaces, with 1–3 articles, each article 12 × 6–7 mm, laterally flattened, glabrous, brown, soon deciduous. Seeds 3–4, kidney-shaped, light brown.

**Representative examined material:** Nuevo León: 26-VIII-1936, *M. Taylor 236* (TEX-LL); 2-VI-1934, *M.T. Mueller 691* (TEX-LL); 10-XI-2001, *C. Yen y E. Estrada 13233* (CFNL); 21-VIII-1991, *Hinton* et al. *21233* (TEX-LL); 22-IX-2001, *C. Yen y E. Estrada 13082* (CFNL); 4-VII-1933, M.T. Mueller 495 (TEX-LL). Tamaulipas: 7-X-1987, *M. Martínez 1589* (UAT); 15-X-1989, *F. González Medrano*, *V. Juárez*, *M. Hernández*, *H. González 17282* (MEXU); 2-X-1973, *F. González Medrano*, *R. M. López*, *R. Hernández 6369* (MEXU); 25-V-1970, *F. González Medrano*, *V. M. Toledo*, *E. Martínez S. 2939* (MEXU); 29-VIII-1968, *A.T. Richardson 897* (TEX00191880); 1-III-1968, *Warburton 32* (TEX00562115).

**Distribution:** Endemic to Mexico, widely distributed, from Sonora to Tamaulipas, but absent in the arid areas of Chihuahua and Coahuila within the Chihuahuan Desert region. Extending to the south, in Veracruz, Guerrero, Oaxaca to Chiapas. Species easily distinguished by its large orbicular, obtuse, or emarginate apically stipules, and its orbiculate, obtuse, or emarginate bracts, coupled with the leaves with only 2 pairs of large, cordate leaflets. Common, in humid and shaded areas of ravines and canyons, associated with oak and oak-pine forests, 800–1700 m.

***Arachis*** L, Sp. Pl. 741. 1753. *Arachidna* Plum. ex Boehm., Defin. Gen. Pl.: 255 (1760) nom. illeg. *Mundubi* Adans., Fam. Pl. 2: 323. 1763.

**Type:** *arachis hypogaea* L., Sp. Pl.: 741. 1753.

Herbs, annual, biennal, or perennial, sometimes rhizomatous or stolonifeorus. Stipules large, partly adnate to the petiole. Leaves paripinnate. Leaflets opposite, subsessile, almost always tetrafoliate. Inflorescences axillary, frequently solitary flower or a few-flowered cluster. Calyx membranous, bilabiate, upper lip wider, 4-toothed, lower lip falcate. Corolla papilionate, yellow or orange. Stamens monadelphous, 10, usually one of them absent. Anthers dimorphic, alternate long and short ones, the largest almost dorsifixed, the short ones basifixed. Ovary subsessile, basally elongated, and curved after fertilization. Ovules 2–6. Style filiform. Stigma terminal. Fruit oblong, subtorulose, its walls thick, smooth, reticulated. Seeds 1–6, indehiscent.

Genus with 69 accepted species, native to South America, most of species are found in Brazil [1]. In northeastern Mexico, only one species recorded, cultivated, *Arachis hypogaea* (cacahuate, peanut). Species widely used as food and source of vegetal oil and forage.

***Arachis hypogaea*** L. Sp. Pl.: 741. 1753. Basionym: *Arachidna hypogaea* (L.) Moench, Methodus: 122. 1794. *Arachidna quadrifolia* Trew, Pl. Rar. Hort. Domest.: t. 3. 1764. nom. inval. *Arachis africana* Lour., Fl. Cochinch.: 430. 1790. nom. illeg. *Arachis asiatica* Lour., Fl. Cochinch.: 43. 1790. *Arachis guaraniana* Bertoni, Catalogo espec. o var. plant. cult. Estac. Agron. Pto. Bertoni: 6. 1912. *Arachis hypogaea* f. *nambyquarae* (Hoehne) F.J. Herm., Agric. Monogr. U.S.D.A. 19: 14. 1954. *Arachis hypogaea* f. *typica* Hoehne, Fl. Brasílica 25(2): 18. 1940. nom. inval. *Arachis hypogaea* subsp. *fastigiata* Waldron, Contr. Bot. Lab. Morris Arbor. Univ. Pennsylvania 4: 312. 1919. *Arachis hypogaea* subsp. *nambyquarae* (Hoehne) A. Chev., Rev. Bot. Appl. Agric. Trop. 13: 772. 1933. *Arachis hypogaea* subsp. *procumbens* Waldron, Contr. Bot. Lab. Morris Arbor. Univ. Pennsylvania 4: 312. 1919. *Arachis hypogaea* var. *aequatoriana* Krapov. & W.C. Greg., Bonplandia (Corrientes) 8: 154. 1994. *Arachis hypogaea* var. *hirsuta* Köhler, Med.-Pfl., ed. 3: 42. 1898. *Arachis hypogaea* var. *nambyquarae* (Hoehne) Burkart, Darwiniana 3: 281. 1939. *Arachis hypogaea* var. *peruviana* Krapov. & W.C. Greg., Bonplandia (Corrientes) 8: 153. 1994. *Arachis hypogaea* var. *vulgaris* Harz, Landw. Samenk.: 642. 1885. *Arachis nambyquarae* Hoehne, Relat. Commiss. Linhas Telegr. Estratég. Matto Grosso Amazonas 5(12): 21. 1922. *Arachis oleifera* A. Chev., Rev. Bot. Appl. Agric. Trop. 13: 770. 1933. *Arachis rasteiro* A. Chev., Rev. Bot. Appl. Agric. Trop. 9: 487. 1929. nom. inval.

**Type:** Netherlands, *G. Clifford n.n.* (Lectotype: BM000646534!).

**Distinguishing features:** Herbaceous, annual, 30–50 cm tall. Leaves paripinnate. Leaflets 4, 1–7 cm long, elliptic, elliptic-obovate to obovate. Inflorescences axillary, in racemes. Flowers 1–1.5 cm long, yellow, yellowish-orange, red-veined. Fruit 3–7 cm long, indehiscent, sub-torulose, its walls thick, reticulate. Seeds 1–6. The fruit is geocarpic, developing underground, after fertilization, the gynophore, as it lengthens, forms an elongated thread-like structure, which goes into the ground, allowing the fruit to develop underground.

**Representative examined material:** Nuevo León: 26-IX-2024, *E. Estrada 26643* (CFNL). Tamaulipas: 22-VII-1989, *J.L. Mora-López 25* (UAT).

**Distribution:** Cultivated in Nuevo León, mainly used as food.

***Dalbergia*** L. f. Suppl. Pl. Syst. Veg. 57.1781. *Acouroa* Aubl., Hist. Pl. Guiane 2: 753 1775. nom. rej. *Amerimnon* Jacq., Enum. Pl. Carib. 27. 1760. *Coroya* Pierre, Fl. Forest. Cochinch.: t. 392 C. 1899. *Drakensteinia* DC., Prodr. 2: 476. 1825. *Ecastaphyllum* P. Browne, Civ. Nat. Hist. Jamaica: 299. 1756. *Endespermum* Blume, Catalogus: 24. 1823. *Fornasinia* Bertol., Misc. Bot. 8: 18. 1849. *Hecastophyllum* Kunth, Nov. Gen. Sp. 6: 387. 1824. *Miscolobium* Vogel, Linnaea 11: 200. 1837. *Miscolobium* Vogel, Linnaea 11: 200. 1837. *Podiopetalum* Hochst., Flora 24: 657. 1841. *Pterocarpus* P.J. Bergius, Kongl. Vetensk. Acad. Handl. 30: 116. 1769. nom. illeg. *Securidaca* L., Sp. Pl.: 707. 1753 nom. rej. *Semeionotis* Schott, Wiener Z. Kunst 3: 804. 1829. *Trioptolemea* Mart. ex Benth., Comm. Legum. Gen.: 38. 1837. *Triptolemea* Mart., Flora 20(2 Beibl.): 122. 1837.

**Type**: *Dalbergia lanceolaria* L.f. Suppl. Pl.: 316. 1782.

Trees, shrubs, or lianas. Leaves imparipinnate. Leaflets 1–many, alternate on rachis. Inflorescences axillary or terminal racemes or panicles. Bractlets paired at the base of calyx. Calyx campanulate, the teeth equal or different in size, the carinal, the longest. Corolla papilionate, small. Petals subequal, unguiculate, the keel petals adherent along the lower margin, purple, yellow-orange to white. Stamens 10, or in two fascicles of 5. Anthers small, erect, opening apically by horizontal slits or anthers divergent and opening by longitudinal slits. Ovary stipitate. Style subulate. Stigma capitate or indeterminate. Fruit stipitate, sometimes constricted in the middle, oblong, elliptic to orbicular, falcate-reniform to lunate-shaped, frequently flattened and indehiscent, wingless or with the wing surrounding the seminiferous area, Seeds 1–2, reniform, flat, brown.

Genus with approximately 250 species [1] from tropical and subtropical America, Asia, and Africa. In Mexico, 20 [45]–27 [46] species of *Dalbergia* are recorded. In northeastern Mexico only one species is found, *Dalbergia brownei*, in the state of Tamalipas.

***Dalbergia brownei*** (Jacq.) Urb., Symb. Antill. (Urban). 4(2): 295, in obs. 1905. Basionym: *Amerimnon brownei* Jacq., Enum. Syst. Pl. 7, 27. 1760.

**Type:** Jamaica, *nondate*, *P. Browne n.n.* (Holotype: BM000931833!).

**Distinguishing features:** Shrub up to 4.5 m tall. Branches decumbent, reclining: Leaves unifoliolate, 3.5–7 cm long, obtuse to acute, glabrous, shiny. Inflorescences in clusters. Flowers white.

**Representative examined material:** Tamaulipas: 10-VI-1997, *A. Mora-Olivo 7297* (MEXU, UAT); 30-IV-1898, *C.G. Pringle 6809* (MEXU).

**Distribution:** Widely distributed, from Tamaulipas, Veracruz to Oaxaca, extending south to West Indies, Central America to South America. In Mexico, this is the only species with unifoliolate leaves. Deciduous forest 200–350 m.

***Diphysa*** Jacq., Enum. Syst. Pl.: 7. 1760.

Trees or shrubs, unarmed, with spinescent branches. Viscid glands sometimes present. Leaves alternate, imparipinnate. Leaflets opposite, subopposite, or alternate, along rachis. Inflorescence in short axillary racemes or fascicles. Pedicels articulated below the calyx, each 2-bracteolate. Flowers yellow, papilionate. Calyx campanulate, 5-toothed, the teeth unequal, the 2 uppermost ones relatively broad, the 2 lateral ones equal to these in length but narrower, the lowest one narrow, acute, longer than the others. The banner petals the longest, the keel petals the shortest, all petals short short-clawed. Stamens 10, diadelphous. Anthers uniform. Ovary stipitate. Style glabrous. Stigma small, terminal. Ovules many. Fruit stipitate, the body inflated, bladder-like or somewhat flattened, oblong, compressed.

Genus of 15 species distributed from Mexico to Central America, one species in south USA and another in South America [1]. Only two species recorded in northeastern Mexico.
1A.Small shrub 1–2 m tall or less; leaves 2–4 cm long; fruit papyraceous, its vexilar margin sulcate ***D. microphylla***1B.Trees 3–15 m tall or more; leaves 6–17 cm long; fruit somewhat coriaceous, its vexilar margin non sulcate ***D. americana***

***Diphysa americana*** (Mill.) M. Sousa, Ann. Missouri Bot. Gard. 77(3): 576. 1990. Basionym: *Colutea americana* Mill., Gard. Dict. ed. 8. *Colutea* No. 5, 1768. *Diphysa robinioides* Benth., in Benth. et Oerst., Vidensk. Meddel. Dansk Naturhist. Foren. Kjövenhavn 1853 (1–2): 11, 12. 1854. *Diphysa carthaginensis* sensu Benth., in Benth. et Oerst., Vidensk. Meddel. Dansk Naturhist. Foren. Kjövenhavn 1853 (1–2): 13. 1854, non Jacq. (1760).

**Type:** Mexico, Veracruz, 1731, *W. Houstoun n.n.* (Holotype: M000931458!).

**Distinguishing features:** Tree 3–8 (in our area)-15 m tall. Branches without brachiblasts, unarmed. Leaves 6–17 cm long. Leaflets 7–21, 1.5–4.2 cm long. Inflorescences in axillary racemes, 2.5–6.5 cm long. Fruit 4.5–13 × 1.3–2.5 cm, stipitate, the stipe covered by the calyx, the body flattened, barely inflated, somewhat coriaceous, constricted in some interseminal spaces, glabrate, reticulated, shiny. Seeds 7–10 × 3–4 mm, elliptic, light brown.

**Representative examined material:** Tamaulipas: 4-X-2008. *E. Martínez 40651* (MEXU 1281397); 4-X-2008, *E. Martínez 40651* (MEXU 1281390).

**Distribution**: Mexico and Mesoamerica, from San Luis Potosí and Querétaro to Chiapas, along and between the Gulf and Pacific coasts. Easily distinguishable from *D. microphylla* due to its size, large shrub or tree, larger than 3 m tall, leaves longer than 6 cm, and its relatively flattened and somewhat leathery fruit. Deciduous forest, 450 m.

***Diphysa microphylla*** Rydb., in Britton N. Amer. Fl. 24(4): 213. 1924. Basionym: *Diphya minutifolia* sensu Standley, *pro parte.* Contr. U.S. Nat. Herb. 23(2): 478–479. 1922, *non-Rose* (1909).

**Type:** Mexico, Tamaulipas, vicinity of Victoria, *E. Palmer 367* (Holotype NY00007757!).

**Distinguishing features:** Shrub, 0.5–2 m tall. Branchlets with brachyblasts, commonly spinescent. Leaves 2–4 cm long. Leaflets 7–19, 3–7 × 1.7–2.5 mm, papillate abaxially, punctate. Inflorescences 2–2.5 cm long. In short, axillary racemes with 2–4 flowers. Pedicels basally articulate. Fruits inflated, stipitate, the stipecovered by the calyx, papyraceous, barely reticulate, vexilar margin sulcate.

**Representative examined material:** Nuevo León: 16-IV-2001, *C. Yen y E. Estrada 12430* (CFNL). 7-VII-2001, *C. Yen y E. Estrada 13025* CFNL); 15-XI-1990, *E. Estrada 1923* (CFNL); 10-VII-2002, *C. Yen y E. Estrada 14770* (CFNL); 13-VII-2002, *C. Yen y E. Estrada 14785* (MEXU); 23-VII-2002, *C. Yen y E. Estrada 15005* (MEXU); 24-VIII-2003, *C. Yen y E. Estrada 15900* (MEXU). Tamaulipas: 7-X-1999, *A. Mora-Olivo 7644* (UAT); 22-IX-1999, *A. Mora-Olivo 7619* (UAT).

**Distribution**: Endemic to Mexico. Outside the area, in San Luis Potosí and Veracruz. In desert scrublands (Tamaulipan thornscrub), low deciduous forest, 70–530 m.

***Nissolia*** Mill., Gard. Dict. Abr., ed. 4.: [s.p.]. 1754. *Pseudomachaerium* Hassler, Bull. Herb. Boiss. II, 7: I, 1907.

**Type:** *Nissolia fruticosa* Vell., Fl. Flumin.: 298. 1829.

Vines or postrate (one species), herbaceous or somewhat woody. Leaves imparipinnate. Leaflets 5–7. Stipules lanceolate to ovate. Inflorescences axillary, in racemes or rarely in panicles. Flowers papilionate. Calyx campanulate 5-toothed, the teeth subequal. Petals commonly yellow, at times white or purple. Stamens 10, diadelphous- Fruit a loment samara-like, 2–5-articulate, the distal one sterile, winged. Seeds reniform, compressed, sublustrous, brown to reddish-brown.

American genus with 12 [47]–15 [48] species distributed from southern USA (Arizona and Texas) through Mexico, where the largest number of species are found, and extending to Argentina and Paraguay. In northeastern Mexico, within the tribe Dalbergieae, *Nissolia* is the only genus characterized by having a climbing habit and by having lomentaceous fruits, similar to a samara, with the distal article winged.
1A.Stipe of the fruit 3–6 mm long, longer than calyx length; inflorescence not fasciculated, its axis lengthening considerably at fruit stage ***N. fruticosa*** var. ***fruticosa***1B.Stipe of the fruit 1–4 mm long, shorter than calyx length; inflorescence commonly fasciculed, its axis not elongating at fruit stage22A.Prostrate plants; distal article of the fruit almost the same size and width as the fertile articles***N. wislizeni***2B.Climbing plants; distal article of the fruit wider and longer than the fertile articles33A.Fruit densely villose when young; fruit and frequently the calyx with abundant glandular setae***N. platycarpa***3B.Fruit no densely villous when young; fruit and calyx glabrous or pubescent, without glandular setae44A.Flowers 1.4–2 cm long; calyx tube 4.5–10 mm long***N. platycalyx***4B.Flowers up to 1.4 cm long; calyx tube up to 4 mm long55A.Stipules 1.5–3 mm wide at base***N. laxior***5B.Stipules 1 mm wide or less66A.Calyx tube 2–4 × 2–4 mm, its teeth 1–4 mm long***N. pringlei***6B.Calyx tube 1–2 × 1.5 mm, its teeth 0.5–2 mm long***N. leiogyne***

***Nissolia fruticosa*** Jacq., Enum. Syst. Pl.: 27. 1760. var. ***fruticosa.*** Basionym: *Machaerium berteronianum* (Steud.) Urb., Repert. Spec. Nov. Regni Veg. 17: 402. 1921. *Machaerium verapazense* Donn.Sm., Bot. Gaz. 40: 2. 1905. *Nissolia aculeata* Spreng., Syst. Veg., ed. 16. 3: 190. 1826. nom. illeg. *Nissolia berteroniana* Steud., Nomencl. Bot., ed. 2, 2: 196. 1841. *Nissolia costaricensis* Donn. Sm., Bot. Gaz. 44: 108. 1907. *Nissolia dubia* Poir., Encycl., Suppl. 4: 99. 1816. *Nissolia nelsonii* Rose, Contr. U.S. Natl. Herb. 5: 162. 1897. *Nissolia polysperma* Bertero ex DC., Prodr. 2: 257. 1825. *Nissolia racemosa* DC., Prodr. 2: 257. 1825.

**Type**: Colombia, [Bolivar], Cartagena, *Jacquin* (Killip negative 629 photo of the type ex BM) (Rudd, 1955), probably correponding to BM000931572! (Country unknown, *N.J. Jacquin n.n.*).

**Distinguishing features:** Liana, climbing. Inflorescence racemose with abundant flowers. Flowers 5–10 mm long. Calyx 2–4 mm long, its teeth 0.5–1 mm long.

**Representative examined material:** Tamaulipas: 12-X-2024, *A. Mora-Olivo n.n.* (UAT); 21-X-1983, *L. Hernández 801*, *F. González-Medrano*, *C. Cortés* (UAT); 27-IX-1996, *C. Ramos n.n.* (CFNL, UAT); 26-IX-1960, *M. Johnston 5736* et al. (MICH1179187).

**Distribution:** Species recorded for the first time in the northeastern Mexico, widely distributed from northern Mexico to Venezuela. Chaparral, deciduous forest, and sub-deciduous forest, 200–2000 m.

***Nissolia laxior*** (B.L. Rob.) Rose, Contr. U.S. Natl. Herb. 5: 162. 1897. Basionym: *Nissolia confertiflora* var. *laxior* B. L.Rob., Proc. Amer. Acad. Arts 29: 315. 1894.

**Type:** Mexico, Barranca de Beltran, 5-VI-1893, *C.G. Pringle 4379* (Holotype: GH00064512!).

**Distinguishing features**: Liana, climbing. Stems frequently with trichomes setose-glandular. Stipules 1.5–3 mm wide at base. Leaves 5–10 cm long. Leaflets 1.5–5 cm long. Inflorescences fasciculate. Flowers 8–20, up to 1.1 cm long. Calyx tube up to 4 mm long. Fruit and calyx glabrous or pubescent, without glandular setae. Fruits 4–4.5 cm long, the distal article with a wing of 1.8–2.6 cm long.

**Representative examined material:** Tamaulipas: 19-IX-1981, *Paul A. Fryxell 3688* (TEX00272695).

**Distribution:** Morphologically similar to *N. platycalyx*, but the latter with longer petals (1.4–2 cm long) and longer calyx tubes (4.5–6 mm long). In northeastern Mexico, central, west, and southern Mexico to Guerrero, in desert scrublands (Tamaulipan thornscrub), 50–150 m.

***Nissolia leiogyne*** Sandwith, Bull. Misc. Inform. Kew 1937: 302. 1937.

**Type:** Mexico, Guerrero, Coyuca, Santa Bárbara, 14-VII-1934, *G.B. Hinton 6291* (Isotype: GH00064516!, NY00026456!). Holotype not found.

**Distinguishing features:** Liana, climbing. Leaflets 0.5–3 cm long, obovate to suborbicular. Stipules 1 mm wide or less. Inflorescences fasciculate. Flowers 0.7–1 cm long. Calyx tube up to 2 mm long, its teeth 0.5–2 mm long. Fruit 2.5–3 cm. long, 2–3 articulate, glabrous or almost so, articles 5–7 × 5 mm, the distal one, sterile and winged, 1.5 × 0.6–0.8 cm.

**Representative examined material:** Coahuila: 5-V-1981, *D. Riskind 2344* (MEXU). Tamaulipas: 30-V-1986, *L. Hernández 1874* (UAT); 26-IV-1985, *M. Martínez 327*, *L. Hernández* (UAT).

**Distribution:** Rare in northeastern Mexico. Outside the study area, from Baja California Sur and Sonora to Oaxaca, along the Pacific slope. In desert scrublands, and chaparral, 1300–1500 m.

***Nissolia platycalyx*** S. Watson, Proc. Am. Acad. 17: 344. 1882.

**Type:** Mexico, Coahuila, Mountains, 6 miles east of Saltillo, Coahuila, VII-1880, *E. Palmer 248 in part* (Holotype: GH00064519!).

**Distinguishing features**: Climbing. Leaves 4–7 cm. long. Leaflets up to 2.5 cm long. Inflorescences 1–4-flowered. Flowers 1.4–2 cm long.; calyx 6.5–10 mm. long. Fruit 3–4 cm long, articles 2–4, pubescent when young, subglabrous with age, the distal article, sterile, winged, 2–3 cm. long and about 1 cm. wide.

**Representative examined material:** Coahuila: 4-VIII-1973, *J. Henrickson 11847* (ASU0036688!); 16-V-1973, *M.C. Johnston 11028* (ASU0036689!); Nuevo León: 23-VII-1999, *E. Estrada* et al. *11251* (CFNL); 23-VII-2002, *C. Yen y E. Estrada 15027* (CFNL, MEXU). 5-IX-1989, *Hinton* et al. *19614* (MEXU, TEX-LL); 8-IX-1962, *B.L. Turner y A.M. Powell 1078* (TEX-LL). 8-VIII-1988, *T.F. Patterson 6346* (TEX-LL). Tamaulipas: 10-IX-1986, *M. Martínez 1297* (UAT); 9-XII-1976, *F. González-Medrano 10163* (UAT); 20-VI-1986, *M. Martínez 1165* (UAT); 20-VI-1986, *M. Martinez 1165* (LL00272722!); 21-V-1982, *L.J. Dor*, *T. Atkins 2357* (LL00272742!).

**Distribution**: South of Texas (USA) and northeastern Mexico. Easily recognized by the size of its large flowers. Desert scrublands (Tamaulipan thorn scrub, piedmont scrub), oak-pine forest, and pine forest, 300–2100 m.

***Nissolia platycarpa*** Benth., in Mart. Fl. Bras. 15 (1): 77. 1859. Basionym: *Nissolia dodgei* Rose, Contr. U. S. Nat. Herb. 5: 161. 1899.

**Type:** Mexico, Zimapan, Hidalgo, non date, *Colulter n.n.* (Lectotype: K, Designated by Rose, 1899).

**Distinguishing features**: Climbing. Scandent vine. Leaves up to 8 cm. long. Flowers 0.8–1.2 cm long. Calyx 6–7 mm. long, pubescent or subglabrous, beset with glandular setae. Fruit up to 3.5 cm. Articles 2–5, densely pubescent, white-velutinous when young, glabrescent with age, with few yellowish, glandular setae, the distal article sterile, 1 cm long, 5–10 mm, wide.

**Representative examined material**: Coahuila: 25-VIII-1988, *J.A. Villarreal 4425* (ANSM); 21-VI-2007, *J. Encina D.*, *E. Mata Rocha*, *J. Partida Moncada*, *F. Hernández Soto 2477* (ANSM, TEX00437951!). Nuevo León: 27-VIII-1983, *M. Lavin 4515* (TEX-LL); 18-X-1959, *M.C. Johnston 4348B* (TEX-LL); 30-IX-1977, *J.G. Moya Rodríguez 3* (MEXU); 3-XI-1993, *J.A. Villarreal 7475* (ANSM, TEX-LL). Tamaulipas: 18-III-1987, *S. Ginzbarg 615* (TEX00272750);

**Distribution**: Endemic to Mexico, from Sonora to Tamaulipas, extending south to Puebla, Hidalgo, and Veracruz. This is the only species with white pubescent fruits and the fruits commonly the calyx with glandular setae in northeastern Mexico. Widely distributed in several plant communities, desert scrublands (Tamaulipan thornscrub, piedmont scrub), deciduous low forest, rain forest, 250–1600 m.

***Nissolia pringlei*** Rose, Contr. U. S. Nat. Herb. 5: 159. 1899. Basionym: *Nissolia diversifolia* Rose, Contr. U. S. Nat. Herb. 5: 160. 1899.

**Type:** Mexico, Chihuahua, Santa Eulalia Mountains, 15-IX-1895, *C.G. Pringle 324* (Holotype: US00001851!).

**Distinguishing features**: Climbing vine. Stems rarely with glandular setae. Stipules linear, 1 mm. wide or less. Flowers 0.8–1.3 cm. Calyx 4–6 mm. long, rarely glandular-setose, the tube 2–4 mm long. Fruit 2–3 cm. long, articles 2–5, pubescent, somewhat glabrescent, the terminal, article sterile, winged.

**Representative examined material**: Coahuila: 2-IX-1940, *I. M. Johnston*, *Cornelius H. Mueller 937* (LL00272763); 28-VIII-1971, *J.S. Henrickson 6149-9* (LL00272770). Nuevo León: 12-XI-1997, *M.A. Carranza 2719* (ANSM); 11-XI-1959, *M. Johnston*, *J. Graham 4630A* (1179282); VIII-1903, *C.G. Pringle 11813* (ASU0036677!).

**Distribution**: Endemic to Mexico. From Chihuahua, Coahuila, and Nuevo León to Querétaro, Morelos, and Puebla. Similar to *N. leiogyne*, although, the latter has a calyx tube (1–2 × 1.5 mm), and teeth (0.5–2 mm long) shorter than *N. pringlei*. In desert scrublands (Tamaulipan thornscrub and piedmont scrub), oak forest, and oak-conifer forest, 360–1890 m.

***Nissolia wislizeni*** (A. Gray) A. Gray, J. Proc. Linn. Soc., Bot. 5: 25. 1860. Basionym: *Chaetocalyx wislizeni* A. Gray, Smithsonian Contr. Knowl. 3(5): 51. 1852.

**Type:** Mexico, Chihuahua, [Battle-ground of Sacramento, near Chihuahua], nondate, *F. A. Wislizenus 151* (Holotype: H00053336!).

**Distinguishing features**: Herbaceous, prostrate. Stems pubescent and with yellowish, glandular setae. Leaves up to 5 cm. long: Leaflets elliptic to orbicular. Fruit mostly 2–4 cm long, articles 2–5 articulate, the fertile ones about 7–10 mm. long, the distal one, sterile article flat and winged, but almost as broad as the fertile ones.

**Representative examined material**: Coahuila: 21-V-1968, *A.M. Powell*, *D. Patterson*, *D. Ittner 1573* (LL00272805).

**Distribution**: From southwestern USA (Arizona), Sonora to Coahuila, extending south through Durango, Aguascalientes, Zacatecas, and San Luis Potosí to Jalisco. Easily recognized, because this is the only species with prostrate (non-climbing) stems. Rare in northeastern Mexico, mainly in desert scrublands, 500–1600 m.

***Stylosanthes*** Sw., Prodr. Veg. Ind. Occ.: 108. 1788. *Astyposanthes* Herter, Revista Sudamer. Bot. 7: 209. 1943.

**Type:** *Hedysarum hamatum* L., Syst. Nat., ed. 10. 2: 1170. 1759. *= Stylosanthes hamata* (L.) Taub.

Herbaceous, perennial, or rarely sub-shrubs. Leaves pinnately 3-foliolate. Stipules amplexicaul, divided at the apex into two teeth, adnate to the base of the petioles. Inflorescence terminal or axillary, spicate or globose, 1–30 flowered. Bracts simple, bidentate, or tridentate. Bracteoles 1–2. Fowers papilionate, yellow. Calyx 5-lobed, the two upper teeth completely united, its tube long. Corolla papilionate. Stamens 10, monadelphous, with 5 versatile anthers alternating with 5 sub-basifixed ones. Ovary 2-ovulate. Fruit a loment, 1–2 fertile articles, with the style persistent and forming a straight, sinuous, curved, hooked, or curled beak.

Several species of *Stylosanthes* are called tropical alfalfa, where these species are present, they are often eagerly selected by domestic livestock over other plant species that grow alongside, as is the case of *Stylosanthe mexicana* in the state of Nuevo León (pers. obs.). Another species cultivated and used as a forage species is *S. hamata* [49]. The genus *Stylosanthes* agglutinates approximately 25 species [1,50], mainly distributed in the tropics of America, Africa, Asia, and Malaysia [50]. On the American continent, it is distributed from eastern USA to northern Argentina and the Galapagos Islands. In Mexico, 11 species have been recorded [51], where four are found in northeastern Mexico.
1A.Stems with viscid pubescence, the plant sticky***S. viscosa***1B.Stems without viscid pubescence, the plant non-sticky22A.Fruit with 2 developed articles; flowers not subtended by an axis rudiment, at least the lower ones; bracteoles 1***S. humilis***2B.Fruit with 1 developed article (the upper one); flowers subtended by an axis rudiment; bracteoles 233A.Fruit pubescent on the main body and sometimes on the beak.***S. hamata***3B.Fruit glabrous or with some pubescence on the beak and sometimes on the nerves***S. mexicana***

***Stylosanthes hamata*** (L.) Taub., Verh. Bot. Brand. 32:22. 1890. *Anonis americana* Aubl. Hist. Pl. Guiane 2: 763 (1775). *Hedysarum hamatum* L., Syst. Nat., ed. 10. 2: 1170. 1759. *Ononis cerrifolia* Rchb. ex DC., Prodr. 2: 316. 1825. *Stylosanthes eriocarpa* S.F. Blake, Contr. U.S. Natl. Herb. 24: 4. 1922. *Stylosanthes humilis* Rich. ex Hemsl., Biol. Cent.-Amer., Bot. 1: 272. 1879. nom. illeg. *Stylosanthes procumbens* Sw., Prodr. Veg. Ind. Occ.: 108. 1788.

**Type:** Guatemala, Trail from Los Amates to Izabal, 31-V-1919, *S. F. Blake 7792* (Isotype: GH00026775!). Holotype not found.

**Distinguishing features**: Hebraceous, perennial, prostrate, not viscid. Leaflets 1.5 cm long, elliptic, with white veins, and appressed trichomes along margin abaxially, adaxially glabrous. Inflorescences in spikes, globose, 5–7 flowered. Flowers not subtended by an axis rudiment. Bracts similar to stipules, hyaline, with bristles. Outer bracteole 1. Inner bracteoles 2. Fruit with 2 articles, the upper one up to 7 mm fertile, the lower one 2 mm, abortive, pubescent, apically slightly incurved-beaked.

**Representative examined material**: Tamaulipas: 27-X-1959, *J.G. Graham*, *M.C. Johnston 4510* (TEX00274080).

**Distribution**. Rare, native from Mexico, Guatemala, Costa Rica, Colombia, Venezuela, and the Caribbean islands, it has been introduced into several countries in South America and Africa as a forage crop. Easily distinguished from *S. mexicana* because the latter has glabrous fruit or only slightly pubescent on the beak or rarely on its nerves, deciduous forest, tropical forest, 100–650 m.

***Stylosanthes humilis*** Kunth, Nov. Gen. Sp. 6: 506. 1824 (non *S. humilis* Rich. ex Hemsl.). Basionym: *Stylosanthes figueroae* Mohlenbr. Ann. Missouri Bot. Gard. 44: 342. 1957. *Astyposanthes humilis* (Kunth) Herter, Revista Sudamer. Bot. 7: 209. 1943. *Stylosanthes sundaica* Taub. Verh. Bot. Vereins Prov. Brandenburg 32: 21. 1890.

**Type**: Venezuela. Carichaná, non date, *A.J.A. Bonpland*, *F.W.H.A. von Humboldt*, *n.n.* (Holotype: PP00659968!).

**Distinguishing features**: Herbaceous, perennial, prostrate, non-viscid. Stems not viscid, with bristles up to 4 mm and incurved white trichomes. Leaflets 1.1 cm long, with bristles and gland-dotted abaxially. Inflorescences distal, up to 18-flowers. Flowers subtended by an axis rudiment, at least the lower ones. Inner bracteoles 1. Bracts similar to stipules. Fruit with 2 fertile, developed articles, the lower one dense yellowish pubescent, 2 mm long, the upper one sparsely pubescent, up to 7 mm long. Beak 4 mm, almost equal or larger than segment, elongated and curved apically.

**Representative examined material**: Tamaulipas: 8-VIII-1941, *L.R. Stanford*, *K.L. Retherford*, *R.D. Northcraft 793* (LL00274084); 7-X-1966, *J. A. Mears 531-A* (TEX00274082).

**Distribution**: Widely distributed, from south USA (Arizona and Florida), to northern Mexico (Sonora and Tamaulipas), extending south through the states on both sides, Pacific and Gulf, to Chiapas, Central America, Colombia to Brazil. Rare in the study area, in deciduous forest, abandoned fields, and side roads, 200–1600 m.

***Stylosanthes mexicana*** Taub., Verh. Bot. Vereins Prov. Brandenburg 32: 21. 1890. Basionym: *Stylosanthes bangii* Taub., Mem. Torrey Bot. Club 4: 206. 1895.

**Type:** Mexico, 1-I-1879, J.G. Schaffner 579 (Isotype: CM1105!). Holotype not found.

**Distinguishing features**: Stem ascending to spreading, without viscid pubescence, the plant non-sticky. Stems up to 30 cm tall, commonly branched near the base, densely appressed pubescent with 3–4 pairs of white veins abaxially. Inflorescences sub-oblong, relatively dense, up to 1.5 cm long. Outer bracteole 1, bifid, up to 4.5 mm long; axis rudiment to 6 mm. long in fruit, long white-ciliate. Inner bracteoles 2. Fruit with 2 articles, the upper one up to 5 mm long, fertile, the lower one shorter, abortive, glabrous or with some pubescence on the recurved-uncinate or sometimes with a half-coiled beak, and sometimes on the nerves, reticulate-nerved.

**Representative examined material**: Coahuila: 23-XI-1979, non colector (MEXU461656). Nuevo León: 23-VII-2002, *C. Yen y E. Estrada 15084* (CFNL); 22-X-2002, *C. Yen y E. Estrada 15164* (CFNL); 13-VII-2002, *C. Yen y E. Estrada 14783* (CFNL, MEXU); 23-VII-2002, *C. Yen y E. Estrada 15044* (CFNL); 18-V-1986, *E. Estrada 565* (CFNL, MEXU); 13-IX-1991, *Hinton* et al. *21471* (TEX-LL). Tamaulipas: 27-X-1959, *M.C. Johnston 4510* (MEXU); 15-XI-1963, *H.S. McKee 10961* (MEXU); 29-VI-1985, *L. Hernández 01506* (UAT, MEXU).

**Distribution:** From northeastern Mexico and Sinaloa, through San Luis Potosí and Querétaro to Guanajuato, Nayarit, and Hidalgo. Disjunct in Venezuela and Bolivia. Frequently in desert scrublands, oak, pine-oak forests, and conifers forests, frequently along roads, 360–1530 m. Forage species, highly sought after by domestic livestock that graze freely along roads and abandoned crop areas (pers. obs.).

***Stylosanthes viscosa*** (L.) Sw., Prodr. Veg. Ind. Occ.: 108. 1788. Basionym: *Astyposanthes viscosa* (L.) Herter, Revista Sudamer. Bot. 7: 209. 1943. *Stylosanthes debilis* M.B. Ferreira & Sousa Costa, Anais Congr. Soc. Bot. Brasil 28: 83. 1977 (publ. 1978). *Stylosanthes glutinosa* Kunth, Nov. Gen. Sp. 6: 507. 1824. *Stylosanthes pilosa* M.B. Ferreira & Sousa Costa, Anais Congr. Soc. Bot. Brasil 28: 89 1977 (publ. 1978). *Stylosanthes prostrata* M.E. Jones, Contr. W. Bot. 15: 135. 1929. *Stylosanthes viscosa* f. *typica* Hassl., Repert. Spec. Nov. Regni Veg. 16: 220. 1919. *Stylosanthes viscosa* var. *minor* Micheli, Vidensk. Meddel. Naturhist. Foren. Kjøbenhavn 1875: 72. 1876.

**Type**: JAMAICA. not locality, 1759, *H. Sloane 1696*, (lectotype: P! designated by Kirkbride & Kirkbride, 1987).

**Distinguishing features:** Herbaceous perennial. Stems decumbent with white dense pubescence and spread glandular bristles, so the stems viscid, glutinous, and sticky. Leaves trifoliate. Leaflets 1–2 cm long, white veined abaxially. Inflorescences in globose or oblong-globose spikes. Bracts similar to stipules. Inner bracteoles 2 at base of each flower. Flowers 4.5 mm. Hypanthium 3 mm. Calyx 2 mm, inferior tooth largest. Fruit with only 1 article developed, 4 mm, reticulate, pubescent, and rounded reddish glands, beaked, the beak curved, pubescent as the article.

**Representative examined material**: Tamaulipas: 27-IX-1959, *M. Johnston*, *J. Graham 4078B* (MICH1183109); 30-VII-1957, *R.L. Dressler 2092* (MICH1183112).

**Distribution:** Widely distributed, from south USA, through Mexico including all the states along both the Gulf and Pacific coasts, extending south to Bolivia and Brazil. Easily distinguished from the rest of the species because it is the only species with viscid stems. Desert scrublands, and low deciduous forest, 250–760 m.

***Zornia*** J. Gmel. Syst. 1076. 1791. *Myriadenus* Desv. Journ. Bot. 3: 121. 1813.

**Type:** *Zornia bracteata* J.F. Gmel., Syst. Nat., ed. 13[bis].: 1096. 1792.

Perennial herbaceous, rarely sub-shrubs. Leaves bifoliolate or tetrafoliolate. Stipules, paired, peltate. Inflorescences axillary or distal, spicate or racemose, 1-few flowers. Bracts paired, peltate. Flowers papilionate, yellow, orange-yellow, rarely white. Calyx 5-dentate, the tube short. Stamens 10, monadelphous. Anthers 5 versatile anthers and 5 sub-basifixed. Fruit a loment with 2–15 articles.

The species of *Zornia* are distributed in tropical and subtropical areas of the world. The genus is composed of approx. 75 species, 11 of which occur in Mexico and Central America [52]. Four species were recorded in northeastern Mexico.
1A.Leaflets four per leaf***Z. bracteata***1B.Leaflets two per leaf22A.Upper and lower leaves with leaflets similar in shape, linear, although the upper ones smaller***Z. lasiocarpa***2B.Upper and lower leaves with leaflets different in shape, the upper ones linear to lanceolate, the lower ones shorter and broader33A.Fruit included within the bracts, or only 1–2 articles exserted of the bracts***Z. reticulata***3B.Fruit conspicuously exserted of the bracts***Z. latifolia***

***Zornia bracteata*** (Walt.) J.F. Gmel., Syst. Nat., ed. 13[bis].: 1096. 1792. Basionym: *Hedysarum tetraphyllum* Thunb., Nova Acta Regiae Soc. Sci. Upsal., ser. 2, 6: 44. 1799. *Zornia tetraphylla* Michx. Fl. Bor.-Amer. 2: 76. 1803.

**Type:** USA, 3 miles west of Marion, 7-VII-1927, *K.M. Wiegand*, *W.E. Manning; 1624* (Neotype: GH00277013!).

**Distinguishing features:** Herbaceous, perennial. Stems up to 0.8 m tall. Leaves upper and lower with leaflets of different shapes, the upper ones linear to lanceolate, the lower ones shorter and broader. Leaflets 4 per leaf, 1-nerved abaxially, up to 2.5 cm long. Stipules 5–7 nerved. Inflorescence crowded or interrupted. Bracts ovate, up to 1 cm long, 5–7 nerved. Fruit, a loment 3.5 mm long, 2–6 articled, non- reticulate, with abundant retrorse hairy bristles up to 1 mm long.

**Representative examined material**: Tamaulipas: II-1996, *M. González G. 160* (MEXU);

**Distribution:** In states on the eastern coast of the USA up to Tamaulipas. Rare, in desert scrublands and tropica forest, 200–750 m.

***Zornia lasiocarpa*** A. Molina, in Ceiba 1: 257. 1951.

**Type:** Honduras, 23-XI-1948, *A. Molina R. 1660* (Isotype: F0059982F!, GH00241663!). Holotype not found.

**Distinguishing features:** Herbaceous, perennial. Stems up to 30 cm tall, branched, glabrous. Upper and lower leaves with leaflets similar in shape, linear, although the upper ones smaller. Leaflets 2, linear-filiform, punctate, glabrous. Stipules punctate. Fruit a loment 3-articled, each 2.5 × 2.0 mm, shallowly reticulate, pilose, with abundant retrorsely bristles to 1.5 mm long.

**Representative examined material**: Tamaulipas: 27-X-1959, *Marshall C. Johnston*, *J. G. Graham 4549* (TEX00274451).

**Distribution:** Distributed in Mexico (Sinaloa, Tamaulipas, and Chiapas), Honduras, and Venezuela. Species easily distinguished from other species by its linear leaflets. In our study area, recorded only in the southern area (municipality of Altamira) of the state of Tamaulipas, desert scrublands, deciduous forest tropical forest, oak forest, and disturbed areas, 130–1230 m.

***Zornia latifolia*** Sm., Cycl. 39: n.° 4. 1818. Basionym: *Hedysarum gemellum* Willd. ex Vogel, Linnaea 12: 61. 1838. nom. inval. *Zornia diphylla* subsp. *gracilis* (DC.) Malme, Ark. Bot. 23A(13): 76. 1931. *Zornia diphylla* var. *bernardinensis* Chodat & Hassl., Bull. Herb. Boissier, sér. 2, 4: 887. 1905. *Zornia diphylla* var. *gracilis* (DC.) Benth., Fl. Bras. 15(1): 83. 1859. *Zornia gemella* (Willd.) Vogel, Linnaea 12: 61. 1838. *Zornia gracilis* DC., Prodr. 2: 316. 1825. *Zornia latifolia* var. *bernardinensis* (Chodat & Hassl.) Mohlenbr., Webbia 16: 128. 1961. *Zornia maranhamensis* G. Don, Gen. Hist. 2: 280. 1832. *Zornia surinamensis* Miq., Ann. Mag. Nat. Hist. 11: 14. 1843.

**Type:** Paraguay, nondate, *E. Hassler 5115* (Isotype: NY00050717!). Holotype not found.

**Distinguishing features:** Herbaceous, perennial. Stems erect, up to 50 cm tall, branched, glabrate or pilose. Leaflets 2, up to 2.5 cm, punctate, 1-nerved. Stipules often punctate, 5–7 nerved. Inflorescence distal, crowded, interrupted below. Bracts oblong-lanceolate or lanceolate, usually punctate, 5-nerved. Fruit with 5–7 articles conspicuously exserted of the bracts. Articles 1.6–2 × 1.4–1.7 mm, non-reticulate or faintly reticulate, pilose, with abundant retrorsely bristles 0.2–1.0 mm long, eglandular.

**Representative examined material**: Tamaulipas: 9-XI-1994. *Hinton 25050* (TEX00274445).

**Distribution**: Widely distributed, from southern USA (Texas), northeastern Mexico, Central America, Antilles, to Brazil and Argentina. Oak forest, 600–900 m.

***Zornia reticulata*** Sm., Cycl. 39: n.° 2. 1818. Basionym: *Hedysarum bifolium* Vell., Fl. Flumin.: 318. 1829. *Zornia barbata* Desv., Mém. Soc. Linn. Paris 4: 325. 1826. *Zornia cuyabensis* Malme, Ark. Bot. 23A(13): 75. 1931. *Zornia diphylla* f. *ciliata* Chodat & Hassl., Bull. Herb. Boissier, sér. 2, 4: 887. 1904. *Zornia diphylla* f. *diversifolia* Chodat & Hassl., Bull. Herb. Boissier, sér. 2, 4: 887. 1904. *Zornia diphylla* f. *intermedia* Chodat & Hassl., Bull. Herb. Boissier, sér. 2, 4: 888. 1904. *Zornia diphylla* subsp. *cuyabensis* Malme, Ark. Bot. 23A(13): 75. 1931. *Zornia diphylla* subsp. *reticulata* (Sm.) Malme, Ark. Bot. 23A(13): 25. 1931. *Zornia diphylla* subsp. *subperforata* Malme, Ark. Bot. 23A(13): 75. 1931. *Zornia diphylla* var. *elatior* Micheli, Bull. Herb. Boissier 6(App. 1): 33. 1898. nom. illeg. *Zornia diphylla* var. *paraguariensis* Chodat & Hassl., Bull. Herb. Boissier, sér. 2, 4: 887. 1904. *Zornia diphylla* var. *pubescens* (Kunth) Benth., Fl. Bras. 15(1): 82. 1859. *Zornia diphylla* var. *reticulata* (Sm.) Benth., Fl. Bras. 15(1A): 81. 1859. *Zornia diphylla* var. *rupestris* Chodat & Hassl., Bull. Herb. Boissier, sér. 2, 4: 888. 1904. *Zornia diphylla* var. *stenophylla* Griseb., Cat. Pl. Cub.: 72. 1866. *Zornia diphylla* var. *stricta* Benth., Fl. Bras. 15(1A): 81. 1859. *Zornia echinata* Mohlenbr., Webbia 16: 132. 1961. *Zornia havanensis* A. Rich., Hist. Phys. Cuba, Pl. Vasc.: 423. 1846. *Zornia inermis* Desv., Mém. Soc. Linn. Paris 4: 325. 1826. *Zornia ovata* Vogel, Linnaea 12: 58. 1838. *Zornia pubescens* Kunth, Nov. Gen. Sp. 6: 515. 1824. *Zornia reticulata* var. *puberula* DC., Prodr. 2: 316. 1825. *Zornia reticulata* var. *punctata* Vogel, Linnaea 12: 58. 1838. *Zornia subperforata* Malme, Ark. Bot. 23A(13): 26. 1931.

**Type:** Jamaica, St. Catherine, 8-VI-1915, *W.H. Harris 12070* (Neotype: US01003353!).

**Distinguishing features:** Herbaceous, perennial. Upper and lower leaves with leaflets different in shape, the upper ones linear to lanceolate, the lower ones shorter and broader. Leaflets 2. Inflorescence compact. Fruit a loment 4–7 articles, including whitened bracts, or only 1–2 articles exserted from the bracts.

**Representative examined material**: Nuevo León-Tamaulipas border: 9-XI-2001, *E. Estrada 13188* (CFNL). Tamaulipas: 22-X-1999, *A. Mora-Olivo 7710* (UAT); 23-IX-1985, *M. Yanez 542* (UAT); 13-X-1987, *L. Hernández*, *M. Martínez 2217* (TEX00274469); 22-VII-2008. *R. Dragustinovis E. Martínez*, *S.A. Ibarra 40338* (MEXU); 22-IX-1956, *F. Martínez Martínez*, *G. Borja Luvando F-1930* (TEX00274457); 23-VII-1957, *R.L. Dressler 1965* (MEXU); 19-VII-1959, *E.F. Anderson 1150* (RSA0156905, RSA0156905).

**Distribution:** South of USA (Arizona), through Mexico, Central America to Ecuador, Paraguay, and Brazil. In rocky soils, piedmont scrub chaparral (dwarf oaks), rain forest, and oak forest, 400–1150 m.

## 3. Discussion

The Amorpheae is the tribe with higher species richness in the study area, followed by Dalbergieae and Brongniarteae. The surface area of the three states of northeastern Mexico reaches 296,000 km^2^, while Mexico reaches almost 2,000,000 km^2^. In terms of surface area, 14.8% of the surface of northeastern Mexico houses 27% of the genera and 20% of the species of the tribes Amorpheae, Brongniartieae, and Dalbergieae occurring in Mexico. The tribe Amorpheae is widely distributed from southern Canada to Argentina [1,29], but Mexico has ca. 78% of the species of this group (200–210 species). In the northeastern region of Mexico, 17% and 44% of all species and genera, respectively, of Amorphae present in Mexico are recorded. Of the approximately 248 species of Amorpheae reported by Lewis et al. [1], around 10–12 new species, especially from the genus *Dalea* [35] have been described from 2006 to date, bringing now approximately a total of 258–260 species within nine genera of the tribe Amorpheae, including *Psorodendron*, recently resurrected by Piñeros et al. [21]. Of the 10 genera that are considered within the tribe Brongniartieae [1], two of them are found in Mexico, *Brongniartia* and *Harpalyce*. *Brongniartia* is typically considered a Mexican genus [39], where more than 95% of the 63 species are found in the country. One-third of all species are microendemic [40], recorded only in the type localities, as is the case of many species from the Balsas region, in southwestern Mexico [40]. Only 8% of *Brongniartia* species are recorded in northeastern Mexico and are those with the widest distribution areas in Mexico, except for *B. rozynskii*, endemic to Tamaulipas. *Harpalyce* with three recognized sections has disjunct distribution patterns [39]; sect. *Harpalyce* has species restricted to Mexico, Guatemala, and Honduras; sect. *Cubenses* include species that are restricted to Cuba, and sect. *Brasilianae* considers species restricted to Brazil [39]. In northeastern Mexico, 43% of the species are found but located only in areas with tropical and subtropical climates, in the extreme south of the state of Tamaulipas. *Dalea* is the genus considered in this study with the highest species richness; in addition, its species inhabit practically all plant communities, especially those that are found in semi-arid and cool areas in low plains and mountainous areas, where desert scrub, gypsophilous grasslands, and oak-pine forests predominate. In northeastern Mexico, *Dalea* represents 28% of the total species present in Mexico (125 species) and 14% of this total are endemic to northeastern Mexico. Of the four species of *Eysenhardtia* present in Mexico, two of them are endemic to the northeastern region. The tribe Dalbergieae in northeastern Mexico is represented by 52% of the extant genera in Mexico, excpet for *Andira*, *Chaetocalyx*, *Chapmannia*, *Machaerium*, *Platymiscium*, *Poiretia*, *Pterocaprus*, and *Vatairea*. The most diverse genera of this tribe in northeastern Mexico are *Nissolia*, *Stylosanthes*, and *Zornia*, highlighting that the four species of *Stylosanthes* reported for Mexico are all distributed in northeastern Mexico, especially in the state of Tamaulipas. It is worth noting that more than half of the species of *Nissolia* reported for Mexico are found in the northeastern part of the country, present in desert scrub, oak, and coniferous forests, as well as low deciduous forests. Other genera such as *Ctenodon*, *Dalbergia*, and *Diphysa* are poorly represented in the study region, being more diverse in southern Mexico. It is interesting to note that most of the 63 of the 75 registered species of the Amorpehae, Brongniartieae, and Dalbergieae tribes, are found in the desert scrublands, with its multiple variants, among which the Tamaulipan thornscrub (100–350 m), piedmont scrub (450–850 m), microphyllous scrub (especially constituted by *Larrea tridentata*) (1300–1800 m), and rosetophyllous scrub (1400–1700 m) stand out. The main life forms of these species are herbaceous and shrubby, predominantly inhabiting calcareous soils, where *Dalea* species are the most frequent, many of which are endemic to desert scrub. The second highest species richness of the above-mentioned tribes is recorded in the oak-pine forest (31 taxa). This community is altitudinally and latitudinally continuous throughout the mountains of northeastern Mexico, between the altitudinal ranges of 850–2600 m, presenting notable contrasts in its physiognomy and species composition according to the different altitudinal ranges and exposure. In this plant community, several species of the genera *Dalea*, *Marina*, *Brongniartia*, *Harplayce*, *Nissolia*, *Stylosanthes*, and *Zornia* stand out for their richness. The oak forest (21 species) and the coniferous forest (15 species) are home to a lower species richness of the three tribes, where several endemic species of *Dalea* stand out, as well as species of *Brongniartia*, *Nissolia*, and *Stylosanthes*. The tropical evergreen forest (7 species) and the low deciduous forest (15 species) are plant communities located in the extreme southeast of the study area, south of the Tropic of Cancer, in the tropical portion of Mexico, from the coast to part of the low plains to the mountain slopes, 90–650 m, and are not characterized by having a high number of species that belong to these three tribes. Several species, such as *Aeschynomene americana*, *Ctenodon fascicularis*, *Dalbergia brownei*, *Diphysa americana*, and *Stylosanthes hamata*, are characteristic of these plant communities and are often absent in other ones. Other plant communities, such as the Chaparrales of Fagaceae (*Quercus* spp.) and Rosaceae (*Cercocarpus*, *Malacomeles*, and *Lindleya*), as well as the halophytic or gypsophilous grasslands, host a few species of legumes, although some of them are notable for their regional endemism, as is the case of *Dalea gypsophila*, *D. radicans*, and *D. parrasana*.

## 4. Materials and Methods

### 4.1. Study Area

**Figure 4:**
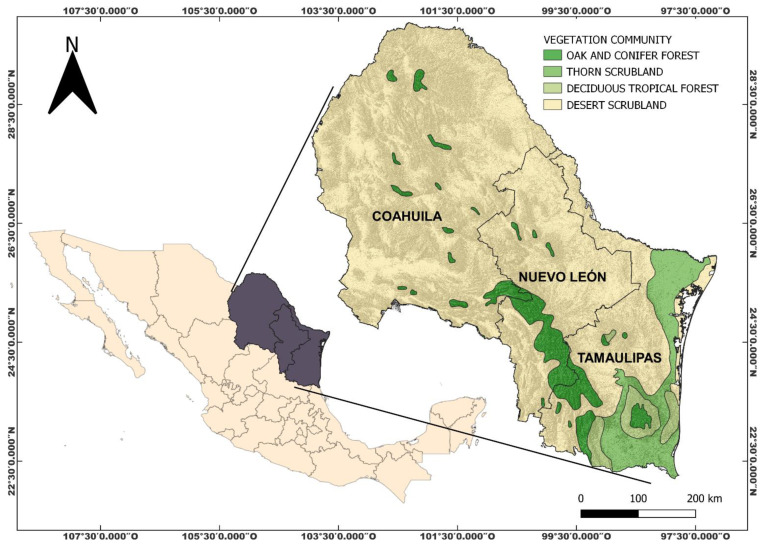
Study area and vegetation types. Northeastern Mexico comprises three states, Coahuila, Nuevo León, and Tamaulipas.

Here northeastern Mexico is considered a group of three states, Coahuila, Nuevo León, and Tamaulipas. Together they comprise 296,000 km^2^ (Figure 4) and are immersed within three physiographic provinces, the Northern Gulf Coast Plain, the Great Plain of North America, and the Sierra Madre Oriental; within this area, there are several soil types with diverse chemical properties [53,54,55].

The relief is characterized by large areas of low plains, between 50 and 370 m [56], housing thorny scrub and low deciduous forest [56], where legumes are predominant elements [56]. Those plant communities are mainly found in the easternmost portion of the study area, the entire coast of the state of Tamaulipas, up to the base of the Sierra Madre Oriental in Nuevo Léon. On the slopes and foothills of the Sierra Madre Oriental, 450–850 m, there are communities of submontane scrub and low deciduous forests, with denser coverage and greater heights than those of the scrub in the lower parts [57]. The highest parts of the mountains that begin in the low plains reach 1300–1600 m latitude on average [58], here oak-pine forests predominate. On the leeward side of the Sierra Madre Oriental, between 1600 and 1900 m, humidity gradually decreases and vegetation gradually changes, transforming into communities of low coniferous forests (*Pinus cembroides*-*Juniperus* spp.) and oak thickets (*Quercus* spp.), as well as desert thickets of *Larrea tridentata* associated with species of *Flourensia*, *Senegalia*, and *Vachellia*, frequently associated with members of Asparagaceae and Cactaceae [53,54,55]. Within those desert scrublands, there are portions of gypsophilous grassland associated with gypsum and calcareous soils, where there is a large number of endemic species, highlighting several legumes [58]. The mountain peaks above 1900 m, have oak groves and coniferous forests with different genera where *Abies*, *Picea*, *Pinus*, and *Pseudotsuga* stand out. At the top of the highest peaks, the subalpine prairie is found [57,58], between 3500 and 3650 m elevation, with vegetation and flora that is heterogeneous to the rest of the plant communities.

Along the altitudinal and latitudinal gradient in the study area, the Fabaceae plays an important role from the ecological and diversity points of view. Its predominance is evident not only in coverage and density but also in species richness, because they are the dominant elements in almost all the scrublands that are found in the area, especially in the lower parts (80–500 m), its dominance in coverage and density decreases in places with the highest elevations within the study area, but its species richness is equally important, such as the case of Asteraceae and Poaceae [11].

### 4.2. Taxonomic Treatment

As we have conducted in our two previous studies regarding the Fabaceae Family in northeastern Mexico [59,60], the first phase of this study consisted of compiling in a database the diversity of the Fabaceae, tribes Amorpheae, Brongniartieae, and Dalbergieae, present in northeastern Mexico published in the scientific literature and available in databases on the Internet. This database was completed with the authors’ personal collection records covering a period of 40 years. The final part of this phase was to review the herbaria ANSM, CFNL, MEXU, UAA, UAT, and UNL, where the largest number of botanical specimens from the study area are stored, as well as to study high-resolution photographs from the databases of the herbaria CAS, MICH, NY, TEX. Type specimens were studied using the Jstor Global Plants platform [61] was consulted. The Tropicos platform [62] and the book Order out of Chaos [63] were consulted to obtain lectotype designation. In the representative material examined sections, the symbol “!” indicates that the type of specimen of the species was studied by the authors. The scientific names and their accepted synonymy are based on the WFO [64]. Artificial dichotomous identification keys were elaborated to discriminate and differentiate the taxa under consideration. Measurements of the morphological characters were carried out by the authors; however, when the specimen(s) were not available, the morphological measurements were obtained from the literature. The type of specimen information was added to the description of each genus and species, as well as its synonymy (heteronyms, homonyms). To facilitate the recognition of the taxa, a brief morphological description of each species was included. A comment section was added after the representative examined the material section, which includes information regarding endemism, ecology, and distribution of the species in the study area and out of it. The tribes, genera, and species were arranged alphabetically.

## Figures and Tables

**Table 1:** Number of taxa in the world, in Mexico, and northeastern Mexico, belonging to the tribes Amorpheae, Brongniartieae, and Dalbergieae of the subfamily Papilionoideae.

Tribe and Number of Genera	Species in the World	Species in Mexico	Species in NE Mexico
**Amorpheae**			
1. *Amorpha*	15	15	1
2. *Dalea*	175	125	35
3. *Eysenhardtia*	13	13	4
4. *Marina*	38	38	2
5. *Psorothamnus*	9	4	1
**Brongniartieae**			
6. *Brongniartia*	≈50	≈45	5
7. *Harpalyce*	24	7	3
**Dalbergieae**			
8. *Aeschynomene*	170	40	3
9. *Amicia*	7	1	1
10. *Arachis*	65	1	1
11. *Ctenodon*	≈120	22	1
12. *Dalbergia*	250	20	1
13. *Diphysa*	15	10	2
14. *Nissolia*	13	13	7
15. *Stylosanthes*	25	6	4
16. *Zornia*	75	14	4

**Table 2:** Endemism of the tribes Amropheae, Brongniatieae, and Dalbergieae in Mexico and northeastern Mexico.

Region/Tribe	Amorpheae	Brongniartieae	Dalbergieae
Endemic to Mexico	*Dalea boraginea*, *D. botteri*, *D. capitata* var. *capitata*, *D. capitata* var. *lupinocalyx*, *D. capitata* var. *pseudohospes*, *D. dorycnioides*, *D. eriophylla*, *D. gypsophila*, *D. hospes*, *D. luisana*, *D. lutea* var. *lutea*, *D. melantha* var. *berlandieri*, *D. melantha* var. *melantha*, *D. melantha* var. *pubens*, *D. neomexicana* var. *megaladenia*, *D. obovatifolia* var. *obovatifolia*, *D. parrasana*, *D. prostrata*, *D. radicans*, *D. saffordii*, *D. uniflora*, *Eysenhardtia parviflora*, *E. polystachya*, *E. schizocalyx*, *Marina filiciformis*, *M. nutans*	*Brongniartia discolor*, *B. intermedia*, *B. magnibracteata*, *B. rozynskii*, *Harpalyce arborescens*, *H. formosa*, *H. mexicana*	*Amicia zygomeris*, *Diphysa microphylla*, *Nissolia platycarpa*, *N. pringlei.*
Endemic of NE Mexico	*Dalea boraginea* (Coahuila), *D. capitata* var. *lupinocalyx* (Nuevo León), *D. capitata* var. *pseudohospes* (Nuevo León and Tamaulipas), *D. gypsophila* (Nuevo León), *D. melantha* var. *pubens* (Coahuila), *D. neomexicana* var. *megaladenia* (Coahuila), and *D. uniflora* (Nuevo León).		

## Data Availability

The data presented in this study are available on request from the corresponding author.

## References

[1] Lewis G.P., Schrire B., MacKinder B., Lock M. (2005). Legumes of the World.

[2] LPWG (2013). Legume phylog-eny and classification in the 21st century: Progress, prospects and lessons for other species-rich clades. Taxon.

[3] Sprent J.I., Parsons R. (2000). Nitrogen fixation in legume and non-legume trees. Field Crops Res..

[4] Estrada-Castillón E., Garza-López M., Villarreal-Quintanilla J.Á., Salinas-Rodríguez M.M., Soto-Mata B.E., González-Rodríguez H., González-Uribe D.U., Cantú-Silva I., Carrillo-Parra A., Cantú-Ayala C. (2014). Ethnobotany in Rayones, Nuevo León, Mexico. J. Ethnobiol. Ethnomed..

[5] Estrada-Castillón E., Villarreal-Quintanilla J.A., Mora-Olivo A., Cuéllar-Rodríguez G., Sánchez-Salas J., Gutiérrez-Santillán T.V., Valdes Alameda R., González-Cuéllar D.A., González-Montelongo C., Arévalo Sierra J.R. (2023). Ethnobotany of the Useful Native Species in Linares, Nuevo León, México. Sustainability.

[6] Sousa M., Ricker M., Hernández H.M. (2001). Tree species of the family *Leguminosae* in Mexico. Harvard Pap. Bot..

[7] Estrada Castillón E., Villarreal Quintanilla J.A., Rodríguez Salinas M.M., Encinas Domínguez J.A., González Rodríguez H., Romero Figueroa G., Arévalo J.R. (2018). Ethnobotanical Survey of Useful Species in Bustamante, Nuevo León, Mexico. Hum. Ecol..

[8] Estrada-Castillón E., Villarreal-Quintanilla J.A., Cuéllar-Rodríguez L.G., March-Salas M., Encina-Domínguez J.A., Himmeslbach W., Salinas-Rodríguez M.M., Guerra J., Cotera-Correa M., Scott-Morales L.M. (2022). Ethnobotany in Iturbide, Nuevo León: The Traditional Knowledge on Plants Used in the Semiarid Mountains of Northeastern Mexico. Sustainability.

[9] França Benjamim J.K., Albuquerque Dias da Costa K., Silva Santos A. (2020). Chemical, Botanical and Pharmacological Aspects of the *Leguminosae*. Pharmacogn. Rev..

[10] Reed J.D. (1995). Nutritional toxicology of tannins and related polyphenols in forage legumes. J. Anim. Sci..

[11] Estrada C.E., Delgado-Salinas A., Villarreal Q.J. (2014). Leguminosas de Nuevo León, México.

[12] Clarke H.D., Downie S.R., Seigler D.S. (2000). Implications of chloroplast DNA restriction site variation for systematics of *Acacia* (Fabaceae: Mimosoideae). Syst. Bot..

[13] Bruneau A., Forest P., Herendeen P.S., Klitgaard B.B., Lewis G.P. (2001). Phylogenetic relationships in the Caesalpinioideae (*Leguminosae*) as inferred from chloroplast *trnL* intron sequences. Syst. Bot..

[14] Wojciechowski M.F., Lavin M., Sanderson M.J. (2004). A phylogeny of legumes (*Leguminosae*) based on analysis of the plastid *matK* gene resolves many well-supported subclades within the family. Am. J. Bot..

[15] Zhao Y., Zhang R., Jiang K., Qi J., Hu Y., Guo J., Zhu R., Zhang T., Egan A.N., Yi T.S. (2021). Nuclear phylotranscriptomics/phylogenomics support numerous polyploidization events and hypotheses for the evolution of rhizobial nitrogen-fixing symbiosis in Fabaceae. Mol. Plant.

[16] de la Estrella M., Forest F., Klitgard B., Lewis G.P., Mackinder B.A., de Queiroz L.P., Wieringa J.J., Bruneau A. (2018). A new phylogeny-based tribal classification of subfamily Detarioideae, an early branching clade of florally diverse tropical arborescent legumes. Sci. Rep..

[17] Catalano S.A., Vilardi J.C., Tosto D., Saidman B.O. (2008). Molecular phylogeny and diversification history of *Prosopis* (Fabaceae: Mimosoideae). Biol. J. Linn. Soc..

[18] Marazzi B., Endress P.K., De Queiroz L.P., Conti E. (2006). Phylogenetic relationships within Senna (*Leguminosae*, Cassiinae) based on three chloroplast DNA regions: Patterns in the evolution of floral symmetry and extrafloral nectaries. Am. J. Bot..

[19] Zhang R., Wang Y.H., Jin J.J., Stull G.W., Bruneau A., Cardoso D., De Queiroz L.P., Moore M.J., Hang S.D., Chen S.Y. (2020). Exploration of plastid phylogenomic conflict yields new insights into the deep relationships of *Leguminosae*. Syst. Biol..

[20] Hughes C.E., Ringelberg J.J., Lewis G.P., Catalano S.A. (2022). Disintegration of the genus *Prosopis* L. (*Leguminosae*, Caesalpinioideae, mimosoid clade). PhytoKeys.

[21] Piñeros Urrego P., Suárz-Baron H., Pavón-Mora N., González F. (2023). Reinstatement of the genus *Psorodendron* and related systematic novelties as revealed from phylogenetic analyses of the tribe Amorpheae (*Leguminosae*, Papilionoideae). Caldasia.

[22] Gagnon E., Bruneau A., Hughes C.E., Paganucci de Queiroz L., Lewis G.P. (2016). A new generic system for the pantropical *Caesalpinia* group (*Leguminosae*). PhytoKeys.

[23] Ringelberg J.J., Koenen E.J.M., Iganci J.R., de Queiroz L.P., Murphy D.J., Gaudeul M., Bruneau A., Luckow M., Lewis G.P., Hughes C.E. (2022). Phylogenomic analysis of 997 nuclear genes reveals the need for extensive generic re-delimitation in Caesalpinioideae (*Leguminosae*). PhytoKeys.

[24] Lavin M., Pennington R.T., Klitgard B.B., Sprent J.I., Cavalcante de Lima H., Gasson P.E. (2001). The Dalbergioid legumes (Fabaceae) delimitation of the of a pantropical monophyletic clade. Am. J. Bot..

[25] LPWG (2017). A new subfamily classification of the *Leguminosae* based on a taxonomically comprehensive phylogeny. Taxon.

[26] Cardoso D., Pennington R.T., Queiroz L.P., Boatwright J.S., Van Wyk B.E., Wojciechowski M.F., Lavin M. (2013). Reconstructing the deep-branching relationships of the papilionoid legumes. S. Afr. J. Bot..

[27] LPWG The World Checklist of Vascular Plants (WCVP): Fabaceae, vers. June 2021. Govaerts, R. ed. http://sftp.kew.org/pub/data_collaborations/Fabaceae/DwCA/.

[28] Sousa S.M., Delgado A., Ramamoorthy T.P., Bye R., Lot A., Fa J. (1993). Mexican *Leguminosae*: Phytogeography, endemism and origins. Biological Diversity of Mexico.

[29] Barneby R.C. (1977). Daleae imagines. An illustrated revision of *Errazurizia* Philippi, *Psorothamnus* Rydberg, *Marina* Liebmann, and *Dalea* Lucanus emend. Barneby, including all species of *Leguminosae* tribe *Amorpheae* Borissova ever referred to *Dalea*. Mem. N. Y. Bot. Gard..

[30] McMahon M., Hufford L. (2004). Phylogeny of Amorpheae (Fabaceae: Papilionideae). Am. J. Bot..

[31] Ross J.H., Crisp M.D., Lewis G., Schrire B., Mackinder B., Lock M. (2005). Brongniartieae. Legumes of the World.

[32] Klitgaard B., Lavin M., Lewis G., Schire B., Mackinder B., Lock M. (2005). Tribe Dalbergieae. Legumes of the World.

[33] Polhil R.M., Polhill R.M., Raven P.H. (1981). Dalbergieae. Advances in Legumes Systematics.

[34] Wilbur R.L. (1975). A revision of the North American genus *Amorpha* (*Leguminosae*-Psoraleae). Rhodora.

[35] Estrada-Castillón E., Martínez-Ramírez J., Mares-Guerrero A.J., Ocampo G. (2020). A new outstanding species and a new section of *Dalea* (Fabaceae: Papilionoideae) from central Mexico. Phytotaxa.

[36] Cruz Durán R., Sousa M. (2005). *Eysenhardtia officinalis* (*Leguminosae*, Papilionoideae), una especie nueva de México. Novon.

[37] Cruz Durán R., Sousa M. (2013). *Eysenhardtia byei* (*Leguminosae*, Papilionoideae), una especies nueva del noroeste de México. Novon.

[38] Kalin Arroyo M.T., Polhill R.M., Raven P.H. (1981). Brongniartieae. Advances in Legumes Systematics.

[39] Grether R., Rzedowski J. (2015). *Brongniartia herbacea* (*Leguminosae*, Papilionoideae), una especie nueva de Michoacán, México. Acta Bot. Mex..

[40] Dorado O., Cruz-Durán R., Bustamente García R. (2022). Two new closely related species of *Brongniartia* (Fabaceae, Faboideae) from the Sierra Madre del Sur in Guerrero, México. Phytotaxa.

[41] Cardoso Domingos B.O.S., Mattos Cilene M.J., Filardi F., Delgado-Salinas A., Lavin M., de Moraes P.L.R., Tapia-Pastrana P., de Lima H.C. (2020). A molecular phylogeny of the pantropical papilionoid legume *Aeschynomene* supports reinstating the ecologically and morphologically coherent genus *Ctenodon*. Neodiversity.

[42] Rudd V.E. (1955). The American Species of Aeschynomene.

[43] Fernandes A. (1996). O Táxon Aeschynomene No Brasil.

[44] Särkinen T.E., Hughes C.E. (2015). Systematics and Biogeography of *Amicia* (*Leguminosae*, Papilionoideae). Syst. Bot. Monogr..

[45] Linares J., Sousa M. (2007). Nuevas especies de *Dalbergia* (*Leguminosae*: Papilionoideae: Dalbergieae) en México y Centroamérica. Ceiba.

[46] Cervantes A., Linares J., Quintero E. (2019). An updated checklist of the Mexican species of *Dalbergia* (*Leguminosae*) to aid in its conservation efforts. Rev. Mex. Biodiv..

[47] Rudd V.E. (1956). A Revision of the Genus Nissolia.

[48] Durán R., Sousa M. (2004). *Nissolia ruddiae* (*Leguminosae*, Papiliponoideae), una especie nueva de la Cuenca del Balsas, México. Acta Bot. Mex..

[49] Jansen P.I., Edye L.A. (1996). Variation within *Stylosanthes* sp. aff. *S. scabra* and comparison with its closest allies, *S. scabra* and *S. hamata*. Aust. J. Agric. Res..

[50] Vanni R.O. (2017). The genus *Stylosanthes* (Fabaceae, Papilionoideae, Dalbergieae) in South America. Bol. Soc. Argent. Bot..

[51] Gama-López S. (2006). Estudio Sistemático del Género *Stylosanthes* (Fabaceae). Master’s Thesis.

[52] Mohlenbrock R.H. (1961). A monograph of the leguminous genus *Zornia*. Webbia.

[53] INEGI (Instituto Nacional de Estadística, Geografía e Informática) (1986). Síntesis Geográfica del Estado de Nuevo León.

[54] INEGI (Instituto Nacional de Estadística, Geografía e Informática) (1983). Síntesis Geográfica del Estado de Coahuila.

[55] INEGI (Instituto Nacional de Estadística, Geografía e Informática) (1983). Síntesis Geográfica del Estado de Tamaulipas.

[56] Estrada Castillón E., Arévalo J.R., Villarreal Quintanilla J.A., Salinas Rodríguez M.M., Encina-Domínguez J.A., González Rodríguez H., Cantú Ayala C.M. (2015). Classification and ordination of main plant communities along an altitudinal gradient in the arid and temperate climates of northeastern Mexico. Sci. Nat..

[57] Rzedowski J. (1978). Vegetación de México.

[58] Estrada-Castillón E., Scott-Morales L., Villarreal-Quintanilla J.A., Jurado-Ybarra E., Cotera-Correa M., Cantú-Ayala C., García-Pérez J. (2010). Clasificación de los pastizales halófilos del noreste de México asociados con perrito de las praderas (*Cynomys mexicanus*): Diversidad y endemismo de especies. Rev. Mex. Biodiv..

[59] Jstor Global Plant Platform. https://plants.jstor.org/.

[60] Estrada-Castillon E., Villarreal-Quintanilla J.A., Cuellar-Rodriguez G., Encina-Dominguez J.A., Martinez-Avalos J.G., Mora-Olivo A., Sanchez-Salas J. (2024). The Fabaceae in Northeastern Mexico (Subfamily Caesalpinioideae, Mimosoideae Clade, Tribes Mimoseae, Acacieae, and Ingeae). Plants.

[61] Estrada-Castillón E., Villarreal-Quintanilla J.Á., Cuéllar-Rodríguez G., Torres-Colín L., Encina-Domínguez J.A., Sánchez-Salas J., Muro-Pérez G., González-Cuéllar D.A., Galván-García O.M., Rubio-Pequeño L.G. (2024). The Fabaceae in Northeastern Mexico (Subfamilies Caesalpinioideae (Excluding Tribe Mimoseae), Cercidoideae, and Detarioideae). Plants.

[62] Tropicos.org Missouri Botanical Garden. https://tropicos.org.

[63] Jarvis C. (2007). Order Out the Chaos, Linnaean Plant Names and Their Types.

[64] World Flora Online. https://www.worldfloraonline.org/.

